# Quantifying red blood cell compatibility beyond ABO and RhD: a recipient-centered model for matching, allocation, and inventory curation

**DOI:** 10.3389/fmed.2026.1875496

**Published:** 2026-07-14

**Authors:** Gennady Peysakhovich, Elena Butina

**Affiliations:** 1 Independent Consultant, Data Architect/Modeler, Feasterville, PA, United States; 2 Independent Researcher, Kirov, Russia

**Keywords:** alloimmunization, immunohematology, ImmunoHematology Framework, phenotype matching, quantitative compatibility, RBC inventory, RBC transfusion, transfusion decision support

## Abstract

**Background:**

Recipient alloimmunization remains the most frequent adverse effect of transfusion therapy. Routine transfusion decisions rely heavily on clinician judgment and informal rules, which limit consistency and reproducibility. Current transfusion decision-making practices do not systematically integrate patient-specific clinical and immunohematologic factors, phenotype-level requirements, and inventory constraints within a coherent framework. This study introduces a recipient-centered integrative model that unifies patient-group–based matching criteria, quantitative compatibility scoring, and phenotype-based inventory curation to support transfusion practices aimed at reducing alloimmunization risk.

**Methods:**

The ImmunoHematology Framework (IHF) uses demographic, clinical, and laboratory data to define phenotype-based matching requirements. It quantifies recipient–donor compatibility, calculates the degree of phenotype match using all available antigen information, and ranks recipient–unit pairs for selection and allocation. IHF also classifies donor phenotypes to support curation of refrigerated and frozen inventories. The framework was operationally evaluated as an integrated system, including its quantitative compatibility logic, using de-identified operational data and curated demonstration cases across routine and rare scenarios.

**Results:**

The IHF model produced extended phenotype matching outcomes (≥14 antigens) that demonstrated improved compatibility recommendations compared with clinician selections in the pilot evaluation, with complete agreement in 59% of cases, moderate deviation in 18%, and significant deviation in 23%. Matching 327 fully typed donors to 27 patients requiring extended antigen compatibility revealed a subset with substantially broader compatibility (median 9 vs. 4 recipients), identifying these units as candidates for long-term frozen storage.

**Conclusion:**

IHF formalizes recipient-specific transfusion risk associated with phenotype mismatch and quantitative compatibility as a computable construct the framework demonstrates how its logic, scoring method, donor unit allocation process, and phenotype-based inventory curation can be operationalized in real-world transfusion practice, with reproducibility confirmed by a functioning prototype.

## 1 Introduction

Alloimmunization is the most frequent adverse consequence of red blood cell (RBC) transfusion. It occurs when recipients are exposed to foreign antigens, typically through transfusion or pregnancy, leading to the development of antigen-specific alloantibodies and increasing the risk of hemolytic transfusion reactions or hemolytic disease of the fetus and newborn (1–3). Although the overall prevalence of alloimmunization is approximately 3% (4), rates are substantially higher in specific populations, reaching 30% in patients with sickle cell disease (SCD) (5, 6) and 20% in both thalassemia (7) and myelodysplastic syndromes (MDS) (8, 9). Individual risk is difficult to predict, but certain factors, including repeated transfusions, a history of pregnancy, and rare RBC phenotypes, substantially increase the likelihood of a post-transfusion immune response (10–12). The presence of existing alloantibodies or warm autoantibodies is also a well-documented predictor of increased post-transfusion immune risk (13, 14).

Alloimmunization complicates the identification of compatible units, increasing the risk of delayed hemolytic reactions, prolonged hospitalization, and higher healthcare costs (15, 16). Matching all clinically significant antigens could nearly eliminate antibody formation, but complete compatibility is not feasible as a universal practice due to antigenic diversity, the resource demands of extended typing, and operational constraints (17). Consequently, matching strategies must balance the risk of alloimmunization with the resources required for prevention.

Antigenic disparities between donor and recipient are the principal risk factor for alloantibody formation, and the effectiveness of prophylactic selection of compatible RBC units has been demonstrated in numerous studies showing substantial reductions in alloimmunization and its complications (18–20). In many high-income countries, prophylactic phenotype matching of donors and recipients for antigens of the Rh (CcDEe) and Kell (K) systems is routinely performed for women of reproductive age, for patients with MDS, and for recipients with autoantibodies or existing alloantibodies (21, 22). Extended matching is increasingly applied to patients requiring chronic transfusion therapy, such as those with SCD and *β*-thalassemia (23, 24), as well as to individuals receiving monoclonal antibody therapies that interfere with reliable pre-transfusion testing (anti-CD38, anti-CD47) (25).

Population-level estimates illustrate the scale and diversity of US transfusion recipients. Approximately 3.65 million individuals receive RBC transfusions annually (26). A substantial proportion requires limited or extended phenotype matching, including 500,000–900,000 women of reproductive age (27), 160,000–270,000 chronically transfused patients with SCD or MDS (28), and more than 100,000 individuals receiving anti-CD38 therapy annually (29).

The decision to provide RBC units that are phenotypically or genotypically compatible across an extended set of antigens (C, c, E, e, K, Jkᵃ, Jkᵇ, Fyᵃ, Fyᵇ, M, S, s) depends on the availability of suitable units, the clinical urgency of the transfusion, anticipated current and future transfusion requirements, and the transfusion strategy adopted by a given institution.

Large-scale donor genotyping increases the availability of RBC units with extended antigen profiles (30, 31). However, matching across a broad set of clinically significant antigens is often perceived as substantially increasing labor demands and financial costs (32, 33), leading some institutions to adopt reflexive strategies in which compatibility is limited to the ABO and RhD systems until alloantibodies are detected (6). At the same time, accumulated clinical and modeling evidence indicates that early, prophylactic phenotype matching in recipients at high risk of alloimmunization is the most effective approach for preventing primary sensitization and the subsequent cascade of alloantibody formation. Modeling studies further demonstrate that, when donors and recipients are comprehensively phenotyped or genotyped, universal extended matching based on ABO, RhD, and additional clinically significant antigens can be operationally feasible even under realistic inventory constraints (34).

Existing computational and optimization approaches relevant to transfusion practice are primarily inventory-driven, focusing on stock optimization, unit-age policies, and wastage reduction (35–37). Almost all prior inventory management models consider compatibility only for ABO and RhD and cannot be directly applied to the combinatorially more complex problem of extended antigen matching (38). A subset of mathematical models addresses extended donor–recipient phenotype matching (covering at least 14 antigens); however, these models do not stratify patients by RBC matching requirements. Binary antigen-exclusion rules applied in some approaches (34, 39) treat all “compatible” units as equivalent, resulting in an effectively random selection among acceptable units without any quantitative assessment of compatibility. Traditional fixed-antigen, all-or-nothing matching algorithms use a strict matching model that requires perfect donor–recipient antigen agreement on a predefined antigen set, substantially reducing donor availability and necessitating antigen removal and rerunning the matching process when inventory cannot meet demand (38). Other models introduce mismatch penalty formulations that assign costs to antigen differences or shortages, but they do not define recipient-specific compatibility requirements (38, 40, 41). The MINimize Relative Alloimmunization Risks (MINRAR) model (40) incorporates antigen immunogenicity (42) as the sole determinant of the clinical relevance of mismatches. A subsequent modification (41) additionally accounts for patient risk grouping; however, it still lacks a quantitative framework for ranking individual recipient–donor unit pairs and does not model the patient-specific probability of post-transfusion reactions in alloimmunized recipients.

Advances in cryopreservation and a growing emphasis on immunologic safety suggest that the traditional view of frozen RBC inventories as repositories for rarely used units should be reconsidered (43). Instead, cryopreserved inventories should be regarded as operational resources for meeting immediate clinical needs, particularly for patients requiring extended phenotype matching. Because many clinically significant phenotypes occur at low frequencies, maintaining frozen units for specific antigen profiles can help reduce delays in providing RBCs (44). Optimal use of frozen inventories requires identifying the antigen combinations most likely to be clinically necessary, calling for a systematic approach to selecting donor phenotypes that maximize clinical and operational benefits.

We developed the ImmunoHematology Framework (IHF), a unified decision support architecture that formalizes patient-specific red cell compatibility requirements, introduces a quantitative matching method, and applies compatibility information to guide allocation and inventory management. IHF integrates risk stratification, quantitative compatibility scoring, and operational decision-making across matching, allocation, and inventory management.

The framework comprises three core components: transfusion risk group (TRG), which organizes recipients according to transfusion-relevant characteristics and assigns explicit levels of phenotype matching based on expected clinical consequences of incompatible transfusion; Weighted Phenotype Compatibility Score (WPCS), which quantifies recipient–donor compatibility; and Phenotype Usage Type (PUT), which classifies donor phenotypes for inventory curation. The Antigen Significance Factor (ASF) assigns weights only when a donor–recipient antigen mismatch occurs, reflecting antigen immunogenicity and the clinical significance of corresponding antibodies (45). The IHF was validated using retrospectively loaded operational datasets processed as real-time inputs. A curated demonstration dataset was also used to illustrate rare or clinically important compatibility scenarios not well represented in routine operations. This study defines the underlying concepts and components of the IHF framework and demonstrates how its recipient-centered compatibility logic, scoring method, donor unit selection and allocation process, and phenotype-based inventory-curation approach can be operationalized to support consistent, risk-aligned transfusion decision making.

## 2 Methods

### 2.1 Glossary of terms

ImmunoHematology Framework (IHF)—A unified decision support architecture that formalizes patient-specific red cell compatibility requirements, introduces a quantitative matching method, and applies compatibility information to guide allocation and inventory curation.

Transfusion risk group (TRG)—recipient-centered classification for phenotype-based RBC compatibility that stratifies patients by demographic, clinical, and immunohematologic characteristics to define phenotype-matching requirements.

Weighted Phenotype Compatibility Score (WPCS)—unitless, rule-based quantitative method that quantifies recipient–donor compatibility across multiple antigen systems and ranks recipient–unit pairs.

Antigen Significance Factor (ASF)—predefined numeric weight applied only when a donor–recipient antigen mismatch occurs and reflects antigen immunogenicity and clinical significance.

Match Score categories (MS0/MS1/MSM)—categorical compatibility outcomes in which MS0 represents an exact match, MS1 a sufficient match, and MSM a mismatch.

Priority Factor (PF)—numeric weight that modifies categorical match outcomes at both rule and record levels and reflects the clinical and operational importance of compatibility results when prioritizing donor–recipient and unit-level matches.

Choice Factor (CF)—count of deviations from an exact match in compatible cases (MS1) or the number of mismatch rules in incompatible cases (MSM).

Choice Depth (CD)—number of antigen rules applied in compatibility evaluation.

Phenotype Usage Type (PUT)—donor-phenotype classification used for inventory curation that assigns operational categories based on antigenic composition to prioritize phenotypes likely to provide compatible units and to identify rare phenotypes.

### 2.2 IHF core concepts and components

The IHF transfusion strategy prioritizes recipient safety by defining explicit compatibility criteria. This integrative model quantifies compatibility beyond ABO/RhD and guides RBC unit selection, allocation, and phenotype-based inventory curation. The terms IHF and IHF integrative model are used interchangeably throughout.

The IHF comprises three core components: TRG, WPCS, and PUT. Together, these components support a recipient-centered approach to RBC transfusion. Additional model inputs, including ASF and deterministic match rules, support the WPCS component. The IHF logic is population-agnostic and does not depend on the demographic characteristics of the pilot dataset.

#### 2.2.1 IHF conceptual architecture

**Figure 1:**
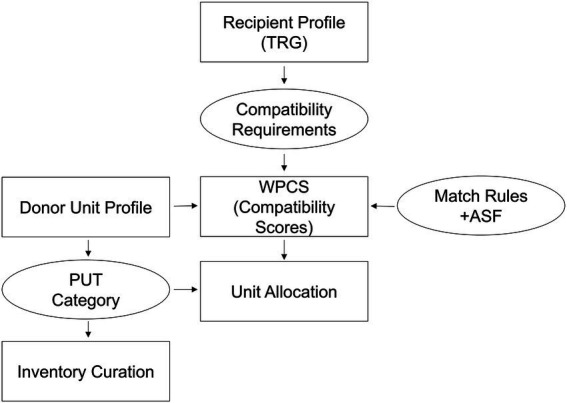
Conceptual architecture of the ImmunoHematology Framework (IHF). Recipient Profile (TRG) defines patient-specific compatibility requirements. WPCS computes compatibility scores using recipient compatibility requirements, donor antigen phenotype, and match rules. ASF is a direct input to the match-rule logic and is applied only when antigen mismatches occur. PUT classifies donor phenotypes to support phenotype-based inventory curation and, together with WPCS compatibility scores, contributes to RBC unit selection and allocation.

Figure 1 provides a high-level view of the IHF integrative model, illustrating how the framework links recipient-specific requirements, compatibility logic, scoring mechanisms, phenotype-usage classification, and inventory curation into a unified structure. This conceptual diagram clarifies how the major elements relate to one another before the detailed components are introduced.

#### 2.2.2 Transfusion risk group: patient grouping to define compatibility requirements

The TRG classification defines explicit compatibility criteria. Patients are assigned to groups based on demographic, clinical, and laboratory data. Each TRG corresponds to a phenotype-matching level (routine, limited, extended), ensuring that compatibility assessment reflects the expected clinical consequences for a recipient of incompatible transfusion. Two additional conditions are applied in specific clinical contexts.

TRG groups correspond to substantial US patient populations, including single-episode recipients (TRG1), women of reproductive age and patients with hematologic disorders (TRG2), chronically transfused individuals with SCD, thalassemia, or MDS (TRG3–5), and patients with unreliable serology (TRG6). This alignment underscores the operational relevance of a risk-based compatibility framework.

**Figure 2:**
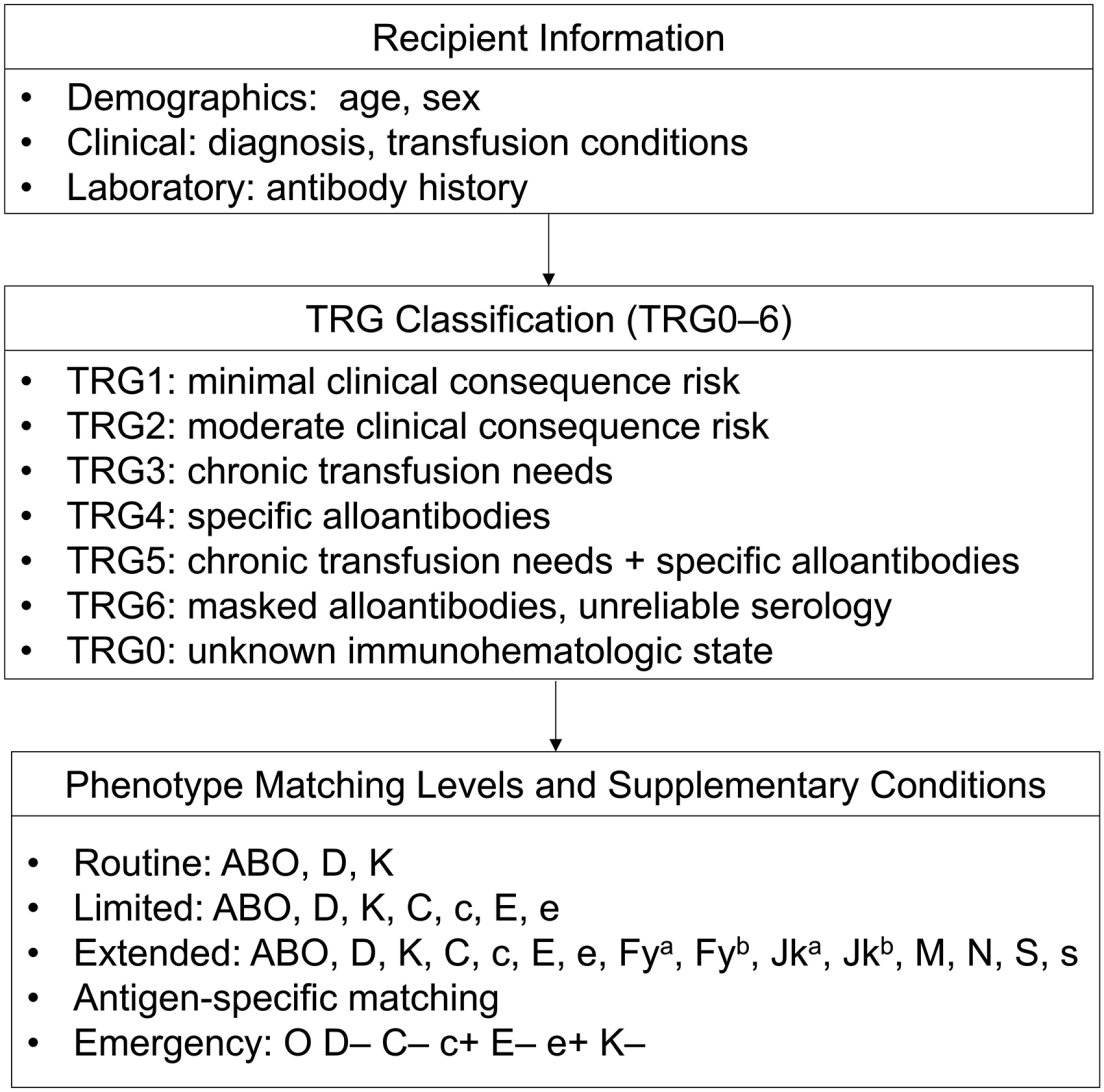
Overview of the transfusion risk group (TRG) classification. The figure summarizes recipient information used for TRG assignment, TRG categories (TRG0–TRG6), and the phenotype-matching levels and additional conditions.

Figure 2 provides a high-level overview of the TRG structure, including recipient information used for classification, TRG categories (TRG0–TRG6), and the phenotype-matching levels associated with each group.

TRG assignment generates a Recipient Profile that specifies compatibility requirements for transfusion practice. The profile summarizes key recipient-specific parameters, including:

- Demographic, clinical, and laboratory information.
- The assigned TRG.
- The required phenotype-compatibility level.
- Sufficiency of available blood-typing data.
- Notable antigen-pattern findings (rare combinations, double populations, specific autoantibodies).

Table 1 provides the detailed TRG definitions and phenotype-matching levels.

**Table 1:** Transfusion risk group (TRG) classification, recipient parameters, phenotype matching level, and example recipient.

TRG	Demographics	Clinical	Laboratory	Required compatibility (phenotype matching level)	Example recipient
1	Men; Women ≥50			Routine (ABO, D, and K)	Male 24, no antibodies
2	Women <50	HD, RT		Limited (ABO, D, K, C, c, E, e)	Female 22, no antibodies
3		MDS, SCD, THL		Extended (ABO, D, K, C, c, E, e, Fy^a^, Fy^b^, Jk^a^, Jk^b^, S, s)	Female, 61, MDS, no antibodies
4			SA	Limited (ABO, D, K, C, c, E, e)^a^	Female 56, anti-Jk^a^
5		MDS, SCD, THL	+ SA	Extended (ABO, D, K, C, c, E, e, Fy^a^, Fy^b^, Jk^a^, Jk^b^, S, s)^a^	Male 38, MDS, anti-Le^a^
6		anti-CD38/47 therapy	PA	Extended (ABO, D, K, C, c, E, e, Fy^a^, Fy^b^, Jk^a^, Jk^b^, S, s, M)	Male 62, PA
0	Unknown blood group	Emergency transfusion	Not screened	Emergency phenotype (O D- C- c + E- e + K-)	Male 30, emergency transfusion

^a^Donor units must be antigen-negative for the antigen(s) corresponding to any specific antibody detected in the recipient. HD, hematologic disorders; RT, repeated transfusions; MDS, myelodysplastic syndrome; PA, panagglutinating antibodies; SA, specific antibodies; SCD, sickle cell disease; THL, thalassemia.

A detailed description of the profile structure, creation process, and an example case is provided in Supplementary material A.

#### 2.2.3 Antigen significance factor: weighting antigen mismatches by clinical transfusion risk

The ASF assigns weights based on antigen immunogenicity and the clinical significance of the corresponding antibodies (44). ASF assigns each RBC antigen a numeric value, which is applied *only* when a donor–recipient antigen mismatch occurs. The scale is comparative: relative differences, not absolute values, determine mismatch prioritization. For illustration, ASF(C) = 200 and ASF(c) = 240 reflect the higher clinical transfusion risk associated with a c mismatch; the ASF scale used in this study and illustrative example are provided in Supplementary material B.

#### 2.2.4 Recipient–donor compatibility determination

Recipient–donor compatibility is determined by the recipient’s TRG, which specifies antigens relevant to minimizing alloimmunization. A donor is compatible when they do not express antigens absent from the recipient (donor X– → recipient X– or X+). Incompatibility occurs when the donor expresses antigens the recipient lacks (donor X + → recipient X–). Full antigenic identity represents the maximal degree of recipient–donor compatibility. Phenotype match supplements this assessment by quantifying antigen-profile similarity based on available laboratory data.

#### 2.2.5 Match rules governing donor-recipient matching

Match rules define the deterministic logic used to evaluate recipient–donor phenotype compatibility and the degree of phenotype match. Rules are grouped into two categories: antigen rules and antibody rules. ABO compatibility is enforced as a gating criterion before extended phenotype evaluation. Antigens ABO, D, and K are designated priority antigens and are handled with distinct clinical and operational logic. Antigen match outcomes follow the model’s compatibility definitions: exact match, sufficient match, mismatch, or unknown.

When recipient alloantibodies are present, antibody rules are applied. If a patient currently has, or has previously been found to have, an antibody of defined specificity, the corresponding antigen must be absent from the donor phenotype. Under antibody rules, only exact match and mismatch outcomes are permitted.

These rule-level outcomes provide the categorical and numeric compatibility measures used by the model’s scoring and allocation methods. Detailed definitions and illustrative examples are provided in Supplementary material C.

#### 2.2.6 Weighted phenotype compatibility score: quantifying recipient–donor compatibility

The WPCS is the model’s primary decision-support mechanism. It quantifies compatibility and the degree of phenotype match across multiple blood group systems and ranks RBC units while incorporating unit-specific characteristics. These functions support consistent and reproducible selection and allocation of RBC units. WPCS is a unitless, composite algorithmic method that records categorical match outcomes and applies antigen-specific match rules with assigned weights. It integrates these elements into aggregated scores and ranks (lower scores are more desirable). In addition to antigen-based compatibility, WPCS incorporates unit-specific attributes into the selection process to generate a prioritized recipient–unit matching score.

WPCS operates on three primary inputs:

- TRG: patient-level transfusion risk group defining compatibility requirements.
- Match rules: rules governing donor–recipient matching.
- ASF: antigen-specific immunogenicity and clinical significance weights.

WPCS generates four output parameters: Match Score Code (MS Code), Priority Factor (PF), Choice Factor (CF), and Choice Depth (CD), which together define compatibility. Table 2 summarizes their definitions and roles. These parameters are used across match-rule evaluation, compatibility assessment, phenotype-match quantification, and recipient–unit scoring.

**Table 2:** Parameters of the weighted phenotype compatibility score (WPCS).

Parameter	Definition	Role in WPCS
MS code	Categorical match outcome: MS0 = exact match; MS1 = sufficient match; MSM = mismatch Examples: MS0: Donor C + → Recipient C+ MS1: Donor C- → Recipient C+ MSM: Donor C + → Recipient C-	Encodes the qualitative degree of matching
Priority factor (PF)	Numeric weight that quantifies the impact of a match outcome	Measures recipient–donor compatibility by summing contributions of all applied match rules; applied to the MS Code to generate a prioritized score; determines optimal allocation among units with compatible (MS0, MS1) MS categories; guides the selection of the least hazardous incompatible units when only MSM options exist
Choice factor (CF)	For MS1: number of deviations from an exact match; for MSM: number of mismatch rules	Provides contextual detail about the nature of deviations; does not influence PF values or prioritized scores
Choice depth (CD)	Number of antigen match rules applied by the algorithm	Indicates the scope of matching used for a given comparison; does not influence PF or prioritized scores

The MS Code and PF correspond directly to clinically interpretable categories: exact matches (MS0) represent the preferred phenotype-level compatibility, sufficient matches (MS1) represent clinically acceptable alternatives, and mismatches (MSM) represent units that should be avoided unless no compatible units exist. PF values quantify the relative clinical importance of these outcomes.

#### 2.2.7 Phenotype usage type: classifying donor phenotypes for inventory curation

The PUT method supports inventory curation by classifying donor phenotypes into operational categories based on antigenic composition. It prioritizes phenotypes likely to provide compatible units for extended matching, while limiting the accumulation of rarely used units, and incorporates extremely rare phenotypes required for recipients with correspondingly rare profiles. Beyond curation, PUT also contributes to allocation by supplying phenotype-usage information to WPCS.

**Figure 3:**
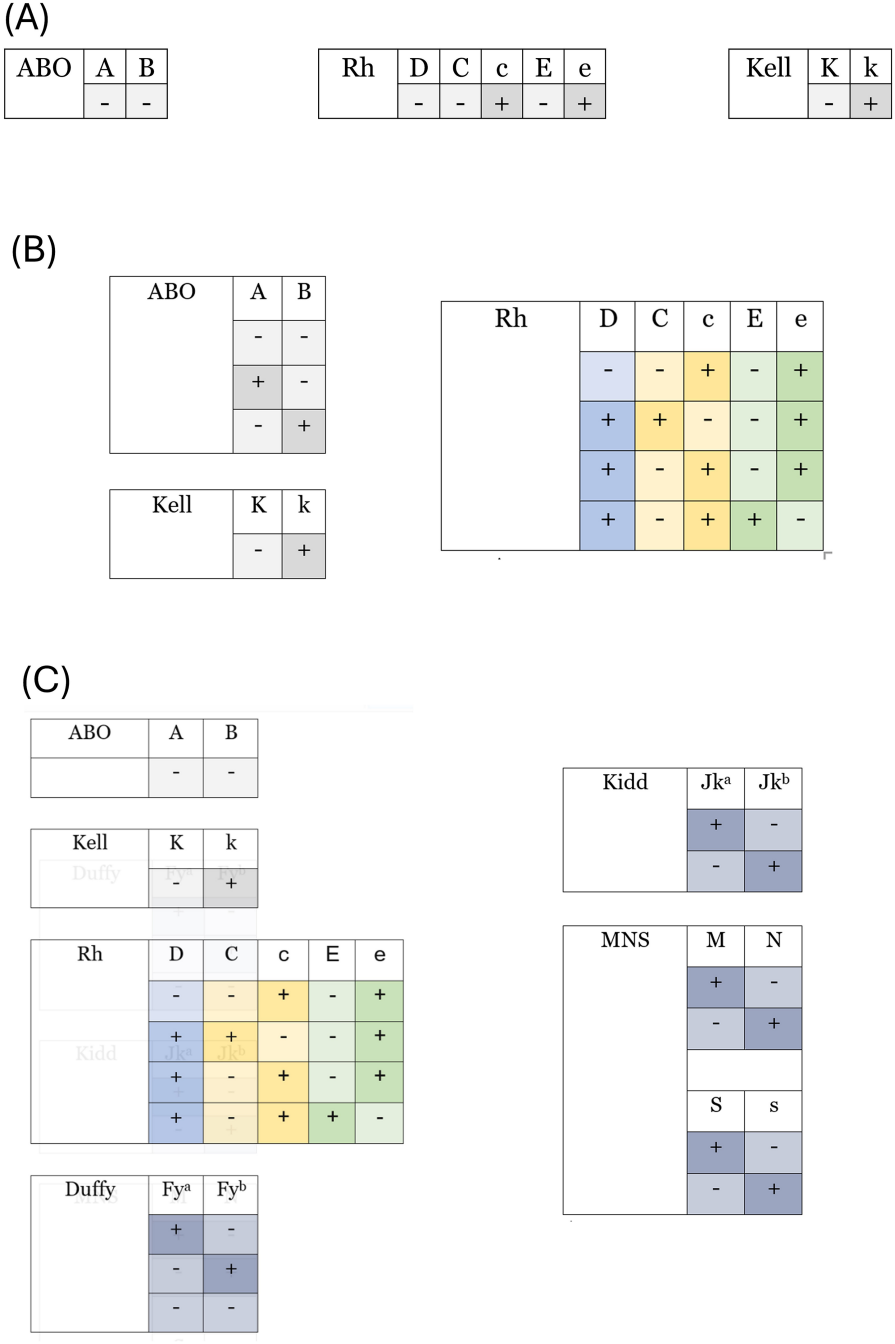
Antigen combinations defining Universal **(A)**, Required **(B)**, and Unique **(C)** PUT categories. Donor phenotypes are classified into these categories when they exhibit one of the combinations shown for each system.

PUT considers antigens from the ABO, Rh, Kell, Duffy, Kidd, MNS, and other systems. Each donor phenotype is assigned to one of five categories according to predefined criteria. Antigen combinations defining the Universal, Required, and Unique categories are shown in Figures 3A–C, and the complete classification is provided in Supplementary material D. The Unique and Extraordinary categories include units considered candidates for cryopreservation, pending the application of phenotype-usage criteria under development within the PUT framework.

### 2.3 WPCS computational architecture: rule evaluation, MS code assignment, and scoring workflow

#### 2.3.1 Architectural overview

**Figure 4:**
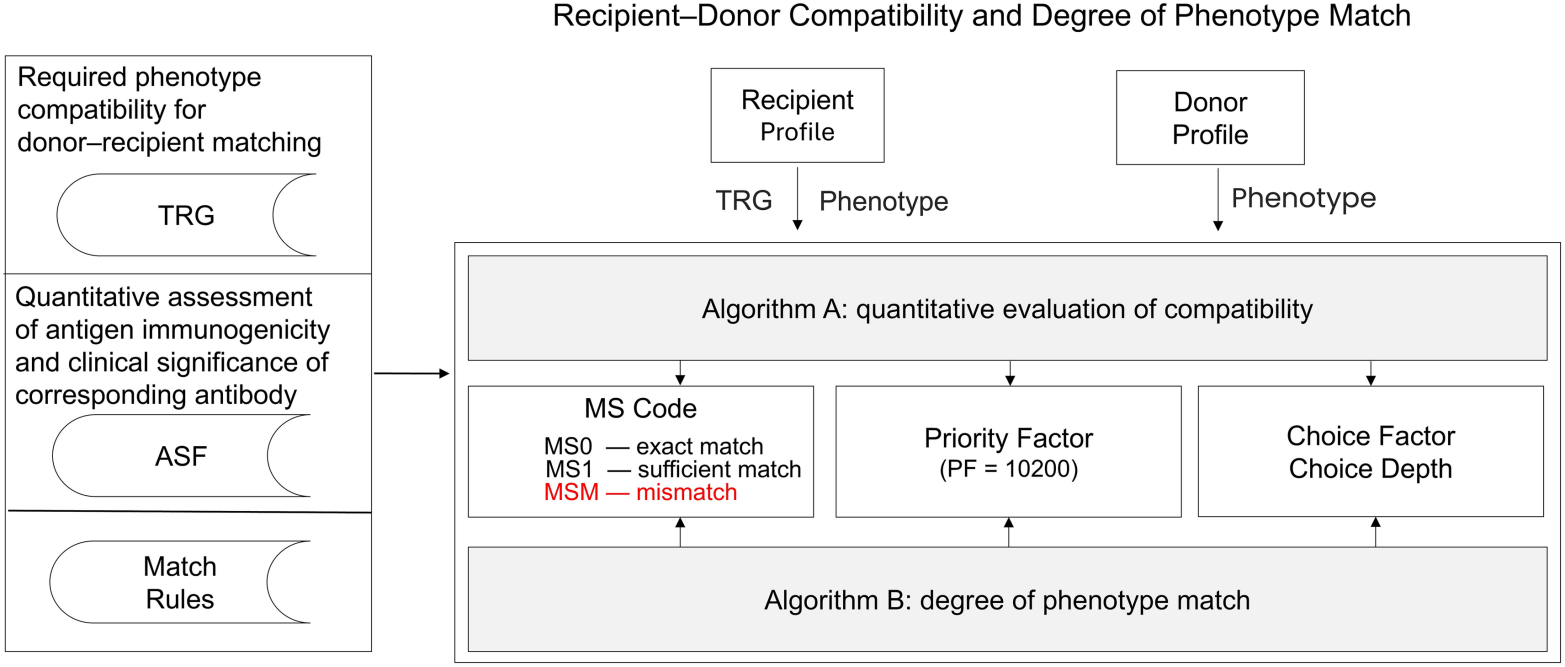
Overview of the WPCS compatibility architecture. Algorithm A (compatibility scoring) and Algorithm B (phenotype match evaluation).

The WPCS compatibility architecture (Figure 4) integrates TRG-defined requirements, antigen-specific match rules, and ASF-based immunogenicity weights to generate both compatibility and phenotype match assessments. Algorithm A evaluates recipient–donor compatibility by applying these inputs to assign MS Code (MS0, MS1, MSM), calculate PF, and determine CF and CD. Algorithm B evaluates phenotype match using the same antigen-level inputs and the same scoring structure as Algorithm A. Together, these outputs support downstream unit-level prioritization and allocation.

Compatibility architecture relies on a record-level determination of the MS Code, which serves as the starting point for all subsequent WPCS computations. WPCS assigns the MS Code at the donor-record level based on the donor’s phenotype and the recipient’s matching requirements. MS0 and MS1 are treated as distinct classes within the compatible category because an exact match (MS0) is clinically preferred over a sufficient match (MS1) and is therefore represented separately in WPCS.

#### 2.3.2 MS code: rule- and record-level categorical outcome

Rule- and record-level MS Code assignment is based on recipient–donor compatibility outcomes (exact match, sufficient match, mismatch, unknown). Each applied match rule evaluates a single antigen and returns a rule-level MS Code; these rule-level outcomes are then aggregated into a single record-level MS Code for the donor–recipient pair.

Rule-level representative examples (full rule set in Supplementary material C):

- Exact Match (MS0): Recipient C+, Donor C+
- Sufficient Match (MS1): Recipient C+, Donor C–
- Mismatch (MSM): Recipient C–, Donor C+
- Unknown: Recipient C+, Donor Null

Record-level assignment

A donor–recipient pair is assigned MS0 when all applied match rules return an exact match outcome. If the applied rules return any combination of exact match and sufficient match outcomes, the pair is assigned MS1. The presence of any mismatch rule results in MSM, and the presence of any unknowns yields Null.

Record-level representative example:

Recipient phenotype: C+, E+, M+.

Donor phenotype: C–, E+, M–.

Rule-level outcomes: MS1, MS0, MS1 → record-level MS Code = MS1.

Formally:

- MS0 applies if every rule has outcome = MS0.
- MS1 applies if any rule has outcome = MS1 and no rule has outcome = MSM.
- MSM applies if any rule has outcome = MSM.
- Null applies if any rule has outcome = Unknown.

#### 2.3.3 Priority factor: rule- and record-level numeric weight

The Priority Factor (PF) is a numeric weight that quantitatively modifies a categorical match outcome. While the MS Code identifies the qualitative match category (MS0, MS1, MSM), the PF measures and determines the outcome’s relative importance. PF values are applied independently for each match rule and modulate the matching result by adjusting the MS Code to produce a prioritized score.

There are two compatibility classes: compatible (MS0, MS1) and incompatible (MSM). While the MS Code (MS0, MS1, MSM) defines the classes themselves, the PF magnitudes differentiate outcomes within the same compatibility class.

ABO compatibility is enforced as a gating criterion before extended phenotype evaluation. Antigens ABO, D, and K are designated priority antigens and are handled with distinct clinical and operational logic. ABO compatibility is assigned a dominant PF to ensure that no combination of Rh, K, or other antigen differences can override ABO matching; D and K receive intermediate PF values reflecting their clinical significance to ensure that no combination of other antigens can override D and K matching; and other antigens contribute minimal PF values to preserve their lower relevance. The following examples illustrate how MS Code and PF interact at the rule level:

- Recipient phenotype: E+; Donor phenotype: E+

MS Code: MS0 (exact match); PF = 0

- Recipient phenotype: M+; Donor phenotype: M −
  MS Code: MS1 (sufficient match); PF = 1
- Recipient phenotype: e−; Donor phenotype: e +
  MS Code: MSM (mismatch); PF = 100 [rule ASF(e)]

PF values are proportional to antigen significance:

Exact matches (MS0):

ABO (optimal) = 10,000; *D* = 100; *K* = 100; all other antigens = 0.

- Sufficient matches (MS1): ABO = 11,000, 12,000, or 13,000 (by selection tier); *D* = 110; *K* = 110; all other antigens = 1.
- Mismatches (MSM): ABO mismatches carry the highest PF values (M, i.e., exclusion), followed by RhD (ASF = 400) and K (ASF = 360), while all other antigens contribute smaller increments based on their Antigen Significance Factor (ASF). When specific alloantibodies are present, PF values for mismatches are assigned according to antibody significance (10,000 or 15,000). These antibody-based PF values override phenotype-based PF values, ensuring that any donor unit carrying the corresponding antigen is treated as a high-priority mismatch.

Matching and scoring may be applied to any of the following outcomes: fine-grain match-rule outcomes, recipient–donor compatibility, recipient–donor degree of phenotype match, and recipient–unit selection.

The total PF for a recipient–donor pair is the sum of all rule-specific contributions, and this aggregate PF reflects the overall degree of compatibility and the degree of phenotype match. A lower Priority Factor indicates a better (more desirable) outcome.

Example (rule-level PF contributions):

A recipient with phenotype B, D+, C–, c+, E+, e–, K–, M + is compared with a donor of the same phenotype. Each antigen-level rule produces an MS0 outcome. The corresponding PF values are:

- ABO (B → B): PF = 10,000
- D (+ → +): PF = 100
- C (− → −): PF = 0
- E (+ → +): PF = 0
- e (− → –): PF = 0
- K (− → –): PF = 100

Total PF = 10,000 + 100 + 0 + 0 + 0 + 0 + 0 + 100 = 10,200.

This aggregate PF of 10,200 represents a high-quality phenotype match dominated by the ABO, D, and K contributions.

This completes the definition of the Priority Factor as the quantitative component of the rule-based matching framework. Complete descriptions and PF values for the Antigen and Antibody match rule categories are provided in Supplementary material C.

#### 2.3.4 Scoring and allocation workflow (Algorithms A and B)

The WPCS computational framework integrates the architectural components, inputs, and output parameters into a unified workflow that generates compatibility scores, phenotype-match evaluations, and ranked recipient–unit allocations. Each recipient–donor pair is evaluated using two parallel algorithms—Algorithm A and Algorithm B—which share the same rule-based structure but differ in scope and purpose. WPCS then computes a recipient–unit score for selection and allocation by adding unit-specific factor weights (Supplementary material H, section H6) to the recipient–donor compatibility score.

Algorithm A applies antigen-specific match rules (MS0, MS1, MSM) and ASF weighting across the TRG-required antigen set. The overall compatibility score is the sum of PF contributions from ABO, D/K, other antigens, and antibody-rule categories:


$$\begin{array}{l} PF_{\text{compatibility}}=\sum PF_{\mathrm{ABO}}+\sum PF_{D,K}\\ +\sum PF_{\left\{\text{other}-\text{antigens}\right\}}+\sum PF_{\left\{\text{antibody}-\text{rules}\right\}} \end{array}$$

Algorithm B uses the same match rules and ASF weighting but evaluates all available phenotype data. The resulting match score reflects the degree of phenotype similarity:


$$PF_{\text{match}}=\sum PF_{\left\{\mathrm{all}-\text{compared}-\text{antigens}\right\}}+\sum PF_{\left\{\text{antibody}-\text{rules}\right\}}$$

Both algorithms output MS Code, PF, CF, and CD; they differ only in scope and interpretation.

The system integrates phenotype-based compatibility from Algorithm A with each donor unit’s operational attributes. The overall recipient–unit score inherits the recipient–donor MS Code, and its PF value reflects contributions from both the compatibility assessment and unit-level factors, including storage method (frozen vs. fresh), PUT category, and remaining shelf-life.

The IHF uses a single, unified Priority Factor (PF) system that operates across two functional layers. Rule-level PFs weigh specific compatibility conditions within the matching rules, while unit-specific PFs (Unique PUT, rare phenotypes) (Supplementary Table H6.1) adjust allocation for individual units. Both PF types share the same PF scale and are calibrated together to ensure consistent behavior. During operational processing, the recipient–unit PF is computed as the sum of the donor-level PF and the unit-specific PF, aligning compatibility logic with allocation prioritization.

The compatibility score and unit-level factors are summed to produce a single recipient–unit score:


$$PF_{\left\{\text{recipient}-\text{unit}\right\}}=PF_{\text{compatibility}}+PF_{\left\{\text{unit}-\text{level}-\text{factors}\right\}}$$

This formulation ensures that unit selection incorporates both phenotype-based compatibility and operational suitability for transfusion.

For each recipient, all compatible units are ranked by their recipient–unit score. Mismatched units are ranked separately using the same scoring framework. The allocation algorithm prioritizes the lowest (most desirable) scores among compatible units (MS0, MS1). If no compatible units are available, the lowest-risk unit from the mismatch set (MSM) is recommended.


$$\mathrm{Ran}k_{\left\{\text{compatible}\right\}}=\text{order}(PF_{\left\{\text{recipient}-\text{unit}\right\}})$$

$$\mathrm{Ran}k_{\left\{\text{mismatch}\right\}}=\text{order}(PF_{\left\{\text{recipient}-\text{unit}\right\}})$$

$$\text{AllocatedUnit}=\min{\left(PF_{\left\{\text{recipient}-\text{unit}\right\}}\right)}_{\left\{\text{compatible}\right\}}$$

$$\text{AllocatedUni}t_{\left\{\text{mismatch}\right\}}=\min{\left(PF_{\left\{\text{recipient}-\mathrm{uni}t\right\}}\right)}_{\left\{\text{mismatch}\right\}}$$

This process produces a globally optimized, rank-ordered allocation list for each transfusion event.

### 2.4 ImmunoHematology framework: integrative model architecture

**Figure 5:**
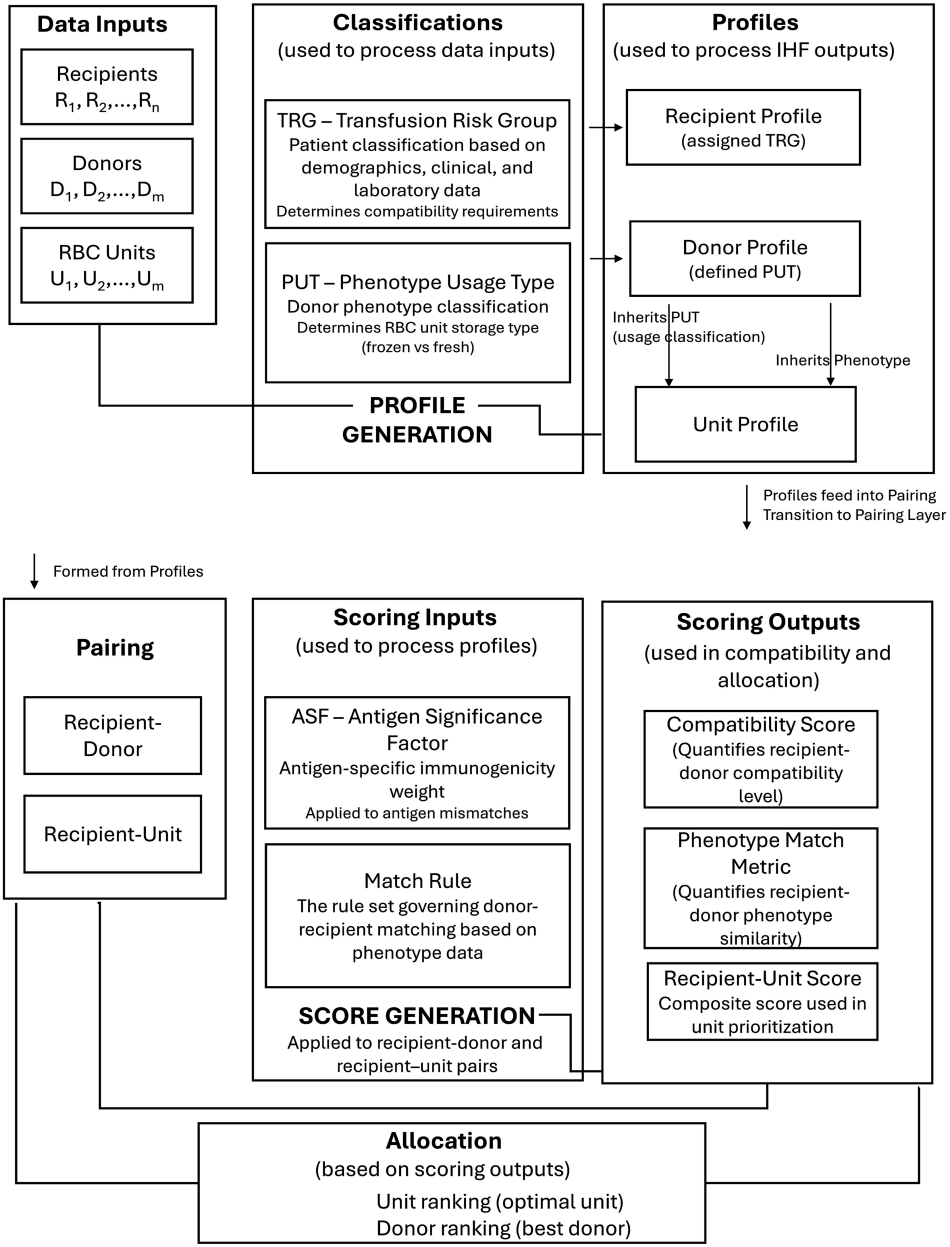
Complete conceptual architecture of the IHF integrative model.

Figure 5 presents the complete conceptual architecture of the IHF integrative model, bringing together all previously defined components into a single, coherent framework. The model shows how recipient, donor, and RBC-unit data enter the system and are transformed through TRG and PUT classifications to generate the core profiles that anchor the framework. Recipient Profiles receive TRG assignments; Donor Profiles incorporate PUT; and Unit Profiles inherit phenotype, PUT, and include unit-level attributes.

Although not depicted in Figure 5, PUT operates as a supplemental process that optimizes the antigen profile of the frozen inventory and is not required for core IHF operation.

Together with match rules, ASF, and metadata, the profiles provide WPCS with the inputs needed for compatibility evaluation, scoring, and allocation. Pairing occurs upstream, using the profiles to generate recipient–donor and recipient–unit combinations. This design makes explicit the role of each component in the framework’s operation.

### 2.5 Additional conceptual elements

#### 2.5.1 Data model

The IHF is based on a conceptual data model (Supplementary material E), with the analytical component implemented as a star-schema data mart (Supplementary material F).

#### 2.5.2 Reproducibility

The IHF methods use deterministic logic: for any given input, they consistently produce the same output. This determinism ensures that results are reproducible both theoretically and operationally, as demonstrated by the IHF prototype system.

#### 2.5.3 Adaptive framework

The IHF is designed as a metadata-driven system in which core components, including TRG definitions, PUT classifications, match rules, ASF values, and Priority Factor parameters, are configurable through metadata rather than code changes. This design allows tailoring the framework to local standards, operational policies, and phenotype-matching practices.

Because clinical practice, immunohematologic knowledge, and transfusion-related risk factors evolve over time, the IHF must incorporate new information as it becomes available. The metadata-driven architecture enables updates to compatibility requirements, antigen-matching rules, and phenotype-usage criteria without altering the underlying computational logic. This adaptability ensures that the framework remains aligned with transfusion-medicine practice.

### 2.6 Worked examples

Comprehensive worked examples illustrating the complete IHF workflow are provided in Supplementary material G. Two brief examples are included here for context.

Example 1 (TRG2 recipient, three donors):

A TRG2 recipient is matched to three donors. Donor 1 is an exact match (MS0, PF = 10,200), Donor 2 is a sufficient match (MS1, PF = 10,210), and Donor 3 is incompatible (MSM). Donor 1 is therefore the optimal choice, with the Priority Factor resolving the ordering among compatible donors.

Example 2 (Recipient–Unit ranking):

For a TRG2 recipient, five RBC units differ in MS Code and unit-level PF adjustments. Three units with MS0 and the lowest donor-derived PF (10200) are the rank-1 candidates; one MS0 unit with additional PF from a storage method or PUT classification is deprioritized; MSM units are excluded.

### 2.7 Prototype system

The IHF prototype implements the full scope of the framework presented in this article, including patient grouping, RBC unit selection and allocation, inventory analysis, and both batch and real-time processing. Supplementary material H provides an end-to-end description of this prototype.

### 2.8 Validation setup and data

To validate the IHF framework, we verify that it behaves as expected across typical and rare edge-case scenarios. Both the retrospective cohort and the demonstration dataset were processed through the prototype implementation to assess the correctness and integration of the full workflow.

The IHF pilot used a prototype implementation operating on two datasets: a de-identified operational dataset and a curated demonstration dataset. The operational dataset was obtained from a specialized clinical unit managing blood disorders. The demonstration dataset included special patient conditions and edge-case scenarios not represented in the operational data, enabling a comprehensive evaluation of the algorithmic logic and system behavior.

Real-time simulation was performed using a 61-day operational snapshot from 2020 (109 patients, 484 donors, 914 units, and 193 decisions), during which the system retrospectively generated recipient–unit compatibility assessments and allocation recommendations. An analytical evaluation using an integrated operational dataset enabled a systematic comparison of model outputs with clinicians’ decisions and a quantitative assessment of donor-phenotype usage patterns.

Representative scenarios from the curated demonstration dataset (Supplementary material J) illustrate system behavior, including TRG assignment, PUT classification, and edge-case handling.

Pattern structures were identified by querying PF, CF, and antigen data using T-SQL against the IHF prototype data mart.

### 2.9 Supplemental validation materials

Pilot validation of the IHF workflow is presented in Supplementary material I.

### 2.10 Statistical analysis

Compatibility counts and donor–recipient match distributions were non-Gaussian as indicated by visual inspection of histograms; therefore, non-parametric methods were used. Group comparisons were performed using the Mann–Whitney U test, with significance defined as *p* ≤ 0.01. These methods were selected because they are appropriate for skewed, discrete compatibility distributions and do not assume normality. Analyses were conducted on system-generated compatibility datasets exported from the IHF prototype data mart. No imputation or smoothing was applied.

The IHF is a deterministic, rule-based framework with predefined weights and fixed match rules. It contains no stochastic components and no parameters estimated from data. Accordingly, traditional statistical concepts such as probabilistic uncertainty quantification and parameter-based sensitivity analysis do not apply in the conventional sense to the IHF framework itself. Robustness in this context refers to structural stability and reproducibility: for a fixed metadata configuration and complete phenotype inputs, compatibility outcomes, Priority Factors, and resulting unit rankings remain consistent under repeated evaluation. The weighting structure (ASF, PF, MS0/MS1/MSM logic) reflects predefined quantitative weighting schemes rather than data-driven calibration.

Statistical analyses were performed using IBM SPSS Statistics, version 28.

## 3 Results

We evaluated the IHF using retrospectively loaded operational data and a curated demonstration dataset. The Results section presents the pilot validation findings, including decision quality analysis, phenotype-based compatibility evaluation, inventory-relevant donor classification, and system behavior in edge-case scenarios. The operational dataset was drawn from a specialized clinical unit managing complex immunohematologic conditions, making it representative of the intended-use population.

### 3.1 Pilot validation

The validation dataset included 109 patients, 484 donors, 914 RBC units, and 193 transfusion decisions from a specialized clinical unit managing blood disorders.

#### 3.1.1 Patient cohort

The patient cohort comprised 58 men and 51 women, aged 1–89 years. Transfusion-relevant conditions were common: 107 patients had hematologic disorders, 27 had prior daratumumab exposure, and 9 had unexpected specific alloantibodies. Smaller numbers of patients had myelodysplastic syndrome, with 6 affected; multispecific antibodies, with 6 affected; chronic myeloid leukemia, with 3 affected; or other condition-specific flags (Supplementary Table I1). These conditions were not mutually exclusive and could occur in combination. Three patients demonstrated double red cell populations consistent with chimerism. No patients had specific autoantibodies or improbable antigen combinations.

#### 3.1.2 TRG distribution

All six TRGs were represented in the cohort. TRG2 (63 patients) and TRG6 (27) were the largest groups, followed by TRG3 (9), TRG4 (8), TRG5 (1), and TRG1 (1). Phenotype information was sufficient for 108 of 109 patients; one TRG3 patient lacked extended typing for six required antigens.

#### 3.1.3 Donor cohort

The pilot included 484 donors: 270 men and 214 women. This specialized medical unit maintains a curated donor registry to ensure availability of phenotypically appropriate donors for patients with complex transfusion requirements; as a result, the donor base does not represent a random population sample.

#### 3.1.4 PUT distribution

All donors were assigned to a PUT: 233 (48%) Required, 178 (37%) Common, 58 Universal (12%), 15 (3%) Unique, and 0 Extraordinary. Donor typing depth ranged from 9 to 22 antigens (Supplementary Figure I2).

#### 3.1.5 System-level outputs

The prototype system successfully generated Recipient Profiles, Donor Profiles, and Unit Profiles for all records. TRG assignment, PUT classification, match-rule evaluation, MS Code assignment, PF computation, and recipient–unit scoring executed deterministically across the dataset. The analytics engine produced 61 daily operational snapshots and populated the data mart with compatibility assessments, phenotype-match evaluations, allocation recommendations, historical records, and aggregated operational data.

#### 3.1.6 Decision quality analysis

**Figure 6:**
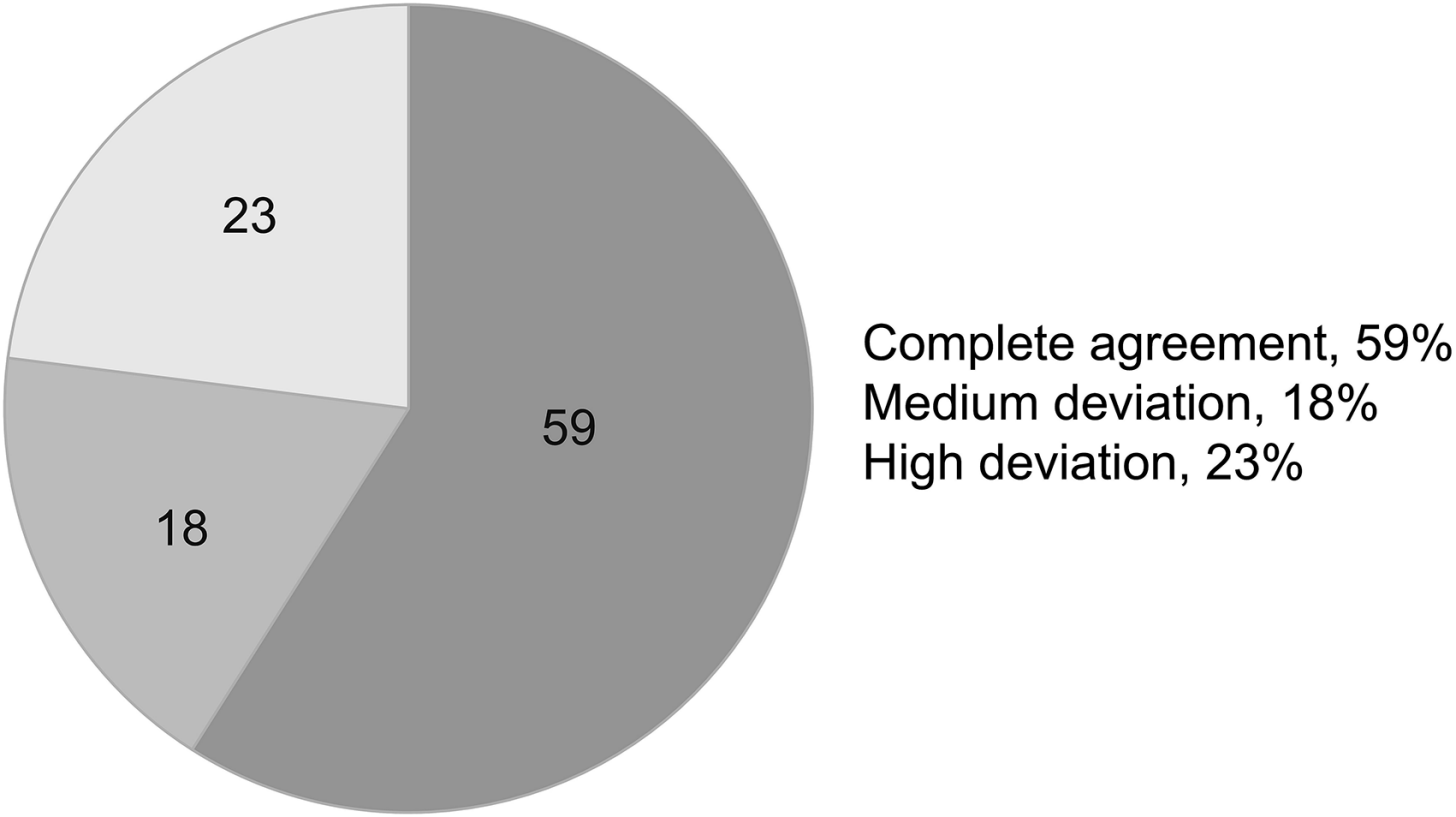
Clinician–model comparison for extended phenotype matching.

A total of 193 physician unit selection decisions were compared with model recommendations using the MS Code and PF. A retrospective comparison using quantitative compatibility scoring and unit ranking demonstrated greater alignment with phenotype-based extended matching based on the framework’s compatibility criteria than observed in clinician selections. Complete agreement was observed in 59% of cases, with moderate and significant deviation in 18 and 23%, respectively (Figure 6).

Moderate deviations reflected clinically acceptable but suboptimal choices, involving selection of a compatible unit with a higher PF. In this study, moderate deviation corresponded to cases in which the PF difference between the physician-selected unit and the model-recommended unit was 300 or less. Significant deviations reflected selection of compatible units for which the PF difference exceeded 300, indicating that the chosen unit was substantially farther from the model’s optimal recommendation while still meeting the defined compatibility requirements.

Deviation patterns fell into three scenarios. In equivalent choices, the physician and model selected units with identical MS Code and Priority Factor (MS0 and PF = 10,200). In suboptimal compatible choices, the physician selected a compatible unit with a higher PF than the model-recommended unit (Patient 22: Unit 253, PF = 10,501 vs. model Unit 239, PF = 10,201; PF difference = 300). In units excluded from model evaluation, the physician selected a unit lacking sufficient typing depth for model assessment (Patient 362: Unit 14,591 with no MS Code or PF, while the model recommended Unit 11,719, MS1, PF = 10,202). Examples correspond to cases described in Supplementary Tables I3–I6.

#### 3.1.7 Compatibility-guided frozen-inventory curation

To evaluate the framework’s ability to support compatibility-guided frozen inventory curation, we analyzed 327 donors with complete extended typing against 27 TRG 6 patients receiving daratumumab and requiring matching across the ABO, Rh, Kell, Kidd, Duffy, and MNS systems. Donors classified as Unique PUT were compatible with significantly more TRG6 recipients than donors classified as Common, Required, or Universal (median 9 vs. 4; *p* ≤ 0.01; average 9.1 vs. 3.8, SD 2.38 vs. 2.62) (Table 3). These findings confirm that Unique PUT donors represent high-value extended-match phenotypes and are appropriate candidates for long-term storage.

**Table 3:** Donor–recipient compatibility by phenotype usage type (PUT) for TRG 6 patients.

Donor phenotype usage type (PUT)	Donors (n)	Compatible patients (n)		*p*
		Average ± standard deviation	Median (range of values)	
Unique	15	9.1 ± 2.38	9 [3–12]	≤0.01
Common, required, universal	312	3.8 ± 2.62	4 [0–12]	

### 3.2 System behavior in edge-case scenarios

System behavior was evaluated using representative scenarios from the curated demonstration dataset (Supplementary material J). These examples illustrate how the model handles atypical, incomplete, or internally inconsistent phenotype information.

In scenarios involving double populations (S2V3), the system interprets DP antigens as absent for compatibility purposes, maintaining deterministic matching despite an atypical serologic pattern. When a reported phenotype includes combinations with zero or near-zero population frequency, such as the E–e– constellation in S2V4, the system issues a warning and recommends confirmatory testing, supporting phenotype quality assurance and preventing the propagation of erroneous data. The model also identifies cases in which phenotype information is insufficient for the assigned transfusion risk group. For example, in S3V3, the absence of Jk^a^ and Jk^b^ typing is explicitly detected, and the system provides actionable next steps, including retyping or a fallback to model-based matching using available information. In situations where an antibody is directed against an antigen expressed by the patient, such as anti-e in an e-positive phenotype (S4V4), the finding is classified as a specific autoantibody rather than an alloantibody incompatibility.

These edge-case scenarios demonstrate that the system applies deterministic, rule-driven logic even when phenotype information is incomplete, atypical, or internally inconsistent.

### 3.3 Framework coherence and integrated operation

Across both the operational and demonstration datasets, the integrated operation of TRG assignment, WPCS scoring, PUT classification, and the analytics engine confirmed the framework’s coherence, producing consistent, deterministic outputs. Compatibility assessments, phenotype match evaluations, and recipient–unit rankings aligned with clinical expectations and revealed opportunities to improve decision quality and inventory management. Pilot objectives, detailed results, scenario-level analyses, and full tables and figures are provided in Supplementary material I. Extended validation results, including demonstration-dataset scenarios and edge-case analyses, are provided in Supplementary material J.

### 3.4 Compatibility patterns across TRG groups

Presents the population-level compatibility landscape defined by extended-phenotype information across TRG categories. One pilot patient with a K– phenotype had no compatible donors in the operational dataset, and another had insufficient phenotype information for donor selection. Both patients were excluded from the compatibility result set but were present in the mismatch data.

#### 3.4.1 Compatibility counts

Compatibility counts across TRG groups are summarized in Table 4. Counts of MS0 and MS1 compatible pairs, together with CF averages and maxima, are shown for each TRG. CF quantifies the number of sufficient-match antigens (MS1 outcomes) and reflects deviation from an exact match within the compatible category. TRG1 showed the broadest compatibility; TRG2 contributed the largest absolute number of compatible pairs; TRG4 showed moderate compatibility; TRG3 and TRG6 had limited compatible donors with higher deviation counts; and no compatible donors were identified for TRG5.

**Table 4:** Compatible patient–donor pairs across TRG groups.

TRG	Patients	Patient– donor pairs	% of pairs within TRG	MS0	MS1	Choice factor Avg	Choice factor max
1	1	324	67	70	254	0.98	2
2	63	11,880	39	1,553	10,327	1.83	4
3	8	172	5	3	169	2.62	5
4	8	528	17	54	474	1.79	4
5	0	0	0	0	0	0	0
6	27	1,301	11	19	1,282	3.31	7

#### 3.4.2 PF distributions

Residual PF distributions at the record level (excluding ABO, D, and K) showed non-zero PF values for all TRGs with patients, with medians ranging from 1 to 2 and IQRs spanning 0–1 to 1–3. Variability remained modest (SD ≈ 0.64–1.03). PF values in Table 5 represent record-level residual PF outcomes aggregated by TRG. PF > 0 occurred in 69.1–95.6% of records in TRGs 2–4 and 6, reflecting the accumulation of multiple sufficient-match (MS1) antigen-rule contributions within this compatible dataset; PF = 0 appeared only in TRGs without patients.

**Table 5:** Residual PF distribution by TRG (record level, excluding ABO, D, and K).

TRG	Patients	Median PF	IQR (0.25–0.75)	SD	PF = 0 count	PF>0 count	PF>0%
1	0	0	0–0	0	0	0	0
2	63	1	0–1	0.7	3,666	8,214	69.1
3	8	2	1–2	0.8	9	163	94.7
4	8	1	0–1	0.64	133	395	74.8
5	0	0	0–0	0	0	0	0
6	27	2	1–3	1.03	57	1,244	95.6

## 4 Discussion

### 4.1 Interpretation of pilot findings

The pilot assessment yielded several important observations. Across the validation cohort, the IHF model showed greater alignment with phenotype-based extended matching according to the framework’s compatibility criteria than clinician selections.

It identified medically compatible units with inventory management–relevant characteristics, prioritized units with lower PF values when multiple compatible options were available, and consistently flagged cases where insufficient typing depth limited compatibility assessment. Together, these findings illustrate how the framework supports consistent application of compatibility logic, provides transparency in unit selection, and applies its allocation algorithm (Supplementary material I).

A central reason for this performance is the framework’s explicit definition of compatibility requirements. The TRG classification establishes the appropriate antigen-matching scope for each patient before any unit is evaluated, eliminating variability in manual workflows, where clinicians must integrate transfusion-relevant conditions, antibody history, and phenotype expectations without a formal structure. Building on TRG, the model evaluates all available units simultaneously and applies deterministic compatibility, phenotype depth, and prioritization rules, yielding model-generated selections that differed from physician selections in 41% of cases, including 27 high-deviation choices (PF > 300). The system also enforces phenotype sufficiency, flagging cases in which insufficient typing depth limited compatibility assessment, as observed in 9% of clinician selections, and preserving Unique PUT units when equally compatible alternatives existed. These structural features explain how the model generated selections with greater alignment to the framework’s compatibility criteria under the complexity of extended phenotype matching.

The PUT classification further demonstrated value for inventory curation. Unique donors were compatible with substantially more recipients than other PUT categories, identifying them as high-value units and underscoring PUT’s role in selecting phenotypically distinctive donors for cryopreservation.

### 4.2 Compatibility patterns—conclusion

The compatibility patterns across TRGs confirm that the TRG structure partitions patients into groups with progressively narrower donor pools, reflecting the underlying antigen requirements of each group. TRG2 showed broad donor availability; TRG3, TRG4, and TRG6 exhibited constrained compatibility, consistent with their extended phenotype scopes. TRG assignment, therefore, functions as a practical predictor of donor scarcity and match complexity.

Record-level PF values increased in TRGs with more restrictive antigen scopes, reflecting the accumulation of multiple MS1 contributions within the compatible set. This behavior confirms that PF provides a monotonic and interpretable measure of phenotype divergence for compatible recipient–donor pairs. In practical terms, PF measures phenotype divergence within the compatible set without implying clinical risk.

The observed compatibility landscape mirrors real-world experience: limited-scope groups such as TRG2 have broad donor access, while more complex phenotypes (TRG3–6) require selective donor pools. These TRG-level patterns support the IHF allocation method, particularly for groups with narrow compatibility windows, where selecting the lowest-PF-compatible unit provides a more consistent match than *ad hoc* selection.

Although comprehensive 14-antigen typing and matching are technically feasible, compatibility patterns across TRGs indicate that extended-match phenotypes are intrinsically rare because multi-antigen combinations occur at low frequencies. In the validation cohort, donor pools narrowed substantially as antigen requirements increased, particularly for TRG3–6. A uniform extended-matching strategy would therefore misallocate these low-frequency phenotypes to recipients who do not require them, reducing availability for high-risk groups with narrow phenotype requirements. The TRG classification corrects this imbalance by aligning matching depth with clinical transfusion risk and donor pool scarcity, ensuring that extended-match phenotypes are preserved for recipients who depend on them and that extended matching remains operationally feasible.

### 4.3 Conceptual contributions

This study introduces quantitative compatibility as a formal concept in transfusion medicine and provides the operational demonstration that such a framework is feasible, coherent, and clinically interpretable. The IHF is not merely an algorithmic improvement but a proof-of-concept implementation showing that compatibility, patient-specific risk, and phenotype-level comparison can be defined, quantified, and operationalized within a unified decision structure. By demonstrating that quantitative compatibility can be computed reproducibly and applied to patient data and inventory, the study provides a foundation for further evaluation of a quantitative, recipient-centered approach to transfusion practice.

Building on this foundation, quantitative compatibility reframes transfusion matching and allocation as quantitative optimization problems rather than rule-based filtering tasks. By representing recipient–donor relationships as measurable compatibility, the framework enables ranking, prioritization, and phenotype-level comparison that are not achievable within binary, rule-based antigen-exclusion models.

The framework introduces conceptual contributions that address gaps not fully resolved by existing approaches. Prior work has focused on expanding donor genotyping or optimizing allocation algorithms, but these efforts operate without a unified, clinically interpretable compatibility logic. IHF addresses this gap by providing a structured method for defining recipient-specific compatibility requirements and organizing phenotype-based inventory, supplying the compatibility framework that is not present in current matching and allocation strategies.

The pilot results illustrate how this structure enables a coherent, compatibility-driven approach to allocation and inventory management. By defining and quantifying compatibility requirements, the recipient-centered model provides a principled basis for extended matching, and offers a structured foundation consistent with precision-oriented transfusion practice. This quantitative compatibility framework clarifies the rationale for each recommendation and strengthens the interpretability of phenotype-based decisions.

Current transfusion practice relies heavily on clinician judgment and informal decision rules, which can limit consistency and reproducibility. IHF formalizes compatibility assessment through explicit compatibility rules and quantitative scoring, providing a structured alternative to *ad hoc* processes while remaining compatible with existing clinical workflows. This shift from judgment-based to model-based reasoning enables consistent application of compatibility logic and enhances the transparency of phenotype-guided decisions.

Within this architecture, the PUT method contributes a principled approach to phenotype-guided inventory curation. By identifying donors with broad recipient compatibility, PUT informs allocation logic and supports the strategic preservation of phenotypically distinctive units.

### 4.4 Related work and comparison to existing approaches

Current transfusion practice relies on qualitative, rule-based approaches that typically do not incorporate recipient-specific transfusion risk or quantitative assessment of compatibility. Allocation decisions are dominated by operational policies such as first-in, first-out or least-shelf-life-first-out, and by manual antigen-matching rules applied to selected patient groups. Even when computational tools are used, such as age-optimization models or inventory-forecasting systems, these methods primarily focus on supply, age, and wastage, rather than compatibility. Rare-donor and phenotype-matching systems expand access to antigen-negative units but generally remain binary, treating all antigen-negative units as equivalent and offering limited mechanisms to rank units or quantify antigenic similarity.

Existing approaches incorporate elements of recipient-specific matching or optimization, but do not define a unified, recipient-centered compatibility framework that classifies patients by transfusion risk and provides a quantitative method for ranking recipient–donor pairs. In current practice, antigen matching is typically based on antigen-by-antigen exclusion, where units meeting the required negative antigens are treated as equally suitable. This approach does not readily distinguish between units with different degrees of antigenic compatibility, does not generalize well across complex serologic patterns, and does not incorporate patient-specific alloimmunization risk.

The IHF addresses these gaps by integrating patient-specific risk classification with a quantitative compatibility score, enabling reproducible, recipient-centered matching that complements existing operational workflows. By defining compatibility requirements through the TRG and quantifying compatibility through the WPCS, the IHF provides deterministic, transparent, and clinically interpretable compatibility logic that is not provided by current qualitative or inventory-driven systems.

Table 6 summarizes the major distinctions between existing practice and IHF in compatibility logic, risk modeling, matching strategy, and operational integration.

**Table 6:** Comparison of current transfusion practice and the IHF framework across key operational and immunohematologic dimensions.

Dimension	Current practice	IHF
Compatibility definition	Binary (compatible/incompatible); rule-based	Quantitative compatibility score (WPCS)
Recipient-specific risk	Not modeled. All patients are treated equally, except for special groups (e.g., SCD)	TRG classifies patients by transfusion risk and defines compatibility requirements
Antigen-specific matching	Manual negative rules (e.g., C– E– K–); no scoring	Algorithmic, phenotype-level compatibility scoring
Extended antigen matching	MINRAR-type binary antigen-by-antigen matching; penalty-based	Unified compatibility scoring (WPCS) across all units
Ranking of RBC units	None; any compatible unit considered equivalent	Full ranking of all units for each recipient
Operational integration	First-in, first-out (FIFO)/least-shelf-life-first-out age policies; age availability trade-offs	Integrates risk + compatibility + operational constraints
Inventory optimization	Separate forecasting/stock models; not linked to matching	Can operate on top of inventory models to guide allocation
Decision reproducibility	Qualitative, variable between technologists	Deterministic, reproducible
Scope	Local, rule-based, qualitative	Global, quantitative, recipient-centered

### 4.5 Limitations and directions

The IHF framework has several limitations. Its performance depends on the completeness and accuracy of demographic, clinical, and laboratory inputs. Patient grouping and compatibility scoring reflect current immunohematology knowledge, which may evolve. Although IHF is metadata-driven and adaptable to changing standards, its present validation is limited.

The validation strategy used in this study was designed to evaluate the behavior and reproducibility of the framework in a controlled setting. The dataset was derived from a dedicated clinical unit in which patients uniformly require extended antigen matching and the donor pool is comprehensively typed across multiple antigen systems. This environment provides the level of phenotypic completeness and matching complexity necessary to demonstrate the operation of the quantitative compatibility logic.

However, the evaluation was conducted at a single institution using a curated, non-random donor registry and may not capture the diversity of transfusion service practices or donor populations. This may limit external generalizability and introduce potential selection bias. Moreover, IHF is a conceptual model with operational validation, and this study does not evaluate clinical outcomes. These limitations highlight the need for multi-institutional validation before broader application.

Future evaluation across diverse clinical settings is essential to assess the performance of patient grouping, compatibility logic, and scoring methods in real-time workflows. Integration with laboratory information systems and electronic health records may enable automated data ingestion and enhance operational consistency. As these developments progress, the IHF framework may provide a scalable foundation for individualized compatibility assessment, allocation-oriented ranking, and phenotype-informed inventory curation.

## 5 Conclusion

The IHF provides a structured, clinically interpretable compatibility framework that addresses long-standing gaps in phenotype-guided transfusion practice. By defining recipient-specific compatibility requirements and applying quantitative scoring and ranking, the model supports allocation that best satisfies clinically defined phenotype-matching requirements. The pilot evaluation demonstrates the feasibility of a quantitative compatibility approach and highlights its potential to improve decision quality and inventory curation while supplementing current practice. Broader validation across diverse clinical settings is needed to confirm scalability and to support integration into routine workflows.

## Data Availability

This study used retrospective, fully de-identified operational transfusion data obtained from an immunohematology laboratory in Russia (2020). The data are fully de-identified but cannot be publicly released or deposited in a repository. Access was granted to the authors for the purposes of this analysis only, and further distribution is not permitted. Requests to access the dataset may be directed to the corresponding author via the manuscript’s correspondence email address. Due to limitations on dataset sharing, access cannot be granted beyond the scope permitted by the originating laboratory.

## Author’s note

Some concepts, text passages, and illustrative elements in this manuscript, while substantially revised, were adapted from material previously published by the authors in a self-published book-length work, *ImmunoHematology Framework (IHF) (2022)*, intended for practitioners and system designers. The present manuscript is a distinct scientific work that presents a formal quantitative compatibility model, including its mathematical formulation, modeling framework, and data analysis.
