# Supplementary material for: Quantifying red blood cell compatibility beyond ABO and RhD: a recipient-centered model for matching, allocation, and inventory curation
Source: Front Med (Lausanne). 2026 Jul 14;13:1875496. doi: 10.3389/fmed.2026.1875496 (PMC13407175; doi:10.3389/fmed.2026.1875496)
Supplement: Supplementary file 1 [file Data_sheet_1.pdf]

Supplement A. Recipient Profile

This supplement provides the structure and example of the Recipient Profile.

The Recipient Profile is developed by mapping recipient data to TRG parameters. The primary focus is the required level of phenotype compatibility for donor RBC selection (Figure A1).

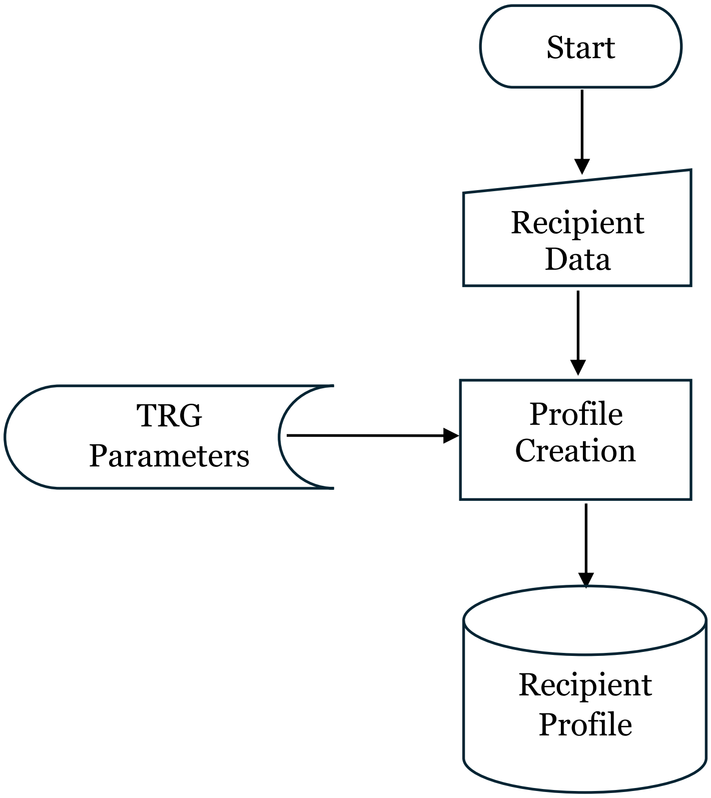

Figure A1 Recipient profile: creation process for the recipient profile used in IHF matching and patient classification.

The Recipient Profile includes the following elements:

- Demographics, clinical, and laboratory information — includes relevant reported ICD[1] codes.
- Assigned TRG — determined by matching patient information to TRG parameters.
- Required phenotype-compatibility level — required level of phenotype compatibility for RBC donor selection.
- Blood typing sufficiency — evaluated against TRG requirements (see Table A1 for example).
- Impossible, improbable, and rare antigen combinations.
- Double Population (DP) condition — the simultaneous presence of two distinct red-cell populations in a patient's sample.
- Autoantibody status — presence and, if available, specificity and date of detection.

Table A1 Example case: recipient, TRG3. Insufficient information for RBC selection.

TRG	3
Compatibility requirements	ABO, D, K, C, c, E, e, Fy^a, Fy^b, Jk^a, Jk^b, S, s
Phenotype	ABO, D, K, C, c, E, e, Fy^a, Fy^b, S, s
Information	Insufficient information for RBC selection:<br>The patient's phenotype lacks Jk^a and Jk^b results required for TRG 3 matching; additional typing is needed to enable antigen-specific donor selection.

[1] International Statistical Classification of Diseases and Related Health Problems
