# Supplementary material for: Quantifying red blood cell compatibility beyond ABO and RhD: a recipient-centered model for matching, allocation, and inventory curation
Source: Front Med (Lausanne). 2026 Jul 14;13:1875496. doi: 10.3389/fmed.2026.1875496 (PMC13407175; doi:10.3389/fmed.2026.1875496)
Supplement: Supplementary file 2 [file Data_sheet_10.pdf]

Supplement J. Demonstration dataset used to illustrate IHF system behavior.

This dataset contains curated patient and donor scenarios designed to demonstrate TRG assignment, PUT classification, system messages, and edge-case behavior. These records and scenarios are not derived from clinical records.

Contents

Supplement J. Demonstration dataset used to illustrate IHF system behavior. 1
Patient Profiles 2
Donor Profiles 17
Blood Unit Profiles 24
Donor-Patient Match 31
Patient-Unit Selection Choice Ranking and System Recommendations 41

Patient Profiles

1. Patient demographic, medical, and laboratory information received.
2. If applicable Transfusion Condition is determined.
3. Transfusion Risk Group (TRG) assigned.
4. Blood typing information sufficiency determined.
5. System messages generated.

Scenario: #1 (S2V1)

Description:
Patient Transfusion Risk Group 2, sufficient information for donor selection, no special and informational messages

Patient name: Demo Patient
Medical facility patient ID = PDS2V1TRG2, source patient ID = 1
Patient demographics: gender: male, age: 31, race: white

Patient medical conditions: BD (Blood hematological disorders)
Patient phenotype: B, D-C-c+E-e+K- (choice depth = 7, blood type: B-)

IHF assigned Transfusion Risk Group: 2
TRG is assigned based on BD transfusion condition
TRG choice depth recommendation = 7 (ABO, D, C, c, E, e, K)

IHF Messages:
Sufficient phenotype information available for donor selection
Special and informational messages: N/A

Comments: N/A

Scenario: #10 (S3V3)

Description:
Patient Transfusion Risk Group 3, insufficient information for donor selection, special and informational messages

Patient name: Demo patient
Medical facility patient ID = PDS3V3TRG3, source patient ID = 10
Patient demographics: gender: female, age: 61, race: white

Patient medical conditions: ICD10 reported C92.2 (Atypical chronic myeloid leukemia, BCR/ABL negative)

Patient phenotype: O, D+C-c+E+e+Cw-K-k+, Fy(a+b+), M+N+S+s+ (choice depth = 15, blood type: O+)

IHF determines Transfusion Condition using ICD10 reported (C92.2).
Transfusion Condition is CML - Chronic myeloid leukemia.

IHF assigned Transfusion Risk Group: 3
TRG is assigned based on CML transfusion condition
TRG choice depth recommendation = 13 (ABO, D, C, c, E, e, K, Fy^a, Fy^b, Jk^a, Jk^b, S, s)

IHF Messages:

Insufficient phenotype information available for donor selection

Special and informational messages:
Missing antigen Jk^a
Missing antigen Jk^b

Comments:

Options:

- Possible manual RBC unit selection using IHF "model 2 (all available information)" patient to donor match.
- Perform patient Kidd (Jk) blood typing and update patient blood typing information.

Scenario: #2 (S2V2)

Description:
Patient Transfusion Risk Group 2, sufficient information for donor selection, no special and informational messages

Patient name: Demo Patient
Medical facility patient ID = PDS2V2TRG2, source patient ID = 2
Patient demographics: gender: female, age: 22, race: white

Patient medical conditions: N/A
Patient phenotype: O, D-C-c+E-e+K- (choice depth = 7, blood type: O-)

IHF assigned Transfusion Risk Group: 2
TRG is assigned based on Female, younger than 55
TRG choice depth recommendation = 7 (ABO, D, C, c, E, e, K)

IHF Messages:
Sufficient phenotype information available for donor selection
Special and informational messages: N/A

Comments: N/A

Scenario: #3 (S2V3)

Description:
Patient Transfusion Risk Group 2, sufficient information for donor selection, no special and informational messages

Patient name: Demo patient
Medical facility patient ID = PDS2V3TRG2, source patient ID = 3
Patient demographics: gender: female, age: 62, race: white

Patient medical conditions: BD (Blood hematological disorders)
ICD3 reported C91.0 (Malignant neoplasms, stated or presumed to be primary, of lymphoid, haematopoietic and related tissue)

Patient phenotype: A, D(DP)C+c+E(DP)e+K- (choice depth = 7, blood type: O D(DP)

IHF assigned Transfusion Risk Group: 2
TRG is assigned based on BD transfusion condition
TRG choice depth recommendation = 7 (ABO, D, C, c, E, e, K)

IHF Messages:
  Special and informational messages:
  Double population (Chimerism). See patient phenotype information
  Sufficient information available for donor selection

Comments:
  Options:
Двойная популяция (DP) эритроцитов при типировании пациента не является препятствием для подбора совместимых доноров, так как в IHF используется алгоритм, при котором «химерный» антиген считается «отсутствующим» в фенотипе реципиента. В данном примере, несмотря на наличие DP по антигенам D и E, имеющейся информации достаточно для подбора совместимых единиц эритроцитов.

Scenario: #4 (S2V4)

Description:
Patient Transfusion Risk Group 2, insufficient information for donor selection, special and informational messages

Patient name: Demo patient
Medical facility patient ID = PDS2V4TRG2, source patient ID = 4
Patient demographics: gender: male, age: 64, race: white

Patient medical conditions: BD (Blood hematological disorders)

Patient phenotype: B, D+C+c+E-e-K-k+ (choice depth = 8, blood type: B+)
IHF assigned Transfusion Risk Group: 2
TRG is assigned based on BD transfusion condition

TRG choice depth recommendation = 7 (ABO, D, C, c, E, e, K)

IHF Messages:

Informational messages:
Insufficient phenotype information available for donor selection. Yes!

Waring messages:
Improbable or very rare antigen combination. Verify phenotype. Phenotype = E-e- frequency is ZERO

Comments:

Options:

1. Possible manual RBC unit selection using IHF "model 2 (all available information)" patient to donor match.
2. Perform patient E(Rh) and e(Rh) blood retyping and update patient blood typing information.

Scenario: #5 (S0V1)

Description:
Patient Transfusion Risk Group 0, sufficient information for donor selection, no special and informational messages

Patient name: Demo Patient
Medical facility patient ID = PDS0V1TRG0, source patient ID = 5
Patient demographics: gender: male, age: 20, race: white

Patient medical conditions: N/A

Patient phenotype: N/A

IHF assigned Transfusion Risk Group: 0
TRG is assigned based on Immediate transfusion

IHF Messages: Sufficient Information Available For Donor Selection.
Special and informational messages: N/A

Comments:

1. Immediate transfusion, patient phenotype is unknown. Donor phenotype must be O, D-C-c+E-e+, K- (O,ccdee,K-)
2. Perform patient blood typing and update patient demographic, medical and laboratory information.

Scenario: #6 (S1V1)

Description:
Patient Transfusion Risk Group 1, sufficient information for donor selection, no special and informational messages

Patient name: Demo Patient
Medical facility patient ID = PDS1V1TRG1, source patient ID = 6
Patient demographics: gender: male, age: 24, race: white

Patient medical conditions: N/A
Patient phenotype: A, D-K- (choice depth = 3, blood type: A-)

IHF assigned Transfusion Risk Group: 1
TRG is assigned based on Male who possess no negative transfusion conditions
TRG choice depth recommendation = 3 (ABO, D, K)

IHF Messages:
Sufficient phenotype information available for donor selection
Special and informational messages: N/A

Comments: N/A

Scenario: #7 (S1V2)

Description:
Patient Transfusion Risk Group 1, sufficient information for donor selection, no special and informational messages

Patient name: Demo Patient
Medical facility patient ID = PDS1V2TRG1, source patient ID = 7
Patient demographics: gender: female, age: 71, race: white

Patient medical conditions: N/A
Patient phenotype: B, D+C-c+E+e-K-k+ (choice depth = 8, blood type: O+)

IHF assigned Transfusion Risk Group: 1
TRG is assigned based on Female older than 55 who possess no negative transfusion conditions
TRG choice depth recommendation = 3 (ABO, D, K)

IHF Messages:
Sufficient phenotype information available for donor selection

Special and informational messages: N/A

Comments: N/A

Scenario: #8 (S3V1)

Description:
Patient Transfusion Risk Group 3, sufficient information for donor selection, no special and informational messages

Patient name: Demo Patient
Medical facility patient ID = PDS3V1TRG3, source patient ID = 8
Patient demographics: gender: female, age: 63, race: white

Patient medical conditions: BD (Blood hematological disorders)
ICD10 reported D46 (MDS - Myelodysplastic syndrome)

Patient phenotype: B, D+C+c+E-e+K-k+, Fy(a+b+), Jk(a-b+), M-N+S-s+ (choice depth = 16, blood type: B+)

IHF determines Transfusion Condition using ICD10 reported (D46).
Transfusion Condition is MDS - Myelodysplastic syndrome.

IHF assigned Transfusion Risk Group: 3
TRG is assigned based on MDS transfusion condition
TRG choice depth recommendation = 13 (ABO, D, C, c, E, e, K, Fy^a, Fy^b, Jk^a, Jk^b, S, s)

IHF Messages:
Sufficient phenotype information available for donor selection
Special and informational messages: N/A

Comments: N/A

Scenario: #9 (S3V2)

Description:
Patient Transfusion Risk Group 3, sufficient information for donor selection, no special and informational messages

Patient name: Demo Patient
Medical facility patient ID = PDS3V2TRG3, source patient ID = 9

Patient demographics: gender: male, age: 60, race: white

Patient medical conditions: BD (Blood hematological disorders)
ICD10 reported D56.1 (THA - Thalassemia)

Patient phenotype: B, D+C+c-E-e+Cw-K-k+, Fy(a+b+), Jk(a+b+), M+N+S+s+ (choice depth = 17, blood type: B+)

IHF determines Transfusion Condition using ICD10 reported (D56.1).
Transfusion Condition is THA - Thalassemia.

IHF assigned Transfusion Risk Group: 3
TRG is assigned based on THA transfusion condition
TRG choice depth recommendation = 13 (ABO, D, C, c, E, e, K, Fy^a, Fy^b, Jk^a, Jk^b, S, s)

IHF Messages:
Sufficient phenotype information available for donor selection
Special and informational messages: N/A

Comments: N/A

Scenario: #11 (S4V1)

Description:
Patient Transfusion Risk Group 4, sufficient information for donor selection, no special and informational messages

Patient name: Demo Patient
Medical facility patient ID = PDS4V1TRG4, source patient ID = 11
Patient demographics: gender: male, age: 52, race: white

Patient medical conditions: N/A

Patient phenotype: AB, D+C-c+E-e+K-k+ (choice depth = 8, blood type: AB+)
Unexpected specific antibodies- anti-E

IHF assigned Transfusion Risk Group: 4
TRG is assigned based on IMMU001 - Unexpected specific antibodies
TRG choice depth recommendation = 7+ (ABO, D, C, c, E, e, K) + specific antibodies

IHF Messages:
Sufficient phenotype information available for donor selection
Special and informational messages: N/A

Comments: N/A

Scenario: #12 (S4V2)

Description:
Patient Transfusion Risk Group 4, sufficient information for donor selection, no special and informational messages

Patient name: Demo Patient
Medical facility patient ID = PDS4V2TRG4, source patient ID = 12
Patient demographics: gender: female, age: 22, race: white

Patient medical conditions: N/A

Patient phenotype: A, D+C+c-E-e+K- (choice depth = 7, blood type: A+)

Unexpected specific antibodies- anti-A1

IHF assigned Transfusion Risk Group: 4
TRG is assigned based on Unexpected specific antibodies
TRG choice depth recommendation = 7 (ABO, D, C, c, E, e, K) + specific antibodies

IHF Messages:
Sufficient phenotype information available for donor selection
Special and informational messages: N/A

Comments:
Anti-A1 антитела у реципиентов с АВО-принадлежностью А и АВ трактуются в IHF как «Unexpected specific antibodies». Подбирают А-негативные эритроциты, совместимые по остальным антигенам.

Scenario: #13 (S4V3)

Description:
Patient Transfusion Risk Group 4, sufficient information for donor selection, no special and informational messages

Patient name: Demo Patient
Medical facility patient ID = PDS4V3TRG4, source patient ID = 13
Patient demographics: gender: male, age: 56, race: white

Patient medical conditions: N/A

Patient phenotype: AB, D+C+c+E-e+K-k+, Jk(a-b+) (choice depth = 10, blood type: AB+)

Unexpected specific antibodies- anti-Jk^a

IHF assigned Transfusion Risk Group: 4
TRG is assigned based on Unexpected specific antibodies
TRG choice depth recommendation = 7+ (ABO, D, C, c, E, e, K) + specific antibodies

IHF Messages:
Sufficient phenotype information available for donor selection
Special and informational messages: N/A

Comments: N/A

Scenario: #14 (S5V1)

Description:
Patient Transfusion Risk Group 5, sufficient information for donor selection, no special and informational messages

Patient name: Demo Patient
Medical facility patient ID = PDS5V1TRG5, source patient ID = 14
Patient demographics: gender: male, age: 38, race: white

Patient medical conditions:
ICD10 reported C92.1 (Chronic myeloid leukaemia [CML], BCR/ABL-positive)

Patient phenotype: A, D+C+c+E-e+Cw-K-k+, Fy(a+b+), Jk(a+b-), M-N+S+s+, Le(a-b+) (choice depth = 19, blood type: A+)
Unexpected specific antibodies- anti-Le^a

IHF determines Transfusion Condition using ICD10 reported (C92.1).
Transfusion Condition is CML - Chronic myeloid leukemia.

IHF assigned Transfusion Risk Group: 5
TRG is assigned based on CML, Unexpected specific antibodies transfusion condition
TRG choice depth recommendation = 13+ (ABO, D, C, c, E, e, K, Fy^a, Fy^b, Jk^a, Jk^b, S, s) + specific antibodies

IHF Messages:
Sufficient phenotype information available for donor selection
Special and informational messages: N/A

Comments: N/A

Scenario: #15 (S5V2)

Description:
Patient Transfusion Risk Group 5, sufficient information for donor selection, no special and informational messages

Patient name: Demo Patient
Medical facility patient ID = PDS5V2TRG5, source patient ID = 15
Patient demographics: gender: male, age: 65, race: white

Patient medical conditions:
ICD10 reported C92.2 (CML - Atypical chronic myeloid leukemia, BCR/ABL-negative)

Patient phenotype: AB, D+C+c+E-e+Cw-K-k+, Fy(a+b-), Jk(a-b+), M+N+S+s+ (choice depth = 17, blood type: AB+)
Unexpected specific antibodies- anti-Jk^a

IHF determines Transfusion Condition using ICD10 reported (C92.2).
Transfusion Condition is CML - Chronic myeloid leukemia.

IHF assigned Transfusion Risk Group: 5
TRG is assigned based on CML, Unexpected specific antibodies transfusion condition
TRG choice depth recommendation = 13+ (ABO, D, C, c, E, e, K, Fy^a, Fy^b, Jk^a, Jk^b, S, s) + specific antibodies

IHF Messages:
Sufficient phenotype information available for donor selection
Special and informational messages: N/A

Comments: N/A

Scenario: #16 (S5V3)

Description:
Patient Transfusion Risk Group 5, sufficient information for donor selection, no special and informational messages

Patient name: Demo Patient
Medical facility patient ID = PDS5V3TRG5, source patient ID = 16
Patient demographics: gender: female, age: 60, race: white

Patient medical conditions:
Patient medical conditions: BD (Blood hematological disorders)
ICD10 reported D46 (MDS - Myelodysplastic syndrome)

Patient phenotype: O, D-C-c+E-e+Cw-K-k+, Fy(a+b-), Jk(a+b-), M-N+S+s+ (choice depth = 17, blood type: O-)
Unexpected specific antibodies- M

IHF determines Transfusion Condition using ICD10 reported (D46).
Transfusion Condition is MDS - Myelodysplastic syndrome.

IHF assigned Transfusion Risk Group: 5
TRG is assigned based on CML, Unexpected specific antibodies transfusion condition
TRG choice depth recommendation = 13+ (ABO, D, C, c, E, e, K, Fy^a, Fy^b, Jk^a, Jk^b, S, s) + specific antibodies

IHF Messages:
Sufficient phenotype information available for donor selection
Special and informational messages: N/A

Comments: N/A

Scenario: #19 (S5V4)

Description:
Patient Transfusion Risk Group 5, sufficient information for donor selection, no special and informational messages

Patient name: Demo Patient
Medical facility patient ID = PDS5V4TRG5, source patient ID = 19
Patient demographics: gender: female, age: 60, race: white

Patient medical conditions: BD (Blood hematological disorders)
ICD10 reported D46 (MDS - Myelodysplastic syndrome)

Patient phenotype: O, D-C-c+E-e+Cw-K-k+, Fy(a+b+), Jk(a+b-), M+N+S-s+, Lu(a-b+) (choice depth = 19, blood type: O-)
Unexpected specific antibodies- anti-Lu^a

IHF determines Transfusion Condition using ICD10 reported (D46).
Transfusion Condition is MDS - Myelodysplastic syndrome.

IHF assigned Transfusion Risk Group: 5
TRG is assigned based on MD , Unexpected specific antibodies transfusion condition
TRG choice depth recommendation = 13+ (ABO, D, C, c, E, e, K, Fy^a, Fy^b, Jk^a, Jk^b, S, s) + specific antibodies

IHF Messages:
Sufficient phenotype information available for donor selection
Special and informational messages: N/A

Comments: N/A

Scenario: #22 (S5V5)

Description:
Patient Transfusion Risk Group 5, sufficient information for donor selection, no special and informational messages

Patient name: Demo Patient
Medical facility patient ID = PDS5V5TRG5, source patient ID = 22
Patient demographics: gender: female, age: 40, race: black

Patient medical conditions:
ICD10 reported C92.2 (CML - Atypical chronic myeloid leukemia, BCR/ABL-negative)

Patient phenotype: AB, D+C+c+E-e+Cw-K-k+, Fy(a+b-), Jk(a-b+), M+N+S+s+ (choice depth = 17, blood type: AB+)
Unexpected specific antibodies- anti-Jk^a

IHF determines Transfusion Condition using ICD10 reported (C92.2).
Transfusion Condition is CML - Chronic myeloid leukaemia.

IHF assigned Transfusion Risk Group: 5
TRG is assigned based on CML, Unexpected specific antibodies transfusion condition
TRG choice depth recommendation = 13+ (ABO, D, C, c, E, e, K, Fy^a, Fy^b, Jk^a, Jk^b, S, s) + specific antibodies

IHF Messages:
Sufficient phenotype information available for donor selection
Special and informational messages: N/A

Comments: N/A

Scenario: #21 (S2V5)

Description:
Patient Transfusion Risk Group 2, sufficient information for donor selection, no special and informational messages

Patient name: Demo Patient
Medical facility patient ID = PDS2V6TRG2, source patient ID = 21

Patient demographics: gender: male, age: 29, race: black

Patient medical conditions: BD (Blood hematological disorders)
Patient phenotype: B, D-C-c+E-e+K- (choice depth = 7, blood type: B-)

IHF assigned Transfusion Risk Group: 2
TRG is assigned based on BD transfusion condition
TRG choice depth recommendation = 7 (ABO, D, C, c, E, e, K)

IHF Messages:
Sufficient phenotype information available for donor selection
Special and informational messages: N/A

Comments: N/A

Scenario: #20 (S4V4)

Description:
Patient Transfusion Risk Group 4, sufficient information for donor selection, special and informational messages

Patient name: Demo Patient
Medical facility patient ID = PDS4V4TRG4, source patient ID = 22
Patient demographics: gender: male, age: 62, race: white

Patient medical conditions: BD (Blood hematological disorders)

Patient phenotype: B, D+C-c+E-e+K-k+ (choice depth = 8, blood type: AB+)
Unexpected specific antibodies- anti-e

IHF assigned Transfusion Risk Group: 4
TRG is assigned based on Unexpected specific antibodies
TRG choice depth recommendation = 7+ (ABO, D, C, c, E, e, K) + specific antibodies

IHF Messages:
Sufficient Information Available For Donor Selection.

Special and informational messages: Specific Autoantibody. Phenotype antigen and antibody are present. Antibody = Anti-e, Antigen = e.

Comments: N/A
Options:

- Possible manual RBC unit selection using IHF "model 2 (all available information)" patient to donor match.

Scenario: #17 (S6V1)

Description:
Patient Transfusion Risk Group 6, sufficient information for donor selection, no special and informational messages

Patient name: Demo Patient
Medical facility patient ID = PDS6V1TRG6, source patient ID = 17
Patient demographics: gender: female, age: 43, race: white

Patient medical conditions: BD (Blood hematological disorders)
ICD3 reported D59 (Diseases of the blood and blood-forming organs and certain disorders involving the immune mechanism)

Patient phenotype: B, D+C+c+E-e+Cw-K-k+, Fy(a+b+), Jk(a-b+), M-N+S-s+ (choice depth = 17, blood type: B+)
Multispecific/unidentified antibodies

IHF assigned Transfusion Risk Group: 6
TRG is assigned based on BD, Multispecific/unidentified *antibodies transfusion condition*
TRG choice depth recommendation = 14 (ABO, D, C, c, E, e, K, Fy^a, Fy^b, Jk^a, Jk^b, S, s, M)

IHF Messages:
Sufficient phenotype information available for donor selection
Special and informational messages: N/A

Comments: N/A

Scenario: #18 (S6V2)

Description:
Patient Transfusion Risk Group 6, sufficient information for donor selection, no special and informational messages

Patient name: Demo Patient
Medical facility patient ID = PDS6V2TRG6, source patient ID = 18
Patient demographics: gender: male, age: 62, race: white

Patient medical conditions: BD (Blood hematological disorders)
ICD3 reported C90 (Malignant neoplasms, stated or presumed to be primary, of lymphoid, haematopoietic and related tissue)

Patient phenotype: B, D-C-c+E-e+Cw-K-k+, Fy(a+b-), Jk(a+b+), M+N+S-s+ (choice depth = 17, blood type: B-)

IHF assigned Transfusion Risk Group: 6
TRG is assigned based on BD, Daratumumab transfusion condition
TRG choice depth recommendation = 14 (ABO, D, C, c, E, e, K, Fy^a, Fy^b, Jk^a, Jk^b, S, s, M)

IHF Messages:
Sufficient phenotype information available for donor selection
Special and informational messages: N/A

Comments: N/A

Donor Profiles

Donor demographic and laboratory information received
Phenotype Usage Type (PUT) determined
System messages generated

Scenario: #1 (SD0V1)

Description:
Phenotype Usage Type: Unique, no special and informational messages

Donor name: Demo Donor
Official Donor ID = DDSD0V1UNQ8, source donor ID = 1
Donor demographics: gender: female, age: 51, race: white

Donor phenotype: O, D-C-c+E-e+Cw-K-k+, Fy(a-b+), Jk(a-b+), M-N+S-s+
(Donor choice depth = 17, blood type: O-)

IHF assigned PUT: Unique
PUT is assigned based on Phenotype ID: UNQ8
UNQ8 Phenotype Usage Type Base = *16 O, D-C-c+E-e+K-k+, F(a+b-), Jk(a+b-), M-N+S-s+*
(O, D-C-c+E-e+K-k+Fy^{a}+Fy^{b}-Jk^{a}+Jk^{b}- M-N+S-s+)

System Messages: N/A

Comments:
Standard of Care recommendation: Cryopreserve RBC unit

Scenario: #2 (SD0V3)

Description:
Phenotype Usage Type: Unique, no special and informational messages

Donor name: Demo Donor
Official Donor ID = DDSD0V3UNQ51, source donor ID = 3
Donor demographics: gender: female, age: 58, race: white

Donor phenotype: O, D+C+c-E-e+Cw-K-k+, Fy(a-b+), Jk(a+b-), M+N-S+s- (Donor choice depth = 17, blood type: O+)

IHF assigned PUT: Unique
PUT is assigned based on Donor Phenotype ID: UNQ51
UNQ51 Phenotype Usage Type Base = 16 O, D+C+c-E-e+K-k+, Fy(a-b+), Jk(a+b-), M+N-S+s-
(O, D+C+c-E-e+K-k+Fy^{a}-Fy^{b}+Jk^{a}+Jk^{b}- M+N-S+s-)

System Messages: N/A

Comments:
Standard of Care recommendation: Cryopreserve RBC unit

Scenario: #3 (SD0V4)

Description:
Phenotype Usage Type: Unique, no special and informational messages

Donor name: Demo Donor
Official Donor ID = DDSD0V4UNQ80, source donor ID = 30
Donor demographics: gender: male, age: 26, race: black

Donor phenotype: O, D+C-c+E+e-Cw-K-k+, Fy(a-b-), Jk(a-b+), Kp(a-b+), Le(a+b-), Lu(a-b+), M-N+S-s+, P1+
(Donor choice depth = 24, blood type: O+)

IHF assigned PUT: Unique
PUT is assigned based on Donor Phenotype ID: UNQ80
UNQ80 Phenotype Usage Type Base = 16 O, D+C-c+E+e-K-k+, Fy(a-b-), Jk(a-b+), M-N+S-s+ *(O, D+C-c+E+e-K-k+Fy^{a}-Fy^{b}-Jk^{a}-Jk^{b}+ M-N+S-s+)*

System Messages: N/A

Comments:
Standard of Care recommendation: Cryopreserve RBC unit

Scenario: #4 (SD1V1)

Description:
Phenotype Usage Type: Extraordinary, no special and informational messages

Donor name: Demo Donor
Official Donor ID = DDSD1V1EXT1, source donor ID = 4
Donor demographics: gender: male, age: 21, race: white

Donor phenotype: O, D+C+c-E+e-Cw-K-k+ (Donor choice depth = 9, blood type: O+)

IHF assigned PUT: Extraordinary
PUT is assigned based on Donor Phenotype ID: EXT1
EXT1 Phenotype Usage Type Base = *8 O, D+C+c-E+e-K-k+ (O, D+C+c-E+e-K-k+)*

System Messages: N/A

Comments:
Standard of Care recommendation: Cryopreserve RBC unit

Scenario: #5 (SD1V2)

Description:
Phenotype Usage Type: Extraordinary, no special and informational messages

Donor name: Demo Donor
Official Donor ID = DDSD1V2EXT12, source donor ID = 23
Donor demographics: gender: female, age: 47, race: white

Donor phenotype: O, D-C-c+E-e+Cw-K-k+, Fy(a+b+), Jk(a+b-), Kp(a+b-), Le(a+b-), Lu(a-b+), M+N+S+s+, P1-
(Donor choice depth = 24, blood type: O-)

IHF assigned PUT: Extraordinary
PUT is assigned based on Donor Phenotype ID: EXT12
EXT12 Phenotype Usage Type Base = 10 O, D-C-c+E-e+K-k+, Kp(a+b-), (O, D-C-c+E-e-K-k+ Kp^a+Kp^b-)

System Messages: N/A

Comments:
Standard of Care recommendation: Cryopreserve RBC unit

Scenario: #6 (SD2V1)

Description:
Phenotype Usage Type: Universal, no special and informational messages

Donor name: Demo Donor
Official Donor ID = DDSD2V1UNV1, source donor ID = 5
Donor demographics: gender: male, age: 23, race: white

Donor phenotype: O, D-C-c+E-e+Cw-K-k+ (Donor choice depth = 9, blood type: O-)

IHF assigned PUT: Universal
PUT is assigned based on Donor Phenotype ID: UNV1
UNV1 Phenotype Usage Type Base = 8 O, D-C-c+E-e+K-k+, (O, D-C-c+E-e-K-k+)

System Messages: N/A

Comments:
Standard of Care recommendation: maintain a minimal supply level of refrigerated RBC units.

Scenario: #7 (SD2V2)

Description:
Phenotype Usage Type: Universal, no special and informational messages

Donor name: Demo Donor
Official Donor ID = DDSD2V2UNV11, source donor ID = 6
Donor demographics: gender: male, age: 26, race: white

Donor phenotype: O, D-C-c+E-e+Cw-K-k+, Fy(a+b+), Jk(a+b+), Kp(a-b+), Le(a-b-), Lu(a-b+), M+N+S+s+, P1-
(Donor choice depth = 24, blood type: O-)

IHF assigned PUT: Universal
PUT is assigned based on Donor Phenotype ID: UNV1
UNV1 Phenotype Usage Type Base = 8 O, D-C-c+E-e+K-k+, (O, D-C-c+E-e-K-k+)

System Messages: N/A

Comments:
Standard of Care recommendation: maintain a minimal supply level of refrigerated RBC units.

Scenario: #8 (SD2V6)

Description:
Phenotype Usage Type: Universal, no special and informational messages

Donor name: Demo Donor
Official Donor ID = DDSD2V6UNV1, source donor ID = 31
Donor demographics: gender: male, age: 23, race: black

Donor phenotype: O, D-C-c+E-e+Cw-K-k+, Fy(a-b+), Jk(a+b-), Kp(a-b+), Le(a-b+), Lu(a-b+), M-N+S+s+, P1+
(Donor choice depth = 24, blood type: O-)

IHF assigned PUT: Universal
PUT is assigned based on Donor Phenotype ID: UNV1
UNV1 Phenotype Usage Type Base = 8 O, D-C-c+E-e+K-k+, (O, D-C-c+E-e-K-k+)
System Messages: N/A

Comments:
Standard of Care recommendation: maintain a minimal supply level of refrigerated RBC units.

Scenario: #9 (SD3V1)

Description:
Phenotype Usage Type: Required, no special and informational messages

Donor name: Demo Donor
Official Donor ID = DDSD3V1REQ2, source donor ID = 7
Donor demographics: gender: male, age: 28, race: white

Donor phenotype: A, D+C+c-E-e+Cw-K-k+, Fy(a+b+), Jk(a+b+), Kp(a-b+), Le(a-b+), Lu(a-b+), M+N+S-s+, P1+ (Donor choice depth = 24, blood type: A+)

IHF assigned PUT: Required
PUT is assigned based on Donor Phenotype ID: REQ2
REQ2 Phenotype Usage Type Base = 8 A, D+C+c-E-e+K-k+, (A, D+C+c-E-e-K-k+)

System Messages: N/A

Comments:
Standard of Care recommendation: maintain a minimal supply level of refrigerated RBC units.

Scenario: #10 (SD3V3)

Description:
Phenotype Usage Type: Required, no special and informational messages

Donor name: Demo Donor
Official Donor ID = DDSD3V3REQ2, source donor ID = 9
Donor demographics: gender: female, age: 33, race: white

Donor phenotype: O, D+C+c-E-e+Cw-K-k+ (Donor choice depth = 9, blood type: O+)

IHF assigned PUT: Required
PUT is assigned based on Donor Phenotype ID: REQ1
REQ1 Phenotype Usage Type Base = 8 O, D+C+c-E-e+K-k+*, (O, D+C+c-E-e-K-k+)*

System Messages: N/A

Comments:
Standard of Care recommendation: maintain a minimal supply level of refrigerated RBC units.

Scenario: #11 (SD3V4)

Description:
Phenotype Usage Type: Required. Special and informational messages

Donor name: Demo Donor
Official Donor ID = DDSD3V4REQ2, source donor ID = 25
Donor demographics: gender: male, age: 28, race: white

Donor phenotype: A, D+C+c-E-e+Cw-K-k+, Fy(a+b+), Jk(a-b-), Kp(a-b+), Le(a-b+), Lu(a-b+), M+N+S-s+, P1+ (Donor choice depth = 24, blood type: A+)

IHF assigned PUT: Required
PUT is assigned based on Donor Phenotype ID: REQ2
REQ2 Phenotype Usage Type Base = 8 A, D+C+c-E-e+K-k+, (A, D+C+c-E-e-K-k+)

System Messages:
WARNING. Improbable or very rare antigen combination. Verify phenotype. phenotype = Jka-Jkb- frequency is RARE

Comments:
Perform donor Jk^a (Jk) and Jk^b(Jk) blood retyping and update donor blood typing information.

Scenario: #12 (SD4V1)

Description:
Phenotype Usage Type: Common, no special and informational messages

Donor name: Demo Donor
Official Donor ID = DDSD4V1COM, source donor ID = 11

Donor demographics: gender: female, age: 51, race: white

Donor phenotype: A, D+C-c+E+e+Cw-K-k+ (Donor choice depth = 9, blood type: A+)

IHF assigned PUT: Common
PUT is assigned based on Not applicable

System Messages: N/A

Comments: N/A

Scenario: #13 (SD4V3)

Description:
Phenotype Usage Type: Common, no special and informational messages

Donor name: Demo Donor
Official Donor ID = DDSD4V1COM, source donor ID = 13
Donor demographics: gender: male, age: 31, race: white

Donor phenotype: AB, D+C+c+E-e+Cw-K-k+, Fy(a-b+), Jk(a+b-), M+N+S+s- (Donor choice depth = 17, blood type: A+)

IHF assigned PUT: Common
PUT is assigned based on Not applicable

System Messages: N/A

Comments: N/A

Scenario: #14 (SD4V14)

Description:
Phenotype Usage Type: Common. Special and informational messages

Donor name: Demo Donor
Official Donor ID = DDSD4V14COM, source donor ID = 28
Donor demographics: gender: female, age: 22, race: white

Donor phenotype: AB, D-C+c+E-e+Cw-K-k+, Fy(a+b-), Jk(a-b+), Kp(a-b+), Le(a+b-), Lu(a-b-), M+N-S-s+, P1+ (Donor choice depth = 24, blood type: AB-)

IHF assigned PUT: Common
PUT is assigned based on Not applicable

System Messages:
WARNING. Improbable or very rare antigen combination. Verify phenotype. Phenotype = Lua-Lub- frequency is VERYRARE

Comments:
Perform donor Lu^a (Lu) and Lu^b(Lu) blood retyping and update donor blood typing information.

Blood Unit Profiles

Unit information received
System messages generated

Scenario: #1 (SD0V1)

Description:
Red Blood Cell. Cryopreservation. Phenotype Usage Type: Unique. Special and informational messages

Blood bank name: Demo Blood Bank
Unit ID = UD0001
Product: Red Blood Cell
Storage method: Cryopreservation
PUT: Unique
Unit start date: 05/30/2019 Unit end date: 05/30/2029

Unit phenotype: O, D-C-c+E-e+Cw-K-k+, Fy(a-b+), Jk(a-b+), M-N+S-s+
(Donor choice depth = 17, blood type: O-)

System Messages: Unit Storage Flag - green

Comments: RBC of Unique phenotype usage type should be cryopreserved

Scenario: #2 (SD0V3)

Description:
Red Blood Cell. Refrigerated. Phenotype Usage Type: Unique. Special and informational messages

Blood bank name: Demo Blood Bank
Unit ID = UD0003
Product: Red Blood Cell;
Storage method: Refrigerated;
PUT: Unique;
Unit start date: 02/11/21; Unit end date: 03/21/21

Unit phenotype: O, D+C+c-E-e+Cw-K-k+, Fy(a-b+), Jk(a+b-), M+N-S+s- (Donor choice depth = 17, blood type: O+)

System Messages: Unit Storage Flag - red

Comments: RBC of Unique phenotype usage type should be cryopreserved

Scenario: #3 (SD0V4)

Description:
Red Blood Cell. Cryopreservation. Phenotype Usage Type: Unique. Special and informational messages

Blood bank name: Demo Blood Bank
Unit ID = UD0030
Product: Red Blood Cell;
Storage method: Cryopreservation;
PUT: Unique;
Unit start date: 02/11/21; Unit end date: 02/11/31

Unit phenotype: O, D+C-c+E+e-Cw-K-k+, Fy(a-b-), Jk(a-b+), Kp(a-b+), Le(a+b-), Lu(a-b+), M-N+S-s+, P1+ (Donor choice depth = 24, blood type: O+)

System Messages: Unit Storage Flag - green

Comments: RBC of Unique phenotype usage type should be cryopreserved

Scenario: #4 (SD1V1)

Description:
Red Blood Cell. Cryopreservation. Phenotype Usage Type: Extraordinary. Special and informational messages

Blood bank name: Demo Blood Bank
Unit ID = UD0004
Product: Red Blood Cell;
Storage method: Cryopreservation;
PUT: Extraordinary;
Unit start date: 07/22/18; Unit end date: 07/22/28

Unit phenotype: O, D+C+c-E+e-Cw-K-k+ (Donor choice depth = 9, blood type: O+)

System Messages: Unit Storage Flag - green

Comments: RBC of Unique phenotype usage type should be cryopreserved

Scenario: #5 (SD1V2)

Description:
Red Blood Cell. Cryopreservation. Phenotype Usage Type: Extraordinary. Special and informational messages

Blood bank name: Demo Blood Bank
Unit ID = UD0023
Product: Red Blood Cell;
Storage method: Cryopreservation;
PUT: Extraordinary;
Unit start date: 07/22/18; Unit end date: 07/22/28

Unit phenotype: O, D-C-c+E-e+Cw-K-k+, Fy(a+b+), Jk(a+b-), Kp(a+b-), Le(a+b-), Lu(a-b+), M+N+S+s+, P1- (Donor choice depth = 24, blood type: O-)

System Messages: Unit Storage Flag - green

Comments: Эритроциты с Extraordinary фенотипом рекомендуется криоконсервировать.

Scenario: #6 (SD2V1)

Description:
Red Blood Cell. Refrigerated. Phenotype Usage Type: Universal. No special and informational messages

Blood bank name: Demo Blood Bank
Unit ID = UD0005
Product: Red Blood Cell;
Storage method: Refrigerated;
PUT: Universal;
Unit start date: 02/11/21; Unit end date: 03/21/21

Donor phenotype: O, D-C-c+E-e+Cw-K-k+ (Donor choice depth = 9, blood type: O-)

System Messages: N/A

Comments: N/A

Scenario: #7 (SD2V2)

Description:
Red Blood Cell. Refrigerated. Phenotype Usage Type: Universal. No special and informational messages

Blood bank name: Demo Blood Bank
Unit ID = UD0006
Product: Red Blood Cell;
Storage method: Refrigerated;
PUT: Universal;

Unit start date: 02/11/21; Unit end date: 03/21/21

Unit phenotype: O, D-C-c+E-e+Cw-K-k+, Fy(a+b+), Jk(a+b+), Kp(a-b+), Le(a-b-), Lu(a-b+), M+N+S+s+, P1- (Donor choice depth = 24, blood type: O-)

System Messages: N/A

Comments: N/A

Scenario: #8 (SD2V6)

Description:
Red Blood Cell. Refrigerated. Phenotype Usage Type: Universal. No special and informational messages

Blood bank name: Demo Blood Bank
Unit ID = UD0031
Product: Red Blood Cell;
Storage method: Refrigerated;
PUT: Universal;
Unit start date: 02/11/21; Unit end date: 03/21/21

Unit phenotype: O, D-C-c+E-e+Cw-K-k+, Fy(a-b+), Jk(a+b-), Kp(a-b+), Le(a-b+), Lu(a-b+), M-N+S+s+, P1+ (Donor choice depth = 24, blood type: O-)

System Messages: N/A

Comments: N/A

Scenario: #9 (SD3V1)

Description:
Red Blood Cell. Refrigerated. Phenotype Usage Type: Required. No special and informational messages

Blood bank name: Demo Blood Bank
Unit ID = UD0007
Product: Red Blood Cell;
Storage method: Refrigerated;
PUT: Required;
Unit start date: 02/11/21; Unit end date: 03/21/21

Unit phenotype: A, D+C+c-E-e+Cw-K-k+, Fy(a+b+), Jk(a+b+), Kp(a-b+), Le(a-b+), Lu(a-b+), M+N+S-s+, P1+ (Donor choice depth = 24, blood type: A+)

System Messages: N/A

Comments: N/A

Scenario: #10 (SD3V3)

Description:
Red Blood Cell. Refrigerated. Phenotype Usage Type: Required. No special and informational messages

Blood bank name: Demo Blood Bank
Unit ID = UD0009
Product: Red Blood Cell;
Storage method: Refrigerated;
PUT: Required;
Unit start date: 02/11/21; Unit end date: 03/21/21

Unit phenotype: O, D+C+c-E-e+Cw-K-k+ (Donor choice depth = 9, blood type: O+)

System Messages: N/A

Comments: N/A

Scenario: #11 (SD3V4)

Description:
Red Blood Cell. Refrigerated. Phenotype Usage Type: Required. No special and informational messages

Blood bank name: Demo Blood Bank
Unit ID = UD0025
Product: Red Blood Cell;
Storage method: Refrigerated;
PUT: Required;
Unit start date: 02/11/21; Unit end date: 03/21/21

Unit phenotype: A, D+C+c-E-e+Cw-K-k+, Fy(a+b+), Jk(a-b-), Kp(a-b+), Le(a-b+), Lu(a-b+), M+N+S-s+, P1+ (Donor choice depth = 24, blood type: A+)

System Messages: N/A

Comments: N/A

Scenario: #12 (SD4V1)

Description:
Red Blood Cell. Refrigerated. Phenotype Usage Type: Common. No special and informational messages

Blood bank name: Demo Blood Bank
Unit ID = UD0011
Product: Red Blood Cell;
Storage method: Refrigerated;
PUT: Common;
Unit start date: 02/11/21; Unit end date: 03/21/21

Unit phenotype: A, D+C-c+E+e+Cw-K-k+ (Donor choice depth = 9, blood type: A+)

System Messages: N/A

Comments: N/A

Scenario: #13 (SD4V3)

Description:
Red Blood Cell. Refrigerated. Phenotype Usage Type: Common. No special and informational messages

Blood bank name: Demo Blood Bank
Unit ID = UD0013
Product: Red Blood Cell;
Storage method: Refrigerated;
PUT: Common;
Unit start date: 02/11/21; Unit end date: 03/21/21

Unit phenotype: AB, D+C+c+E-e+Cw-K-k+, Fy(a-b+), Jk(a+b-), M+N+S+s- (Donor choice depth = 17, blood type: A+)

System Messages: N/A

Comments: N/A

Scenario: #14 (SD4V14)

Description:
Red Blood Cell. Cryopreservation. Phenotype Usage Type: Common. Special and informational messages

Blood bank name: Demo Blood Bank
Unit ID = UD0028
Product: Red Blood Cell;
Storage method: Cryopreservation;
PUT: Common;

Unit start date: 02/11/21; Unit end date: 03/21/21

Unit phenotype: AB, D-C+c+E-e+Cw-K-k+, Fy(a+b-), Jk(a-b+), Kp(a-b+), Le(a+b-), Lu(a-b-), M+N-S-s+, P1+ (Donor choice depth = 24, blood type: AB-)

System Messages: Unit Storage Flag - red

Comments: Эритроциты с Common фенотипом не рекомендуется криоконсервировать.

Donor-Patient Match

Scenario: #1 (S2V1)

Description:
Patient has MS0 (exact match), MS1(sufficient match) and MSM (mismatch) donors, no special and informational messages

Patient name: Demo Patient
Medical Facility Patient ID = PDS2V1TRG2, Source Patient ID = 1
Assigned Transfusion Risk Group: TRG 2
Patient phenotype: B, D-C-c+E-e+K- (choice depth = 7, blood type: B-).
Choice depth recommendation = 7 (ABO, D, C, c, E, e, K)

Final Compatibility

Exact Match
1.1 Source Donor ID = 15
Donor phenotype: B, D-C-c+E-e+Cw-K-k+ (choice depth = 9, blood type: B-).
MS0, Priority factor = 10200, Choice Factor = 0, Choice Depth = 7

Sufficient Match
1.2 Source Donor ID = 1
Donor phenotype: O, D-C-c+E-e+Cw-K-k+, Fy(a-b+), Jk(a-b+), M-N+S-s+
MS1, Priority factor = 11200, Choice Factor = 1, Choice Depth = 7

Source Donor ID = 5
Donor phenotype: O, D-C-c+E-e+Cw-K-k+.
MS1, Priority factor = 11200, Choice Factor = 1, Choice Depth = 7

Mismatch
1.3 Source Donor ID = 17
Donor phenotype: AB, D-C-c+E-e+Cw-K-k+
MSM, Priority factor = 15200, Choice Factor = -1, Choice Depth = 7

IHF Messages:
Special and informational messages: N/A

Comments: N/A

Scenario: #10 (S3V3)

Description:
The patient does not have Exact and Sufficient matched donors, special and informational messages

Patient name: Demo Patient
Medical facility patient ID = PDS3V3TRG3, Source Patient ID = 10
Assigned Transfusion Risk Group: TRG = 3
Patient phenotype: O, D+C-c+E+e+Cw-K-k+, Fy(a+b+), M+N+S+s+ (choice depth = 15, blood type: O+).
Choice depth recommendation = 13 (ABO, D, C, c, E, e, K, Fy^a, Fy^b, Jk^a, Jk^b, S, s)

Final Compatibility
Insufficient phenotype information available for donor selection

IHF Messages:
Special and informational messages: N/A

Comments:
Options:

- Possible manual RBC unit selection using IHF "model 2 (all available information)" patient to donor match.
- Perform patient Kidd (Jk) blood typing and update patient blood typing information.

Scenario: #2 (S2V2)

Description:
Patient has MS0 (exact match) and MSM (mismatch) donors, no special and informational messages

Patient name: Demo Patient
Medical facility patient ID = PDS2V2TRG2, source patient ID = 2
Assigned Transfusion Risk Group: TRG = 2
Patient phenotype: O, D-C-c+E-e+K- (choice depth = 7, blood type: O-).
Choice depth recommendation = 7 (ABO, D, C, c, E, e, K)

Final Compatibility

Exact match

Source Donor ID = 1
Donor phenotype: O, D-C-c+E-e+Cw-K-k+, Fy(a-b+), Jk(a-b+), M-N+S-s+
MS0, Priority factor = 10200, Choice Factor = 0, Choice Depth = 7

Sufficient match: N/A

Mismatch
Source Donor ID = 17
Donor phenotype: AB, D-C-c+E-e+Cw-K-k+
MSM, Priority factor = 15211, Choice Factor = -1, Choice Depth = 7

IHF Messages:
Special and informational messages: N/A

Comments: N/A

Scenario: #3 (S2V3)

Description:
Patient has MS1(sufficient match) and MSM (mismatch) donors, no special and informational messages

Patient name: Demo Patient
Medical facility patient ID = PDS2V3TRG2, Source Patient ID = 3
Assigned Transfusion Risk Group: TRG = 2
Patient phenotype: A, D(DP)C+c+E(DP)e+K- (choice depth = 7, blood type: O D(DP).
Choice depth recommendation = 7 (ABO, D, C, c, E, e, K)

Final Compatibility

Exact Match
N/A

Sufficient Match
Source Donor ID = 1
Donor phenotype: O, D-C-c+E-e+Cw-K-k+, Fy(a-b+), Jk(a-b+), M-N+S-s+
MS1, Priority factor = 11101, Choice Factor = 1, Choice Depth = 7

Source Donor ID = 5
Donor phenotype: O, D-C-c+E-e+Cw-K-k+.
MS1, Priority factor = 11101, Choice Factor = 1, Choice Depth = 7

Mismatch
Source Donor ID = 17
Donor phenotype: AB, D-C-c+E-e+Cw-K-k+
MSM, Priority factor = 15101, Choice Factor = -1, Choice Depth = 7

IHF Messages:
Special and informational messages: N/A

Comments: N/A

Scenario: #6 (S1V1)

Description:
Patient has MS0 (exact match), MS1(sufficient match) and MSM (mismatch) donors, no special and informational messages

Patient name: Demo Patient
Medical Facility Patient ID = PDS1V1TRG1, source patient ID = 6
Assigned Transfusion Risk Group: TRG 1
Patient phenotype: A, D-K- (choice depth = 3, blood type: A-).
Choice depth recommendation = 3 (ABO, D, K)

Final Compatibility

Exact Match
Source Donor ID = 16
Donor phenotype: O, D-C-c+E-e+Cw-K-k+, Fy(a+b+), Jk(a+b-), M+N+S+s+ (Donor choice depth = 17, blood type: O-).
MS0, Priority factor = 10200, Choice Factor = 0, Choice Depth = 3

Sufficient Match
Source Donor ID = 1
Donor phenotype: O, D-C-c+E-e+Cw-K-k+, Fy(a-b+), Jk(a-b+), M-N+S-s+
MS1, Priority factor = 11200, Choice Factor = 1, Choice Depth = 3

Source Donor ID = 5
Donor phenotype: O, D-C-c+E-e+Cw-K-k+.
MS1, Priority factor = 11200, Choice Factor = 1, Choice Depth = 3

Mismatch
Source Donor ID = 17
Donor phenotype: AB, D-C-c+E-e+Cw-K-k+
MSM, Priority factor = 15200, Choice Factor = -1, Choice Depth = 3

IHF Messages:
Special and informational messages: N/A

Comments: N/A

Scenario: #8 (S3V1)

Description:
Patient has MS1(sufficient match) and MSM (mismatch) donors, no special and informational messages

Patient name: Demo Patient
Medical Facility Patient ID = PDS3V1TRG3, source patient ID = 8
Assigned Transfusion Risk Group: TRG 3
Patient phenotype: B, D+C+c+E-e+K-k+, Fy(a+b+), Jk(a-b+), M-N+S-s+ (choice depth = 16, blood type: B+)
Choice depth recommendation = 13 (ABO, D, C, c, E, e, K, Fy^a, Fy^b, Jk^a, Jk^b, S, s)

Final Compatibility

Exact Match N/A

Sufficient Match

Source Donor ID = 1
Donor phenotype: O, D-C-c+E-e+Cw-K-k+, Fy(a-b+), Jk(a-b+), M-N+S-s+
MS1, Priority factor = 11212, Choice Factor = 4, Choice Depth = 13

Source Donor ID = 20
Donor phenotype: B, D-C+c+E-e+Cw-K-k+, Fy(a+b+), Jk(a-b+), M-N+S-s+
MS1, Priority factor = 11210, Choice Factor = 1, Choice Depth = 13

Mismatch
Source Donor ID = 17
Donor phenotype: AB, D-C-c+E-e+Cw-K-k+
MSM, Priority factor = 15211, Choice Factor = -1, Choice Depth = 7

IHF Messages:
Special and informational messages: N/A

Comments: N/A

Scenario: #13 (S4V3)

Description:
Patient has MS1(sufficient match) and MSM (mismatch) donors, no special and informational messages

Patient name: Demo Patient
Medical Facility Patient ID = PDS4V3TRG4, source patient ID = 13
Assigned Transfusion Risk Group: TRG 4
Patient phenotype: AB, D+C+c+E-e+K-k+, Jk(a-b+) (choice depth = 10, blood type: AB+). Unexpected specific antibodies- anti-Jk^a
Choice depth recommendation = 7+ (ABO, D, C, c, E, e, K) + specific antibodies

Final Compatibility

Exact Match N/A

Sufficient Match

Source Donor ID = 1
Donor phenotype: O, D-C-c+E-e+Cw-K-k+, Fy(a-b+), Jk(a-b+), M-N+S-s+

MS1, Priority factor = 13211, Choice Factor = 3, Choice Depth = 8

Source Donor ID = 21
Donor phenotype: AB, D-C+c+E-e+Cw-K-k+, Fy(a+b-), Jk(a-b+), Kp(a-b+), Le(a+b-), Lu(a-b+), M+N-S-s+, P1+
MS1, Priority factor = 10210, Choice Factor = 1, Choice Depth = 8

Mismatch
Source Donor ID = 6
Donor phenotype: O, D-C-c+E-e+Cw-K-k+, Fy(a+b+), Jk(a+b+), Kp(a-b+), Le(a-b-), Lu(a-b+), M+N+S+s+, P1-
MSM, Priority factor = 28211, Choice Factor = -1, Choice Depth = 8

IHF Messages:
Special and informational messages: N/A

Comments: N/A

Scenario: #12 (S4V2)

Description:
Patient has MS1(sufficient match) and MSM (mismatch) donors, no special and informational messages

Patient name: Demo Patient
Medical Facility Patient ID = PDS4V2TRG4, source patient ID = 12
Assigned Transfusion Risk Group: TRG 4
Patient phenotype: A, D+C+c-E-e+K- (choice depth = 7, blood type: A+).
Unexpected specific antibodies- anti-A1
Choice depth recommendation = 7+ (ABO, D, C, c, E, e, K) + specific antibodies

Final Compatibility

Exact Match N/A

Sufficient Match

Source Donor ID = 4
Donor phenotype: O, D-C+c-E-e+Cw-K-k+ (choice depth = 9, blood type: O-)
MS1, Priority factor = 11210, Choice Factor = 2, Choice Depth = 7

Source Donor ID = 3
Donor phenotype: O, D+C+c-E-e+Cw-K-k+ Fy(a-b+), Jk(a+b-), M+N-S+s- (choice depth = 17, blood type: O-)
MS1, Priority factor = 11200, Choice Factor = 1, Choice Depth = 7

Mismatch

Source Donor ID = 17
Donor phenotype: AB, D-C-c+E-e+Cw-K-k+
MSM, Priority factor = 15451, Choice Factor = -2, Choice Depth = 7

IHF Messages:
Special and informational messages: N/A

Comments: N/A

Scenario: #4 (S2V4)

Description:
The patient does not have Exact and Sufficient matched donors, special and informational messages

Patient name: Demo Patient
Medical facility patient ID = PDS2V4TRG2, source patient ID = 4
Assigned Transfusion Risk Group: TRG = 2
Patient phenotype: B, D+C+c+E-e-K-k+ (choice depth = 8, blood type: B+)
Choice depth recommendation = *7 (ABO, D, C, c, E, e, K)*

Final Compatibility
Insufficient phenotype information available for donor selection

IHF Messages:
Special and informational messages: N/A

Comments:
Options:

- Possible manual RBC unit selection using IHF “model 2 (all available information)” patient to donor match.
- Perform patient E(Rh) and e(Rh) blood retyping and update patient blood typing information.

Scenario: #16 (S5V3)

Description:
Patient has MS1(sufficient match) and MSM (mismatch) donors, no special and informational messages

Patient name: Demo Patient
Medical Facility Patient ID = PDS5V3TRG5, source patient ID = 16
Assigned Transfusion Risk Group: TRG 5

Patient phenotype: O, D-C-c+E-e+Cw-K-k+, Fy(a+b-), Jk(a+b-), M-N+S+s+ (choice depth = 17, blood type: O-). Unexpected specific antibodies- M
Choice depth recommendation *= 13+ (ABO, D, C, c, E, e, K, Fy^a, Fy^b, Jk^a, Jk^b, S, s) + specific antibodies*

Final Compatibility

Exact Match N/A

Sufficient Match

Source Donor ID = 10
Donor phenotype: O, D-C-c+E-e+Cw-K-k+, Fy(a+b+), Jk(a+b-), Kp(a-b+), Le(a-b+), Lu(a-b+), M+N+S+s+, P1+ (Donor choice depth = 24, blood type: O-). MS1, Priority factor = 11211, Choice Factor =3, Choice Depth = 14

Source Donor ID = 24
Donor phenotype: O, D-C-c+E-e+Cw-K-k+, Fy(a-b+), Jk(a+b-), Kp(a-b+), Le(a-b+), Lu(a-b+), M-N+S+s+, P1-
MS1, Priority factor = 11212, Choice Factor = 4, Choice Depth = 14

Mismatch
Source Donor ID = 6
Donor phenotype: O, D-C-c+E-e+Cw-K-k+, Fy(a+b+), Jk(a+b+), Kp(a-b+), Le(a-b-), Lu(a-b+), M+N+S+s+, P1-
MSM, Priority factor = 11371, Choice Factor = -1, Choice Depth = 14

IHF Messages:
Special and informational messages: N/A

Comments: N/A

Scenario: #17 (S6V1)

Description:
Patient has MS1(sufficient match) and MSM (mismatch) donors, no special and informational messages

Patient name: Demo Patient
Medical Facility Patient ID = ID = PDS6V1TRG6, source patient ID = 17
Assigned Transfusion Risk Group: TRG 6
Patient phenotype: B, D+C+c+E-e+Cw-K-k+, Fy(a+b+), Jk(a-b+), M-N+S-s+ (choice depth = 17, blood type: B+). Multispecific/unidentified antibodies

Choice depth recommendation *= 14 (ABO, D, C, c, E, e, K, Fy^a, Fy^b, Jk^a, Jk^b, S, s, M)*

Final Compatibility

Exact Match N/A

Sufficient Match
Source Donor ID = 1
Donor phenotype: O, D-C-c+E-e+Cw-K-k+, Fy(a-b+), Jk(a-b+), M-N+S-s+
MS1, Priority factor = 11212, Choice Factor =4, Choice Depth = 14

Source Donor ID = 20
Donor phenotype: B, D-C+c+E-e+Cw-K-k+, Fy(a+b+), Jk(a-b+), M-N+S-s+
MS1, Priority factor = 11210, Choice Factor = 1, Choice Depth = 14

Mismatch
Source Donor ID = 6
Donor phenotype: O, D-C-c+E-e+Cw-K-k+, Fy(a+b+), Jk(a+b+), Kp(a-b+), Le(a-b-), Lu(a-b+), M+N+S+s+, P1-
MSM, Priority factor = 11711, Choice Factor = -3, Choice Depth = 14

IHF Messages:
Special and informational messages: N/A

Comments: N/A

Scenario: #21 (S2V5)

Description:
Patient has MS0 (exact match), MS1(sufficient match) and MSM (mismatch) donors, no special and informational messages

Patient name: Demo Patient
Medical Facility Patient ID = PDS2V6TRG2, source patient ID = 21
Assigned Transfusion Risk Group: TRG 2
Patient phenotype: B, D-C-c+E-e+K- (choice depth = 7, blood type: B-)
Choice depth recommendation = 7 (ABO, D, C, c, E, e, K)

Final Compatibility

Exact Match
Source Donor ID = 29
Donor phenotype: B, D-C-c+E-e+Cw-K-k+ (choice depth = 9, blood type: B-).
MS0, Priority factor = 10200, Choice Factor = 0, Choice Depth = 7

Sufficient Match
Source Donor ID = 31
Donor phenotype: O, D-C-c+E-e+Cw-K-k+, Fy(a-b+), Jk(a+b-), Kp(a-b+), Le(a-b+), Lu(a-b+), M-N+S+s+, P1-
MS1, Priority factor = 11200, Choice Factor = 1, Choice Depth = 7

Mismatch

Source Donor ID = 17
Donor phenotype: AB, D-C-c+E-e+Cw-K-k+
MSM, Priority factor = 30200, Choice Factor = -2, Choice Depth = 7

IHF Messages:
Special and informational messages: N/A

Comments: N/A

Scenario: #22 (S5V5)

Description:
Patient has MS1(sufficient match) and MSM (mismatch) donors, no special and informational messages

Patient name: Demo Patient
Medical Facility ID = PDS5V5TRG5, source patient ID = 22
Assigned Transfusion Risk Group: TRG 5
Patient phenotype: AB, D+C+c+E-e+Cw-K-k+, Fy(a+b-), Jk(a-b+), M+N+S+s+ (choice depth = 17, blood type: AB+). Unexpected specific antibodies- anti-Jk^a

Choice depth recommendation *= 13+ (ABO, D, C, c, E, e, K, Fy^a, Fy^b, Jk^a, Jk^b, S, s)* + specific antibodies

Final Compatibility

Exact Match N/A

Sufficient Match

Source Donor ID = 32
Donor phenotype: B, D+C+c+E-e+Cw-K-k+, Fy(a-b+), Jk(a-b+), M+N+S+s+, P1+ (Donor choice depth = 24, blood type: O-)
MS1, Priority factor = 12212, Choice Factor =4, Choice Depth = 13

Mismatch
Source Donor ID = 24
Donor phenotype: O, D-C-c+E-e+Cw-K-k+, Fy(a-b+), Jk(a+b-), Kp(a-b+), Le(a-b+), Lu(a-b+), M-N+S+s+, P1-
MS1, Priority factor = 26363, Choice Factor = -2, Choice Depth = 13

IHF Messages:
Special and informational messages: N/A

Comments: N/A

Patient-Unit Selection Choice Ranking and System Recommendations

Scenario: #1 (S2V1)

Description:
Patient has MS0 (exact match), MS1(sufficient match) and MSM (mismatch) units, top unit selection ranking and top system recommendations are identical, no special and informational messages

Patient name: Demo Patient
Medical Facility Patient ID = PDS2V1TRG2, Source Patient ID = 1
Assigned Transfusion Risk Group: TRG 2
Patient phenotype: B, D-C-c+E-e+K- (choice depth = 7, blood type: B-).
Choice depth recommendation = 7 (ABO, D, C, c, E, e, K)

Patient – Unit selection ranking, top 3 units (unit selection rank = 1, 2, 3)

Unit	Unit Expiration Date	Phenotype Usage Type (PUT)	Storage Method	Unit Additional Factor	Donor Match Score	Donor Priority Factor	Unit Priority Factor	Unit Selection Rank
UD0015	03/21/2021	Common	Refrg	0	MS0	10200	10200	**1**
UD0018	03/21/2021	Universal	Refrg	0	MS1	11200	11200	2
UD0024	03/21/2021	Universal	Refrg	0	MS1	11200	11200	3

Patient – Unit top recommendations (unit recommendation = 1, 2, 3)

Unit	Unit Selection Rank	Unit Recommendation	Comment
UD0015	1	**1**	Recommended RBC unit
UD0018	2	2	Recommended RBC unit
UD0005	4	3	Recommended RBC unit
UD0024	3	5	Unit taken by Patient (TRG, scenario, source patient id)

IHF Messages:
Special and informational messages: N/A

Comments: N/A

Scenario: #10 (S3V3)

Description:
Patient does not have Exact and Sufficient matched units, special and informational messages

Patient name: Demo Patient
Medical facility patient ID = PDS3V3TRG3, Patient IHF Key = 41748
Assigned Transfusion Risk Group: TRG = 3
Patient phenotype: O, D+C-c+E+e+Cw-K-k+, Fy(a+b+), M+N+S+s+ (choice depth = 15, blood type: O+).
Choice depth recommendation = 13 (ABO, D, C, c, E, e, K, Fy^a, Fy^b, Jk^a, Jk^b, S, s)

Patient – Unit selection ranking: N/A
Patient – Unit top recommendations : N/A

IHF Messages:
No recommended units
No matching donors

Comments:
Options:

- Possible manual RBC unit selection using IHF "model 2 (all available information)" patient to donor match.
- Perform patient Kidd (Jk) blood typing and update patient blood typing information.

Scenario: #2 (S2V2)

Description:
Patient has MS0 (exact match), MS1(sufficient match) and MSM (mismatch) units, top unit selection ranking and top system recommendations are identical, no special and informational messages

Patient name: Demo Patient
Medical facility patient ID = PDS2V2TRG2, source patient ID = 2
Assigned Transfusion Risk Group: TRG = 2
Patient phenotype: O, D-C-c+E-e+K- (choice depth = 7, blood type: O-).
Choice depth recommendation = 7 (ABO, D, C, c, E, e, K)

Patient – Unit selection ranking, top 3 units (unit selection rank = 1, 2, 3)

Unit	Unit Expiration Date	Phenotype Usage Type (PUT)	Storage Method	Unit Additional Factor	Donor Match Score	Donor Priority Factor	Unit Priority Factor	Unit Selection Rank
UD0005	03/21/2021	Universal	Refrg	0	MS0	10200	10200	**1**
UD0006	03/21/2021	Universal	Refrg	0	MS0	10200	10200	2
UD0010	03/21/2021	Universal	Refrg	0	MS0	10200	10200	3

Patient – Unit top recommendations (unit recommendation = 1, 2, 3)

Unit	Unit Selection Rank	Unit Recommendation	Comment
UD0005	1	**1**	Recommended RBC unit
UD0018	4	2	Recommended RBC unit
UD0006	2	3	Recommended RBC unit
UD0010	3	-1	Unit taken by Patient (TRG=5, source patient id= PDS5V4TRG5)

IHF Messages:
Special and informational messages: N/A

Comments: N/A

Scenario: #3 (S2V3)

Description:
Patient has MS1(sufficient match) and MSM (mismatch) units, top unit selection ranking and top system recommendations are identical, no special and informational messages

Patient name: Demo Patient
Medical facility patient ID = PDS2V3TRG2, Source Patient ID = 3
Assigned Transfusion Risk Group: TRG = 2
Patient phenotype: A, D(DP)C+c+E(DP)e+K- (choice depth = 7, blood type: O D(DP).
Choice depth recommendation = 7 (ABO, D, C, c, E, e, K)

Patient – Unit selection ranking, top 3 units (unit selection rank = 1, 2, 3)

Unit	Unit Expiration Date	Phenotype Usage Type (PUT)	Storage Method	Unit Additional Factor	Donor Match Score	Donor Priority Factor	Unit Priority Factor	Unit Selection Rank
UD0016	03/21/2021	Common	Refrg	0	MS1	10101	10101	**1**
UD0018	03/21/2021	Universal	Refrg	0	MS1	11101	11101	2
UD0005	03/21/2021	Universal	Refrg	0	MS1	11101	11101	3

Patient – Unit top recommendations (unit recommendation = 1, 2, 3)

Unit	Unit Selection Rank	Unit Recommendation	Comment
UD0016	1	**1**	Recommended RBC unit
UD0018	2	2	Recommended RBC unit
UD0005	3	-1	Unit taken by Patient (TRG=2, source patient id= PDS2V2TRG2)
UD0024	6	3	Recommended RBC unit

IHF Messages:

Special and informational messages: N/A

Comments: N/A

Scenario: #6 (S1V1)

Description:

Patient has MS1(sufficient match) and MSM (mismatch) units, top unit selection ranking and top system recommendations are not identical, no special and informational messages

Patient name: Demo Patient
Medical Facility Patient ID = PDS1V1TRG1, source patient ID = 6
Assigned Transfusion Risk Group: TRG 1
Patient phenotype: A, D-K- (choice depth = 3, blood type: A-).
Choice depth recommendation = 3 (ABO, D, K)

Patient – Unit selection ranking, top 3 units (unit selection rank = 1, 2, 3)

Unit	Unit Expiration Date	Phenotype Usage Type (PUT)	Storage Method	Unit Additional Factor	Donor Match Score	Donor Priority Factor	Unit Priority Factor	Unit Selection Rank
UD0016	03/21/2021	Common	Refrg	0	MS0	10200	10200	**1**
UD0018	03/21/2021	Universal	Refrg	0	MS1	11200	11200	2
UD0005	03/21/2021	Universal	Refrg	0	MS1	11200	11200	3

Patient – Unit top recommendations (unit recommendation = 1, 2, 3)

Unit	Unit Selection Rank	Unit Recommendation	Comment
UD0016	1	**-1**	Unit taken by Patient (TRG=2, Scenario: #3 (S2V3), source patient ID = PDS2V3TRG2)
UD0018	2	1	Recommended RBC unit
UD0005	3	-1	Unit taken by Patient (TRG=2, Scenario: #2 (S2V2), source patient ID = PDS2V2TRG2)
UD0006	4	2	Recommended RBC unit
UD0024	6	3	Recommended RBC unit

IHF Messages:
Special and informational messages: N/A

Comments: N/A

Scenario: #8 (S3V1)

Description:

Patient has MS1(sufficient match) and MSM (mismatch) units, top unit selection ranking and top system recommendations are not identical, no special and informational messages

Patient name: Demo Patient
Medical Facility Patient ID = PDS3V1TRG3, source patient ID = 8
Assigned Transfusion Risk Group: TRG 3
Patient phenotype: B, D+C+c+E-e+K-k+, Fy(a+b+), Jk(a-b+), M-N+S-s+ (choice depth = 16, blood type: B+)
Choice depth recommendation = 13 (ABO, D, C, c, E, e, K, Fy^a, Fy^b, Jk^a, Jk^b, S, s)

Patient – Unit selection ranking, top 3 units (unit selection rank = 1, 2, 3)

Unit	Unit Expiration Date	Phenotype Usage Type (PUT)	Storage Method	Unit Additional Factor	Donor Match Score	Donor Priority Factor	Unit Priority Factor	Unit Selection Rank
UD0020	03/21/2021	Common	Refrg	0	MS1	10210	10210	**1**
UD0001	05/30/2029	Unique	Cryo	0	MS1	11512	11512	2
UD0002	04/23/2028	Unique	Cryo	0	MS1	11512	11512	3

Patient – Unit top recommendations (unit recommendation = 1, 2, 3)

Unit	Unit Selection Rank	Unit Recommendation	Comment
UD0020	1	**-1**	Unit taken by Patient (TRG=6, source patient ID = PDS6V1TRG6)
UD0001	2	1	Recommended RBC unit
UD0002	3	-1	Unit taken by Patient (TRG=6, source patient ID = PDS6V2TRG6)

IHF Messages:
Special and informational messages: N/A

Comments: N/A

Scenario: #13 (S4V3)

Description:
Patient has MS1(sufficient match) and MSM (mismatch) units, top unit selection ranking and top system recommendations are identical, no special and informational messages

Patient name: Demo Patient
Medical Facility Patient ID = PDS4V3TRG4, source patient ID = 13
Assigned Transfusion Risk Group: TRG 4

Patient phenotype: AB, D+C+c+E-e+K-k+, Jk(a-b+) (choice depth = 10, blood type: AB+). Unexpected specific antibodies- anti-Jk[a]
Choice depth recommendation = 7+ (ABO, D, C, c, E, e, K) + specific antibodies

Patient – Unit selection ranking, top 3 units (unit selection rank = 1, 2, 3)

Unit	Unit Expiration Date	Phenotype Usage Type (PUT)	Storage Method	Unit Additional Factor	Donor Match Score	Donor Priority Factor	Unit Priority Factor	Unit Selection Rank
UD0021	03/21/2021	Common	Refrg	0	MS1	10210	10210	**1**
UD0022	03/21/2021	Common	Refrg	0	MS1	10210	10210	2
UD0028	04/23/2028	Common	Cryo	0	MS1	10510	10510	3

Patient – Unit top recommendations (unit recommendation = 1, 2, 3)

Unit	Unit Selection Rank	Unit Recommendation	Comment
UD0021	1	**1**	Recommended RBC unit
UD0022	2	-1	Unit taken by Patient (TRG=5, source patient ID = PDS5V2TRG5)
UD0028	3	2	Recommended RBC unit
UD0001	6	3	Recommended RBC unit

IHF Messages:
Special and informational messages: N/A

Comments: N/A

Scenario: #12 (S4V2)

Description:
Patient has MS1(sufficient match) and MSM (mismatch) units, top unit selection ranking and top system recommendations are not identical, no special and informational messages

Patient name: Demo Patient
Medical Facility Patient ID = PDS4V2TRG4, source patient ID = 12
Assigned Transfusion Risk Group: TRG 4
Patient phenotype: A, D+C+c-E-e+K- (choice depth = 7, blood type: A+).
Unexpected specific antibodies- anti-A1
Choice depth recommendation = 7+ (ABO, D, C, c, E, e, K) + specific antibodies

Patient – Unit selection ranking, top 3 units (unit selection rank = 1, 2, 3)

Unit	Unit Expiration Date	Phenotype Usage Type (PUT)	Storage Method	Unit Additional Factor	Donor Match Score	Donor Priority Factor	Unit Priority Factor	Unit Selection Rank
UD0003	03/21/2021	Unique	Refrg	0	MS1	11200	11200	**1**
UD0008	03/21/2021	Required	Refrg	0	MS1	11200	11200	2
UD0009	03/21/2021	Required	Refrg	0	MS1	11200	11200	3

Patient – Unit top recommendations (unit recommendation = 1, 2, 3)

Unit	Unit Selection Rank	Unit Recommendation	Comment
UD0003	1	**2**	Recommended RBC unit
UD0008	2	3	Recommended RBC unit
UD0009	3	1	Recommended RBC unit

IHF Messages:
Special and informational messages: N/A

Comments: N/A

Scenario: #4 (S2V4)

Description:
The patient does not have Exact and Sufficient matched units, special and informational messages

Patient name: Demo Patient
Medical facility patient ID = PDS2V4TRG2, source patient ID = 4
Assigned Transfusion Risk Group: TRG = 2
Patient phenotype: B, D+C+c+E-e-K-k+ (choice depth = 8, blood type: B+)
Choice depth recommendation = *7 (ABO, D, C, c, E, e, K)*

Patient – Unit selection ranking: N/A
Patient – Unit top recommendations: N/A

IHF Messages:
No recommended units
No matching donors

Comments:
Options:

1. Possible manual RBC unit selection using IHF “model 2 (all available information)” patient to donor match.
2. Perform patient E(Rh) and e(Rh) blood retyping and update patient blood typing information.

Scenario: #16 (S5V3)

Description:
Patient has MS1(sufficient match) and MSM (mismatch) units, top unit selection ranking and top system recommendations are identical, no special and informational messages

Patient name: Demo Patient
Medical Facility Patient ID = PDS5V3TRG5, source patient ID = 16
Assigned Transfusion Risk Group: TRG 5
Patient phenotype: O, D-C-c+E-e+Cw-K-k+, Fy(a+b-), Jk(a+b-), M-N+S+s+ (choice depth = 17, blood type: O-). Unexpected specific antibodies- M
Choice depth recommendation = *13+ (ABO, D, C, c, E, e, K, Fy^a, Fy^b, Jk^a, Jk^b, S, s) + specific antibodies*

Patient – Unit selection ranking, top 3 units (unit selection rank = 1, 2, 3)

Unit	Unit Expiration Date	Phenotype Usage Type (PUT)	Storage Method	Unit Additional Factor	Donor Match Score	Donor Priority Factor	Unit Priority Factor	Unit Selection Rank
UD0025	03/21/2021	Required	Refrg	0	MS1	10203	10203	**1**
UD0010	03/21/2021	Universal	Refrg	0	MS1	11211	11211	2
UD0024	03/21/2021	Universal	Refrg	0	MS1	11211	11211	3

Patient – Unit top recommendations (unit recommendation = 1, 2, 3)

Unit	Unit Selection Rank	Unit Recommendation	Comment
UD0025	1	**1**	Recommended RBC unit
UD0010	2	2	Recommended RBC unit
UD0024	3	3	Recommended RBC unit

IHF Messages:
Special and informational messages: N/A

Comments: N/A

Scenario: #17 (S6V1)

Description:
Patient has MS1(sufficient match) and MSM (mismatch) units, top unit selection ranking and top system recommendations are identical, no special and informational messages

Patient name: Demo Patient
Medical Facility Patient ID = ID = PDS6V1TRG6, source patient ID = 17
Assigned Transfusion Risk Group: TRG 6
Patient phenotype: B, D+C+c+E-e+Cw-K-k+, Fy(a+b+), Jk(a-b+), M-N+S-s+ (choice depth = 17, blood type: B+). Multispecific/unidentified antibodies

Choice depth recommendation *= 14 (ABO, D, C, c, E, e, K, Fy^a, Fy^b, Jk^a, Jk^b, S, s, M)*

Patient – Unit selection ranking, top 3 units (unit selection rank = 1, 2, 3)

Unit	Unit Expiration Date	Phenotype Usage Type (PUT)	Storage Method	Unit Additional Factor	Donor Match Score	Donor Priority Factor	Unit Priority Factor	Unit Selection Rank
UD0020	03/21/2021	Common	Refrg	0	MS1	10210	10210	**1**
UD0001	05/30/2029	Common	Cryo	0	MS1	11512	10210	2

Patient – Unit top recommendations (unit recommendation = 1, 2, 3)

Unit	Unit Selection Rank	Unit Recommendation	Comment
UD0020	1	**1**	Recommended RBC unit
UD0001	2	2	Recommended RBC unit

IHF Messages:
Special and informational messages: N/A

Comments: N/A

Scenario: #21 (S2V5)

Description:
Patient has MS0 (exact match), MS1(sufficient match) and MSM (mismatch) units, top unit selection ranking and top system recommendations are identical, no special and informational messages

Patient name: Demo Patient
Medical Facility Patient ID = PDS2V6TRG2, source patient ID = 21
Assigned Transfusion Risk Group: TRG 2
Patient phenotype: B, D-C-c+E-e+K- (choice depth = 7, blood type: B-)
Choice depth recommendation = 7 (ABO, D, C, c, E, e, K)

Patient – Unit selection ranking, top 3 units (unit selection rank = 1, 2, 3)

Unit	Unit Expiration Date	Phenotype Usage Type	Storage Method	Unit Additional Factor	Donor Match Score	Donor Priority Factor	Unit Priority Factor	Unit Selection Rank

		(PUT)						
UD0029	03/21/2021	Common	Refrg	0	MS0	10210	10200	**1**
UD0031	03/21/2021	Universal	Refrg	0	MS1	11512	11200	2

Patient – Unit top recommendations (unit recommendation = 1, 2, 3)

Unit	Unit Selection Rank	Unit Recommendation	Comment
UD0020	1	**1**	Recommended RBC unit
UD0001	2	2	Recommended RBC unit

IHF Messages:
Special and informational messages: N/A

Comments: N/A

Scenario: #22 (S5V5)

Description:
Patient has MS1(sufficient match) and MSM (mismatch) units, top unit selection ranking and top system recommendations are identical, no special and informational messages

Patient name: Demo Patient
Medical Facility ID = PDS5V5TRG5, source patient ID = 22
Assigned Transfusion Risk Group: TRG 5
Patient phenotype: AB, D+C+c+E-e+Cw-K-k+, Fy(a+b-), Jk(a-b+), M+N+S+s+ (choice depth = 17, blood type: AB+). Unexpected specific antibodies- anti-Jk^a
Choice depth recommendation = *13+ (ABO, D, C, c, E, e, K, Fy^a, Fy^b, Jk^a, Jk^b, S, s) + specific antibodies*

Patient – Unit selection ranking, top 3 units (unit selection rank = 1, 2, 3)

Unit	Unit Expiration Date	Phenotype Usage Type (PUT)	Storage Method	Unit Additional Factor	Donor Match Score	Donor Priority Factor	Unit Priority Factor	Unit Selection Rank
UD0032	03/21/2021	Common	Refrg	0	MS1	12212	10200	**1**

Patient – Unit top recommendations (unit recommendation = 1, 2, 3)

Unit	Unit Selection Rank	Unit Recommendation	Comment
UD0032	1	**1**	Recommended RBC unit

IHF Messages:
Special and informational messages: N/A

Comments: N/A
