# Supplementary material for: Quantifying red blood cell compatibility beyond ABO and RhD: a recipient-centered model for matching, allocation, and inventory curation
Source: Front Med (Lausanne). 2026 Jul 14;13:1875496. doi: 10.3389/fmed.2026.1875496 (PMC13407175; doi:10.3389/fmed.2026.1875496)
Supplement: Supplementary file 3 [file Data_sheet_2.pdf]

Supplement B. Antigen Significance Factor (ASF)

This supplement provides the ASF scoring table and an illustrative example.

Table B1 Antigen Significance Factor (ASF): numeric score, expressed in arbitrary units assigned to each RBC antigen to represent its relative immunogenicity and clinical risk.

Blood Group System	Antigen	Antigen Significance Factor (ASF)
Rh	D	400
	C	200
	c	240
	E	240
	e	100
	C^w	40
	V	100
Kell	K	360
	k	2
	Js^a	20
	Js^b	100
	Kp^a	4
	Kp^b	2
Kidd	Jk^a	240
	Jk^b	220
	Jk3	100
Duffy	Fy^a	220
	Fy^b	50
	Fy3	20
MNS	M	160
	N	20
	S	50
	s	20
	U	100

Lewis	Le^a	20
	Le^b	10
Lutheran	Lu^a	20
	Lu^b	2
P1PK	P1	2
Wright	Wr^a	100
Colton	Co^b	100
Dombrock	Do^a	20

Worked example: daratumumab recipients and Table B2

Daratumumab causes nonspecific RBC agglutination, complicating antibody screening and crossmatch interpretation. In such patients often desirable to match Duffy (Fy^a, Fy^b), Kidd (Jk^a, Jk^b), and MNS (M, N, S, s) antigens. If ABO/Rh/Kell-matched units are available, compute the summed ASF for each candidate unit's incompatible antigens and select the unit with the lowest summed ASF to minimize alloimmunization risk and potential hemolytic complications.

Table B2 illustrates a recipient versus two candidate donors. For each matching donor antigen, a match score is 0. If the donor's phenotype carries an antigen absent in the recipient, that antigen is scored according to its Antigen Significance Factor (ASF). In this example, the summed ASF for donor 1 (one) is 240, and for donor 2 (two) is 120. Donor 2 (two) is recommended for transfusion because it has a lower total ASF.

Table B2 Comparison of recipient and donor phenotypes for the Duffy, Kidd, and MNS systems using the Antigen Significance Factor (ASF)

Antigens	Jk^a	Jk^b	Fy^a	Fy^b	M	N	S	s
Phenotype								
Patient	−	+	+	−	+	−	−	+
Donor 1	+	+	+	−	−	+	−	−
Donor 2	−	+	+	+	+	+	+	+
Comparison of Recipient and Donor Phenotypes using the Antigen Significance Factor (ASF)								
Donor 1	240	0	0	0	0	0	0	0
Donor 2	0	0	0	50	0	20	50	0
