# Supplementary material for: Quantifying red blood cell compatibility beyond ABO and RhD: a recipient-centered model for matching, allocation, and inventory curation
Source: Front Med (Lausanne). 2026 Jul 14;13:1875496. doi: 10.3389/fmed.2026.1875496 (PMC13407175; doi:10.3389/fmed.2026.1875496)
Supplement: Supplementary file 4 [file Data_Sheet_3.pdf]

Supplement C. Match Rules

This supplement provides rules governing donor–recipient matching.

Contents

Supplement C. Match Rules 1
- ABO — Match Rule 2
- RhD and K (Kell) — Match Rules 4
- All Antigens Except ABO, D, and K — Match Rules 5
- Antibody Category Rule 6
- Table C7 Antigen Match Rules 8
- Table C8 Antibody Match Rules 9

These rules define deterministic donor–recipient phenotype matching used in the WPCS algorithm. Match rules are grouped into two categories: Antigen and Antibody.

Table C1 Antigen category rules: match outcome evaluation — deterministic criteria and decision reasons.

Recipient Antigen	Donor Antigen	Match Result	MS Code	Degree of Matching
+	+	Compatible	MS0	Exact Match
-	-	Compatible	MS0	Exact Match
+	-	Compatible	MS1	Sufficient Match
-	+	Incompatible	MSM	Mismatch
+	Null	Unknown	Null	Unknown
-	Null	Unknown	Null	Unknown

NOTE:

- Symbols: + = antigen present; − = antigen absent.
- Unknown outcome: Occurs when the donor's typing lacks one or more antigen results that are present for the recipient.

Antigen match rules have different weights (Table C3). Antigens ABO, D (RhD), and K (Kell) have distinct clinical and operational priorities.

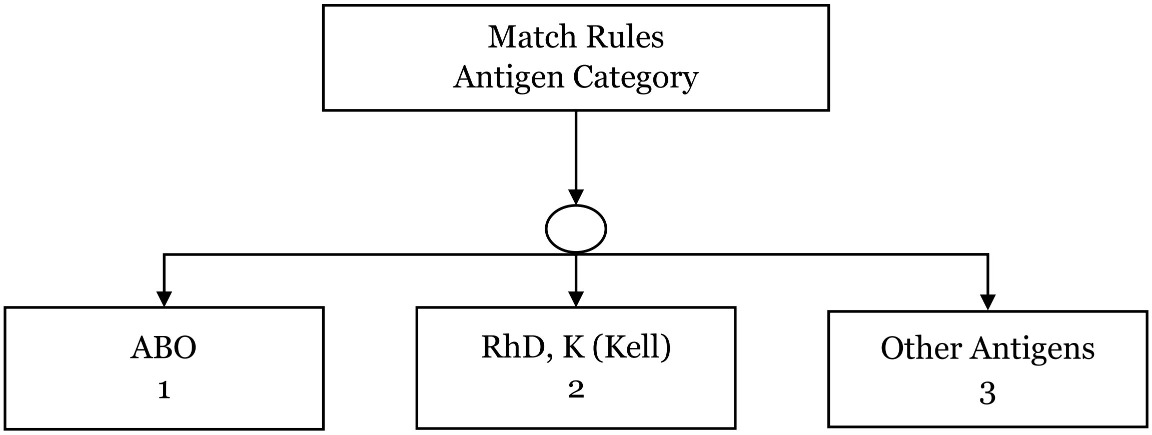

Figure C1 Phenotype Category Match Rules: ABO, D (RhD), and K (Kell) priority antigens. NOTE: Antigen match rules carry different weights for WPCS scoring. ABO, D (RhD), and K (Kell) are designated priority antigens and are handled with distinct clinical and operational logic in the matching algorithm.

ABO — Match Rule

Four (4) scenarios for selecting red blood cell (RBC) units based on the ABO blood type are summarized in Table C2. The table presents transfusion compatibility scenarios

between the recipient and the donor, each corresponding to a match status code (MS0, MS1, MSM) and a priority factor used in the WPCS matching algorithm.

Table C2 ABO blood group system: RBC selection scenarios.

Recipient ABO Blood Type	Donor ABO Blood Type			
	1st choice	2nd choice	3rd choice	4th choice
AB	AB	A	B	O
A	A	O	-	-
B	B	O	-	-
O	O	-	-	-

NOTE: "–" indicates incompatibility between donor and recipient RBCs. Transfusion of incompatible RBCs may cause severe hemolytic reactions.

Compatibility Scenarios in the ABO System

1) Identical ABO types
   - Selection: 1st choice
   - Match Status: Exact Match (MS0)
   - Priority Factor: 10000
   - Example: Recipient A, Donor A

2) Compatible but non-identical ABO types
   - Selection: 2nd, 3rd, or 4th choice
   - Match Status: Sufficient Match (MS1)
   - Priority Factors:
     - 2nd choice: 11000
     - 3rd choice: 12000
     - 4th choice: 13000

3) Incompatible ABO types
   - Selection: Excluded from consideration
   - Match Status: Mismatch (MSM)
   - Priority Factor: M

CLINICAL NOTE:
Transfusion of ABO-incompatible RBCs can result in severe and potentially fatal hemolytic transfusion reactions due to the presence of naturally occurring antibodies in the recipient.

WPCS NOTE:

ABO compatibility is a critical gating criterion in the WPCS phenotype-matching algorithm and is enforced before evaluating the extended phenotype match tier.

Table C3 summarizes ABO-based RBC selection tiers, linking compatibility levels to MS codes, priority factors, and representative donor–recipient pairs.

Table C3 ABO blood group system: compatibility, MS Code, Priority Factor, and examples.

ABO Selection Choice	Degree of Compatibility	MS Code	Priority Factor	Example Donor -> Recipient
1st choice	Exact Match	MS0	10000	A -> A
2nd choice	Sufficient Match	MS1	11000	O -> A
3rd choice	Sufficient Match	MS1	12000	B -> AB
4th choice	Sufficient Match	MS1	13000	O -> AB
Incompatible	Mismatch	MSM	M	A -> O

RhD and K (Kell) — Match Rules

Scenarios for matching RBC units for the D and K antigens are summarized in Table C4. The table presents transfusion compatibility options between the recipient and the donor, each corresponding to a match status code (MS0, MS1, MSM) and a priority factor used in the WPCS matching algorithm.

Compatibility Scenarios for Antigens D and K

1) Identical Antigen Presence
   - Selection: 1st choice
   - Match Status: Exact Match (MS0)
   - Priority Factor: 100
   - Example: Recipient D-, Donor D-

2) Phenotypically compatible
   - Selection: 2nd choice
   - Match Status: Sufficient Match (MS1)
   - Priority Factors: 110
   - Example: Recipient D+, Donor D-

3) Antigen incompatibility
   - Selection: Excluded from consideration
   - Match Status: Mismatch (MSM)

- Priority Factor: Defined by Antigen Significance Factor (ASF)
  - PF(RhD) = 380
  - PF(K) = 360

Table C4 D and K antigens: compatibility, MS Code, Priority Factor, and examples.

Antigen		Compatibility	Degree of Compatibility	MS Code	Priority Factor
Recipient	Donor				
+	+	Compatible	Exact Match	MS0	100
-	-	Compatible	Exact Match	MS0	100
+	-	Compatible	Sufficient Match	MS1	110
-	+	Incompatible	Mismatch	MSM	ASF (e.g., 380 or 360)

LEGEND: Exact Match (MS0) — PF = 100; Sufficient Match (MS1) — PF = 110; Mismatch (MSM) — Priority Factor (PF) equals Antigen Significance Factor (ASF); PF(D) = ASF(D), PF(K) = ASF(K)

NOTE: "–" indicates antigen absence. Antigens D and K are highly immunogenic; Transfusion of incompatible RBCs may cause severe hemolytic reactions.

All Antigens Except ABO, D, and K — Match Rules

Phenotype category rules for all antigens (excluding ABO, D, and K) establish compatibility tiers and priority factors for donor-recipient phenotype matching.

Possible donor–recipient compatibility scenarios for all antigens excluding ABO, RhD, and K (Kell)

1) Identical Antigen Presence
   - Selection: 1st choice
   - Match Status: Exact Match (MS0)
   - Priority Factor: 0
   - Example: Recipient C+, Donor C+

2) Phenotypically compatible
   - Selection: 2nd choice
   - Match Status: Sufficient Match (MS1)
   - Priority Factors: 1

- Example: Recipient C+, Donor C-

3) Antigen incompatibility
   - Selection: Excluded from consideration
   - Match Status: Mismatch (MSM)
   - Priority Factor: Defined by Antigen Significance Factor (ASF)
     - PF(Antigen) = ASF(Antigen)

Table C5 All antigens except ABO, D, and K: compatibility, MS Code, Priority Factor, and examples.

Antigen		Compatibility	Degree of Compatibility	MS Code	Priority Factor
Recipient	Donor				
+	+	Compatible	Exact Match	MS0	0
-	-	Compatible	Exact Match	MS0	0
+	-	Compatible	Sufficient Match	MS1	1
-	+	Incompatible	Mismatch	MSM	ASF

LEGEND: Exact Match (MS0) — PF = 0; Sufficient Match (MS1) — PF = 1; Mismatch (MSM) — Priority Factor (PF) equals Antigen Significance Factor (ASF); PF(C) = ASF(C), PF(M) = ASF(M)

NOTE: For all antigens except those of the ABO system, the Mismatch (MSM) Priority Factor (PF) equals the Antigen Significance Factor (ASF), determined by antigen immunogenicity and the clinical significance of the corresponding antibodies.

Antibody Category Rule

The antibody rule applies when the recipient has specific antibodies. If the patient currently has (or has ever been found to have) antibodies of a defined specificity, the corresponding antigen must be absent from the donor's phenotype.
Only two outcomes of recipient–donor compatibility are possible when alloantibodies are identified in the recipient: Exact Match (MS0) and Mismatch (MSM).

1. Exact Match (MS0)
   - Definition: The donor does not have the antigen against which the recipient's antibodies have been identified.
   - Priority Factor: 0
   - Example: Recipient C+/c- anti-c detected, донор: C+/c-

2. Mismatch (MSM)
    - Definition: The donor has the antigen against which the recipient's antibodies have been identified.
    - Priority Factor: Determined by the Antigen Significance Factor (ASF):
        - Antigens with ASF value ≥ 4 have a Priority Factor = 15,000
        - Antigens with ASF value < 4 have a Priority Factor = 10,000
    - Example: Recipient C+/c- with anti-c detected, донор: C-/c+

Table C6 Specific Antibody Priority Factor: Mismatch (MSM).

Antibody Specificity	Priority Factor
D, C, c, E, e, C^w, K, Js^b, Kp^a, Jk^a, Jk^b, Fy^a, Fy^b, M, S, s, U, Le^a, Le^b, Lu^a, Lu^b, P1	15000
k, Js^a, Kp^b, N	10000

LEGEND: Exact Match (MS0) — PF = 0; Mismatch (MSM) — ASF ≥ 4 → PF = 15,000; ASF < 4 → PF = 10,000.

NOTE: This rule strictly excludes donor units that carry antigens targeted by recipient alloantibodies, reflecting both the immunogenicity of the antigens and the clinical significance of the corresponding antibodies.

Table C7 Antigen Match Rules

Table C7 Antigen Match Rules

Match Rule	Match Rule Category	System	Antigen	Priority Factor		
				MS0	MS1	MSM
Rh, Antigen D	Antigen	Rh	D	100	110	400
Rh, Antigen C	Antigen	Rh	C	0	1	200
Rh, Antigen c	Antigen	Rh	c	0	1	240
Rh, Antigen E	Antigen	Rh	E	0	1	240
Rh, Antigen e	Antigen	Rh	e	0	1	100
Rh, Antigen Cw	Antigen	Rh	C^w	0	1	40
Rh, Antigen V	Antigen	Rh	V	0	1	100
Kell, Antigen K	Antigen	Kell	K	100	110	360
Kell, Antigen k	Antigen	Kell	k	0	1	2
Kell, Antigen Jsa	Antigen	Kell	Js^a	0	1	20
Kell, Antigen Jsb	Antigen	Kell	Js^b	0	1	100
Kell, Antigen Kpa	Antigen	Kell	Kp^a	0	1	4
Kell, Antigen Kpb	Antigen	Kell	Kp^b	0	1	2
Kidd, Antigen Jka	Antigen	Kidd	Jk^a	0	1	240
Kidd, Antigen Jkb	Antigen	Kidd	Jk^b	0	1	180
Kidd, Antigen Jk3	Antigen	Kidd	Jk3	0	1	100
Duffy, Antigen Fya	Antigen	Duffy	Fy^a	0	1	220
Duffy, Antigen Fyb	Antigen	Duffy	Fy^b	0	1	50
Duffy, Antigen Fy3	Antigen	Duffy	Fy3	0	1	20
MNS, Antigen M	Antigen	MNS	M	0	1	160
MNS, Antigen N	Antigen	MNS	N	0	1	20
MNS, Antigen S	Antigen	MNS	S	0	1	50
MNS, Antigen s	Antigen	MNS	s	0	1	20
MNS, Antigen U	Antigen	MNS	U	0	1	100
Lewis, Antigen Lea	Antigen	Lewis	Le^a	0	1	20
Lewis, Antigen Leb	Antigen	Lewis	Le^b	0	1	10
Lutheran, Antigen Lua	Antigen	Lutheran	Lu^a	0	1	20
Lutheran, Antigen Lub	Antigen	Lutheran	Lu^b	0	1	2
P1PK, Antigen P1	Antigen	P1PK	P_1	0	1	2
Wright, Wr^a	Antigen	Wright	Wr^a	0	1	100
Colton, Co^b	Antigen	Colton	Co^b	0	1	100
Dombrock, Do^a	Antigen	Dombrock	Do^a	0	1	20

Table C8 Antibody Match Rules

Table C8 Antibody Match Rules

Match Rule	Match Category	Antibody	Priority Factor		
			MS0	MS1	MSM
Antibody, D	Antibody	Anti-D	0		15000
Antibody, C	Antibody	Anti-C	0		15000
Antibody, c	Antibody	Anti-c	0		15000
Antibody, E	Antibody	Anti-E	0		15000
Antibody, e	Antibody	Anti-e	0		15000
Antibody, Cw	Antibody	Anti-C^w	0		15000
Antibody, V	Antibody	Anti-V	0		15000
Antibody, K	Antibody	Anti-K	0		15000
Antibody, k	Antibody	Anti-k	0		10000
Antibody, Jsa	Antibody	Anti-Js^a	0		10000
Antibody, Jsb	Antibody	Anti-Js^b	0		15000
Antibody, Kpa	Antibody	Anti-Kp^a	0		15000
Antibody, Kpb	Antibody	Anti-Kp^b	0		10000
Antibody, Jka	Antibody	Anti-Jk^a	0		15000
Antibody, Jkb	Antibody	Anti-Jk^b	0		15000
Antibody, Fya	Antibody	Anti-Fy^a	0		15000
Antibody, Fyb	Antibody	Anti-Fy^b	0		15000
Antibody, M	Antibody	Anti-M	0		15000
Antibody, N	Antibody	Anti-N	0		10000
Antibody, S	Antibody	Anti-S	0		15000
Antibody, s	Antibody	Anti-s	0		15000
Antibody, U	Antibody	Anti-U	0		15000
Antibody, Lea	Antibody	Anti-Le^a	0		15000
Antibody, Leb	Antibody	Anti-Le^b	0		15000
Antibody, Lua	Antibody	Anti-Lu^a	0		15000
Antibody, Lub	Antibody	Anti-Lu^b	0		10000
Antibody, P1	Antibody	Anti-P1	0		15000
