# Supplementary material for: Quantifying red blood cell compatibility beyond ABO and RhD: a recipient-centered model for matching, allocation, and inventory curation
Source: Front Med (Lausanne). 2026 Jul 14;13:1875496. doi: 10.3389/fmed.2026.1875496 (PMC13407175; doi:10.3389/fmed.2026.1875496)
Supplement: Supplementary file 5 [file Data_Sheet_4.pdf]

Supplement D. Phenotype Usage Type (PUT)

This supplement provides the complete PUT classification.

Contents

Supplement D. Phenotype Usage Type (PUT) 1

Universal Category 2

Required Category 2

Unique Category 3

Extraordinary Category 5

Common Category 7

Universal Category

Definition
The phenotype is defined by the absence of A, B, D, C, c, E, and K antigens and is therefore limited to the ABO, Rh (D, C, c, E, e), and Kell (K) systems.

ABO	A	B
	-	-

Rh	D	C	c	E	e
	-	-	+	-	+

Kell	K	k
	-	+

Figure D1 PUT. Antigen combinations defining the Universal category.

Usage
RBC units with this phenotype are intended for transfusion to recipients classified as TRG 0, 1, 2, and 4, and for urgent transfusions when the recipient's blood type is unknown.

Recommended standard of care
Maintain an unreduced stock of refrigerated RBC units to ensure immediate availability for routine and emergency needs.

NOTE: The Universal phenotype serves as the baseline when forming a donor registry for extended blood-group systems (Duffy, Kidd, MNS, and others).

Required Category

Definition
Contains the ABO, Rh (D, C, c, E, e), and Kell (K) antigens. RBCs express only one antigen in the pairs C/c and E/e. The K (Kell) antigen is absent in the phenotypes. Blood group AB is excluded from the Required category.

ABO	A	B
	-	-
	+	-
	-	+

Kell	K	k
	-	+

Rh	D	C	c	E	e
	-	-	+	-	+
	+	+	-	-	+
	+	-	+	-	+
	+	-	+	+	-

Figure D2 PUT. Antigen combinations defining the Required category.

Classification Rule — A phenotype is classified as Required if it exhibits one of the antigen combinations shown in this figure for each extended blood group system.

Usage

Phenotypes are in high demand for both planned and emergency transfusions. This level of compatibility is necessary for patients in TRG 2 and, in some cases, TRG 4.

Recommended standard of care

Maintain an unreduced stock of refrigerated RBC units.

NOTE 1: The Required phenotype serves as the baseline when forming a donor registry for extended blood-group systems (e.g., Duffy, Kidd, MNS).

NOTE 2: The antigen combination A-B-K-D-C-c+E-e+ meets the criteria for the Required classification but is assigned to a distinct category: Universal.

Unique Category

Definition

This category encompasses antigens from the ABO, Rh (D, C, c, E, e), Kell (K, k), Kidd (Jk^a, Jk^b), Duffy (Fy^a, Fy^b), and MNS (M, N, S, s) blood group systems. Phenotypes express only one antigen from each of the following antigen pairs: C/c, E/e, Fy^a/Fy^b, Jk^a/Jk^b, S/s, or M/N. Additionally, RBCs lack both antigens A and B of the ABO system, as well as K of the Kell system. The category also includes phenotypes in which either Fy^a or Fy^b antigens of the Duffy system are absent.

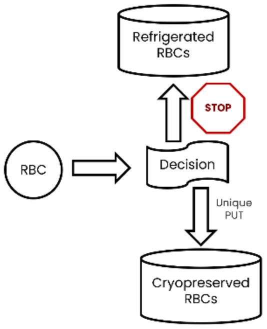

Unique PUT: cryopreservation.

ABO	A	B
	-	-

Kell	K	k
	-	+

Rh	D	C	c	E	e
	-	-	+	-	+
	+	+	-	-	+
	+	-	+	-	+
	+	-	+	+	-

Duffy	Fy^a	Fy^b
	+	-
	-	+
	-	-

Kidd	Jk^a	Jk^b
	+	-
	-	+

MNS	M	N
	+	-
	-	+
	S	s
	+	-
	-	+

Figure D3 PUT. Antigen combinations defining the Unique category.
Classification Rule — A phenotype is classified as Unique if it exhibits one of the antigen combinations shown in this figure for each extended blood group system.

Usage

RBCs with this level of compatibility are transfused to recipients in TRGs 3, 4, 5, and 6. The antigen combinations are compatible with genotypes that contain the relevant antigen-encoding genes in either homozygous or heterozygous states. Unique phenotypes are found in only a small number of donors but offer broad compatibility: they match more than 99% of recipients across 17 antigens — A, B, D, C, c, E, e, K, k, Jk^a, Jk^b, Fy^a, Fy^b, M, N, S, s.

Recommended standard of care

Candidates for cryopreservation, pending the application of phenotype-usage criteria currently being developed within the PUT framework.

Extraordinary Category

Definition

Phenotypes in this category lack an antigen or an antigen combination that is typically present in more than 99% of the population. Classification requires analysis of specific antigens and antigen combinations within the donor phenotype, as outlined below:

- Extraordinary 1 – a rare combination of Rh group antigens (Figure C4.1)
- Extraordinary 2 – lacking k antigen (Kell) (Figure C4.2)
- Extraordinary 3 – rare combination of Js(a+b-) antigens (Kell) (Figure C4.3)
- Extraordinary 4 – lacking the U antigen (MNS) (Figure C4.4)

NOTE: Phenotypes of this category have an optimal combination of ABO and Rh antigens.

Usage

RBCs of this category are used for transfusion only in rare cases, as they are incompatible with the phenotypes of the vast majority of recipients. However, when a recipient has a matching antigen profile and/or antibodies against an antigen widely prevalent in the population, these RBCs may be critically necessary.

Recommended standard of care

Candidates for cryopreservation, pending the application of phenotype-usage criteria currently being developed within the PUT framework.

CLASSIFICATION RULE:

A phenotype is classified as Extraordinary if it exhibits one of the antigen combinations shown in Figures 4.1 – 4.4 for each relevant blood group system.

Extraordinary 1

ABO	A	B
	-	-

Kell	K	k
	-	+

Rh	D	C	c	E	e
	-	+	-	-	+
	-	-	+	+	-

Figure D4.1 PUT. Antigen combinations defining the Extraordinary 1 category

Extraordinary 2

ABO	A	B
	-	-

Kell	K	k
	+	-

Rh	D	C	c	E	e
	-	-	+	-	+
	+	+	-	-	+
	+	-	+	-	+
	+	-	+	+	-

Figure D4.2 PUT. Antigen combinations defining the Extraordinary 2 category

Extraordinary 3

ABO	A	B
	-	-

Kell	K	k
	-	+
	Js^a	Js^b
	+	-

Rh	D	C	c	E	e
	-	-	+	-	+
	+	+	-	-	+
	+	-	+	-	+
	+	-	+	+	-

Figure D4.3 PUT. Antigen combinations defining the Extraordinary 3 category

Extraordinary 4

ABO	A	B
	-	-

Kell	K	k
	-	+

Rh	D	C	c	E	e
	-	-	+	-	+
	+	+	-	-	+
	+	-	+	-	+
	+	-	+	+	-

MNS	U
	-

Figure D4.4 PUT. Antigen combinations defining the Extraordinary 4 category

Common Category

Definition

When a phenotype does not belong to any of the above categories, it is classified as Common.

Recommended standard of care

It is not recommended to stockpile RBCs of this category or send them for long-term retention.
