# Supplementary material for: Quantifying red blood cell compatibility beyond ABO and RhD: a recipient-centered model for matching, allocation, and inventory curation
Source: Front Med (Lausanne). 2026 Jul 14;13:1875496. doi: 10.3389/fmed.2026.1875496 (PMC13407175; doi:10.3389/fmed.2026.1875496)

Supplement E. IHF Conceptual Data Model

This supplement provides the IHF framework conceptual data model.

The data model is where all IHF concepts, entities, and metadata come together, supporting the design of operational and analytical components, as well as data exchange specifications.

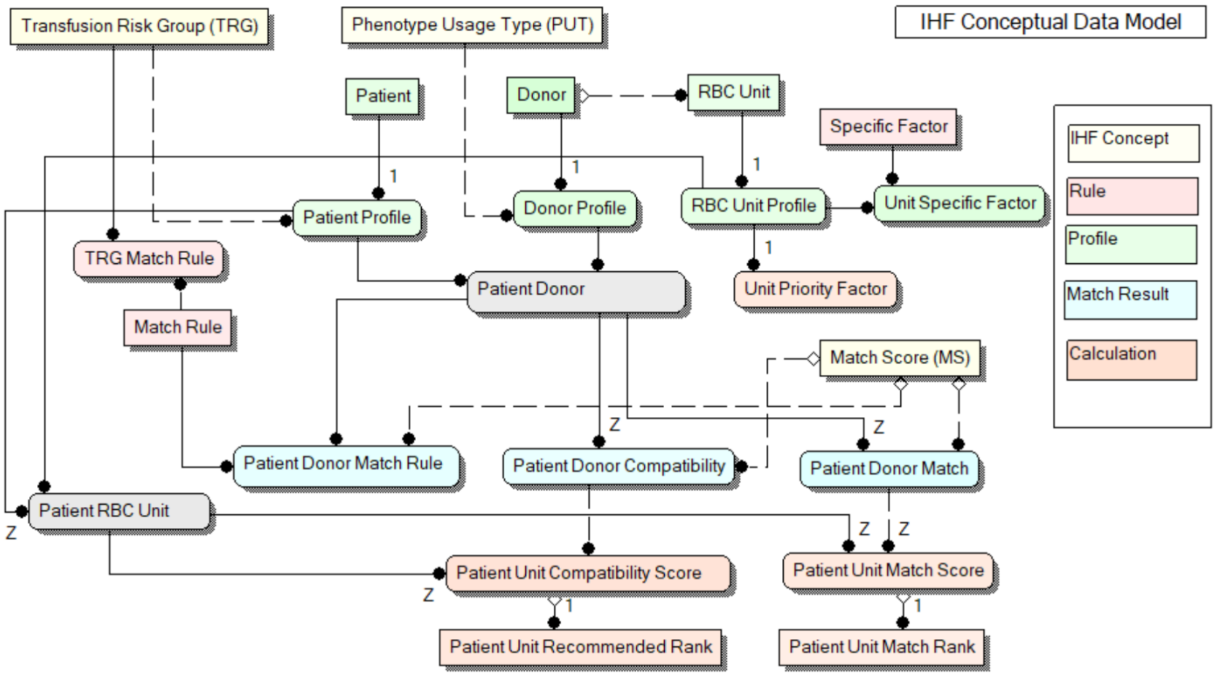

Supplement: Supplementary file 6 [file Data_sheet_5.pdf]
