# Supplementary material for: Quantifying red blood cell compatibility beyond ABO and RhD: a recipient-centered model for matching, allocation, and inventory curation
Source: Front Med (Lausanne). 2026 Jul 14;13:1875496. doi: 10.3389/fmed.2026.1875496 (PMC13407175; doi:10.3389/fmed.2026.1875496)

Supplement F. IHF Data Mart – Dimensional Model

This supplement provides the high-level dimensional data model.

The IHF framework analytical component database is implemented as a star-schema data mart.

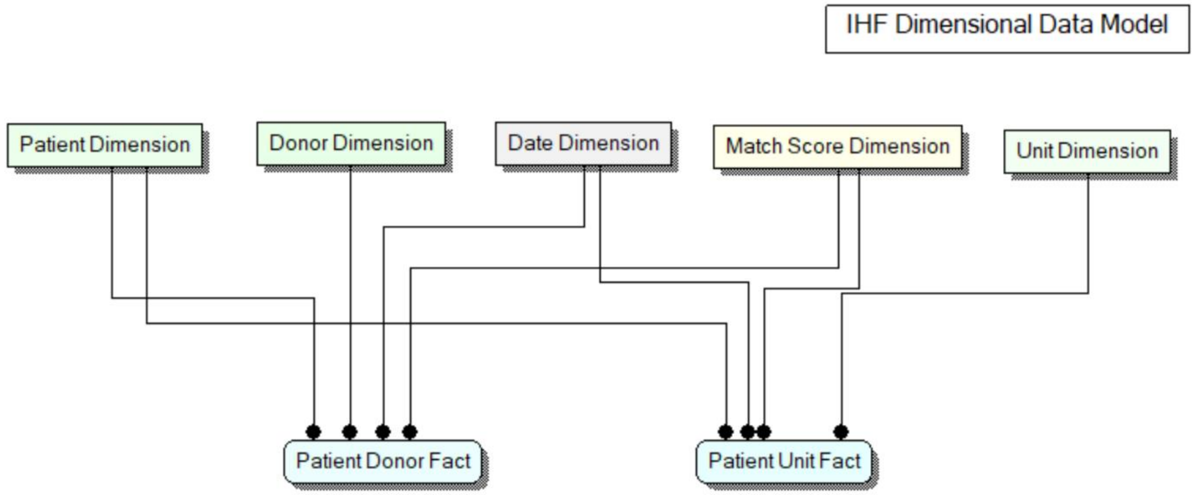

Supplement: Supplementary file 7 [file Data_sheet_6.pdf]
