# Supplementary material for: Quantifying red blood cell compatibility beyond ABO and RhD: a recipient-centered model for matching, allocation, and inventory curation
Source: Front Med (Lausanne). 2026 Jul 14;13:1875496. doi: 10.3389/fmed.2026.1875496 (PMC13407175; doi:10.3389/fmed.2026.1875496)
Supplement: Supplementary file 8 [file Data_sheet_7.pdf]

Supplement G. Worked Examples

This supplement provides comprehensive worked examples illustrating compatibility assessment, degree-of-phenotype-match evaluation, and selection and allocation logic.

Contents

Supplement G. Worked Examples 1

Worked example 1. Recipient – Donor compatibility: Match Score Code 2

Worked example 2. Matching donor and recipient phenotypes (TRG2) 3

Worked Example 3. Recipient-Unit Ranking 5

Worked Example 4. Recipient (TRG5) with Anti-M antibodies 6

Worked Example 5. Recipient (TRG2) and six donors 8

Worked Example 6. Double red cell population (DP), Summary and Interpretation 10

Worked Example 7. Double red-cell population (DP), Recipient (TRG 2) — Donor B1 vs Donor C1 11

Worked Example 8. Specific autoantibodies 13

Worked Example 1. Recipient – Donor compatibility: Match Score Code

The following tables illustrate how antigen-level compatibility between a recipient and donor determines the Match Score Code assigned by WPCS.

Table G1.1 Recipient–donor phenotypes compatible (identical antigen set); Match Score Code = MS0 (Exact Match).

MS0 — Exact Match								
		Rh					Kell	
	ABO	D	C	c	E	e	K	k
Recipient	A	-	-	+	+	-	-	+
Donor	A	-	-	+	+	-	-	+

Table G1.2 Recipient–donor phenotypes compatible; Match Score Code = MS1 (Sufficient Match).

MS1 — Sufficient Match								
		Rh					Kell	
	ABO	D	C	c	E	e	K	k
Recipient	A	-	-	+	+	-	-	+
Donor	A	-	-	+	-	-	-	+

Table G1.3 Recipient–donor phenotypes incompatible; Match Score Code = MSM (Mismatch).

MSM — Mismatch								
		Rh					Kell	
	ABO	D	C	c	E	e	K	k
Recipient	A	-	-	+	+	-	-	+
Donor	A	-	-	+	+	+	-	+

Worked Example 2. Matching donor and recipient phenotypes (TRG2)

Table G2.1 Recipient TRG2 — Inputs for the worked example: demographics, clinical baseline, and required antigens for phenotype matching.

Category	Parameter	Value
Classification	TRG	2
Demographics	Gender	Female
	Age	22
Clinical	Hematologic disorders	No
	History of repeated transfusions	No
Laboratory	Specific antibodies	None
Compatibility Requirements	Antigens matched	ABO, D, K, C, c, E, e

ABO	Rh					Kell	
ABO	D	C	c	E	e	K	k
B	+	-	+	+	-	-	+

NOTE: ABO is represented as a single composite antigen (A, B) for matching simplicity.

This example shows how the WPCS method prioritizes four candidate donors for the TRG2 recipient. Donors are ranked by compatibility using the categorical MS Code and the numeric Priority Factor (PF). Lower PF indicates a more desirable match.

Decision rule

Optimal donor is the one with the lowest combined MS category contribution and PF subtotal (MS Code governs category; PF resolves ordering within the same category).

Compact results table

Donor	MS Code	PF Subtotal	WPCS (displayed)	Rank	Notes
Donor 1	MS0	10200	MS0 10200	1	Compatible identical
Donor 2	MS1	10210	MS1 10210	2	Compatible
Donor 3	MS1	11210	MS1 11210	3	Compatible
Donor 4	MSM	See Chapter 11	MSM	Deprioritized	Incompatible

LEGEND: PF values used in this example: ABO (optimal, MS0) = 10,000; D = 100; K = 100. See Table 9.5 for per-antigen PF components and MS codes; see Appendix C for the complete antigen-

level matching rules. Lower PF indicates a more desirable match.

Conclusion

1. Donors 1–3 are compatible with recipient TRG2; (MS0, MS1). Donor 1 is the optimal choice, with the lowest PF (10200).
2. When MS Code is in the compatible category (MS0 or MS1), PF is the tie-breaking metric: lower PF is preferred. Donor 2 is preferred to Donor 3: PF (2) = 10210 < PF (3) = 11210.
3. Donor 4 is incompatible (MSM) and strongly deprioritized.

Table G2.2 Matching donor and recipient phenotypes using the WPCS method; MS Code, Priority Factor (PF), score, and rank.

	ABO	Rh					Kell		Rank	Choice Depth	Choice Factor
	ABO	D	C	c	E	e	K	k			
Recipient	B	+	-	+	+	-	-	+			
Donor 1	B	+	-	+	+	-	-	+	1		
MS Code	MS0	Ms0	MS0	MS0	MS0	MS0	Ms0		MS0	7	0
Priority Factor	10000	100	0	0	0	0	100		10200		
Donor 2	B	-	-	+	+	-	-	+	2		
MS Code	MS0	Ms1	MS0	MS0	MS0	MS0	Ms0		MS1	7	1
Priority Factor	10000	110	0	0	0	0	100		10210		
Donor 3	O	-	-	+	+	-	-	+	3		
MS Code	MS1	Ms1	MS0	MS0	MS0	MS0	Ms0		MS1	7	2
Priority Factor	11000	110	0	0	0	0	100		11210		
Donor 4	AB	+	+	-	+	-	-	+	N/A		
MS Code	MSM	MS0	MSM	MS1	MS0	MS0	MS0		MSM	7	-2
Priority Factor									See Chapter 11		

LEGEND: Priority Factor (PF) values used by the MS method: ABO (optimal, MS0) = 10,000; D = 100 (MS0); K = 100 (MS0). Lower PF indicates a more desirable match.

Worked Example 3. Recipient-Unit ranking

This example shows how the WPCS method ranks five RBC units for the TRG2 recipient. Units are ordered first by categorical MS Code and then by summed Priority Factor (PF), which includes unit-specific adjustments. A lower PF gives a higher rank.

Decision rule
Choose the unit with the lowest MS category contribution. Within the same MS category, select the unit with the lowest PF subtotal. Apply secondary criteria (expiry, inventory constraints) to break any remaining ties.

Method summary
PF subtotal = base PF assigned for MS category/compatibility + unit-specific adjustments (Additional PF). Lower PF = better. MS category governs primary ordering (e.g., MS0 > MS1 > MSM).

Table G3.1 Ranking RBC units using the WPCS method.

UNIT	Unit Expiration Date	Donor			RBC Unit			
		PUT Category	MS Code	PF Subtotal	Storage Method	Unit Specific Factor (PF)	PF Subtotal	RANK
1	15/04/2025	Common	MS0	10200	Refrigerated	0	10200	1
2	10/05/2025	Common	MS0	10200	Refrigerated	0	10200	1
3	11/04/2030	Common	MS1	10201	Refrigerated	0	10201	3
4	11/05/2030	Unique	MS0	10200	Frozen	300	10500	4
5	19/04/2025	Common	MSM	11160	Refrigerated	0	11160	N/A

LEGEND: MS0 = compatible, identical phenotype; MS1 = compatible; MSM = incompatible; PUT = Phenotype Usage Type; PF = Priority Factor (lower = higher priority); N/A = not applicable (incompatible).

Conclusion (final selection rationale)

1. Units 1, 2, and 4 are MS0 (base PF = 10200).
2. Unit 4 acquires an additional PF of 300 because it is a Unique PUT stored in a long-term freezer (PF subtotal = 10500), so it is deprioritized.
3. Units 1 and 2 remain the top choices (PF = 10200).
4. Comparing expiration dates, unit 1 (expiry 04-15-2025) is chosen as the optimal unit.

Worked Example 4. Recipient (TRG5) with Anti-M antibodies

Case summary

- Recipient (TRG5): phenotype A; D+; C+; c−; E−; e+; K−; k+; Jk(a+b+); Fy(a+b+); M−; N+; S+; s−. Antibody: anti-M.
- Donor: phenotype A; D+; C+; c−; E−; e+; K−; k+; Jk(a−b+); Fy(a−b+); M−; N+; S+; s−.
- Goal: evaluate recipient–donor compatibility and compute the degree of phenotype match using the WPCS method.

Decision rules (TRG5)

- TRG5 compatibility is assessed on 13 clinically relevant antigens plus any specific detected antibody; here, that yields choice depth = 14 (the 13 TRG5 antigens + M because of anti-M).
- For compatibility, the donor must be M− (to avoid the recipient's anti-M).
- The degree of phenotype match is calculated across the complete compared antigen set. In this example, both donor and recipient phenotypes include 16 antigens, so the choice depth for the degree of match is 16.

Input

Table G4.1 Recipient and Donor Phenotypes.

	Phenotype																Antibodies
	ABO	D	C	c	E	e	K	k	Jk^a	Jk^b	Fy^a	Fy^b	M	N	S	s	
Recipient	A	+	+	-	-	+	-	+	+	+	+	+	-	+	+	-	anti-M
Donor	A	+	+	-	-	+	-	+	-	+	-	+	-	+	+	-	

Key match rules and outcomes. Summary (see Table G4.2)

Table G4.2 Summary table. Recipient-donor compatibility and degree of phenotype match: match rules and outcomes.

N	Match Rule	MS Code	Priority Factor	Choice Factor	Choice Depth
1	ABO	MS0	10000	0	1
2	Antigen D	MS0	100	0	1
3	Antigen K	MS0	100	0	1
4	Antigen C	MS0	0	0	1
5	Antigen c	MS0	0	0	1
6	Antigen E	MS0	0	0	1
7	Antigen e	MS0	0	0	1
8	Antigen Jk^{a}	MS1	1	1	1
9	Antigen Jk^{b}	MS0	0	0	1
10	Antigen Fy^{a}	MS1	1	1	1
11	Antigen Fy^{b}	MS0	0	0	1
12	Antigen S	MS0	0	0	1
13	Antigen s	MS0	0	0	1
14	Anti-M (Antigen M)	MS0	0	0	1
Compatibility		MS1 (Sufficient Match)	10202	2	14
16	Antigen k	MS0	0	0	1
17	Antigen N	MS0	0	0	1
Match		MS1 (Sufficient Match)	10202	2	16

LEGEND: Compatibility assessed on TRG5 match rules (13 TRG5 antigens + M) → Choice Depth = 14—result: MS1 (Sufficient Match), PF=10202, Choice Factor=2. Degree of phenotype match evaluated across all compared antigens (16 antigens) → Choice Depth = 16—result: MS1 (Sufficient Match), PF=10202, Choice Factor=2.

Conclusion

- The donor is compatible with this TRG5 recipient because all TRG5 match rules are satisfied; WPCS assigns a score of MS1 (Sufficient Match) PF=10202.
- The overall degree of phenotype match across the complete antigen set is MS1 (Sufficient Match) and PF=10202.

Worked Example 5. Recipient (TRG2) and six donors

Determining the best compatibility choice and ranking donors by match score (MS Code) and Priority Factor (PF).

Case summary

Recipient: TRG2
Donors: A, B, C, D, E, F
Goal: determine the best compatible donor and produce a ranked list by degree of compatibility.

Decision rules (TRG5)

- Rank donors by MS Code (match strength) first, then by Priority Factor (PF) as the tie-breaker.
- Only donors with MS Codes MS0 or MS1 are considered compatible.
- Lower PF indicates higher priority (better choice).
- Donors with insufficient data are treated as Unknown (Null compatibility).

Table G5.1 Results table

N	Donor	Match Result				Rank
		Choice Depth	MS Code	Choice Factor	Priority Factor	
1	Donor A	7	MS0	0	10200	1
2	Donor B	7	MS1	1	10201	2
3	Donor C	7	MS1	2	10211	3
4	Donor D	7	MSM	-1	10440	N/A
5	Donor E	7	MSM	-1	10460	N/A
6	Donor F	7	Unknown	Unknown	Unknown	Null

Interpretation and rules applied

- MS0 = Exact match → highest compatibility. Donor A is MS0 and therefore ranked 1.
- MS1 = Sufficient match → acceptable but lower than MS0. Donors B and C are MS1; PF breaks the order: PF(B)=10201 is lower (better) than PF(C)=10211, so B ranks 2 and C ranks 3.

- MSM = Match excluded/incompatible → D and E are labeled incompatible and receive no rank. Between incompatible units, PF can still indicate relative risk (PF(D)=10440 < PF(E)=10460), but they remain ineligible.
- Unknown / Null → Donor F lacks the necessary phenotype/data; compatibility cannot be determined.

Conclusion

- Compatible donors: A, B, C. Best donor is Donor A (MS0, PF = 10200).
- Among compatible donors, rank order is A (1) > B (2) > C (3) based on the MS Code, then the PF.
- Donors D and E are incompatible; D is lower risk than E per PF, but neither is selectable.
- Donor F is indeterminate due to missing data.

Worked Example 6. Double red cell population (DP), Summary and Interpretation

Case: Recipient (TRG2) with double population (DP) for E: E(DP)e+; Donor: E- e+.
Decision rule (DP): donor must be E-.
Compatibility definition (TRG2): evaluated across 7 antigens: A, D, C, c, E, e, K.

Table G6.1 Per-antigen results and scoring

	Antigens						
	ABO	D	C	c	E	e	K
Recipient	A	D+	C+	c+	E(DP)	e+	K-
Donor A1	A	D+	C+	c+	E-	e+	K-
MS Code	MS0	MS0	MS0	MS0	MS1	MS0	MS0
Priority Factor (PF)	10000	100	0	0	1	0	100
Choice Depth	1	1	1	1	1	1	1
Choice Factor	0	0	0	0	1	0	0

- How PF contributions are assigned: these are the WPCS priority weights for each antigen under the match rules (given in the case).
- E (double population): recipient shows a double population for E (both E+ and E- subpopulations). Donor A1 is E-, which satisfies the DP rule ("Donor must be E-") and yields MS1 (Sufficient Match) for the E antigen; its PF contribution here is 1 (per the provided rule set).

Aggregate metrics

- Compatibility: MS1 (Sufficient Match) — recipient and donor A1 are compatible under TRG2 rules.
- Priority Factor (PF) = sum of per-antigen PF contributions
- Choice Depth: 7 (the seven antigens considered: ABO, D, K, C, c, E, e).
- Choice Factor: 1 (1 match rule deviation from MS0).

Conclusion

- The donor A1 meets the DP constraint (E-) and all other antigen requirements; the E double population in the recipient is handled by accepting an E- donor (hence MS1 for E).
- The high PF (10201) reflects the weighted importance of the antigens in the match rules.
- This donor A1 is considered a sufficient match.

Worked Example 7. Double red-cell population (DP), Recipient (TRG 2) — Donor B1 vs Donor C1

Summary

When both antigens in a pair are chimeric, donors carrying the "most hazardous" antigen are excluded. In this case, the recipient and both donors (B1 and C1) are incompatible. Donor C1 has a lower Priority Factor (PF) than donor B1; therefore, C1 represents a lower risk than B1.

NOTE: The tables below show the MS Code and PF calculations used for each donor.

Key results (high level)

- Recipient and Donor B1: incompatible
- Choice Depth: 7 (ABO, D, K, C, c, E, e)
- Priority Factor (PF): 10441 (10000 + 100 + 0 + 0 + 240 + 1 + 100)
- Match Score Code: MSM (Mismatch)
- Choice Factor: −1

- Recipient and Donor C1: incompatible
- Choice Depth: 7 (ABO, D, K, C, c, E, e)
- Priority Factor (PF): 10251 (10000 + 100 + 0 + 0 + 1 + 50 + 100)
- Match Score Code: MSM (Mismatch)
- Choice Factor: −1

Table G7.1 Recipient vs Donor B1 (double population)

	Antigens						
	ABO	D	C	c	E	e	K
Recipient	A	D+	C+	c+	E(DP)	e(DP)	K-
Donor B1	A	D+	C+	c+	E+	e-	K-
MS Code	MS0	MS0	MS0	MS0	MSM	MS1	MS0
Priority Factor (PF)	10000	100	0	0	240	1	100
Choice Depth	1	1	1	1	1	1	1
Choice Factor					-1		

Aggregate: Match Score = MSM (Mismatch); PF = 10441; Choice Depth = 7; Choice Factor = −1.

Table G7.2 Recipient vs Donor C1 (double population)

	Antigens						
	ABO	D	C	c	E	e	K
Recipient	A	D+	C+	c+	E(DP)	e(DP)	K-
Donor C1	A	D+	C+	c+	E-	e+	K-
MS Code	MS0	MS0	MS0	MS0	MS1	MSM	MS0
Priority Factor (PF)	10000	100	0	0	1	50	100
Choice Depth	1	1	1	1	1	1	1
Choice Factor						-1	

Aggregate: Match Score = MSM (Mismatch

Worked Example 8. Specific autoantibodies

Case summary

Recipient (TRG 2) has a specific autoantibody (anti-e) affecting the E/e pair. Two candidate donors (D1 and E1) are evaluated. Both donors are incompatible with the recipient under the TRG2 rules. Donor E1 represents a lower risk than donor D1 because E1 has a lower Priority Factor (PF).

Recipient (TRG 2) — Donor D1: Compatibility assessment

- Choice Depth: 7 (ABO, D, K, C, c, E, e)
- Priority Factor (PF): 25200 (10000 + 100 + 0 + 0 + 0 + 15000 + 100)
- Compatibility: MSM (Mismatch)
- Choice Factor: −1

Table G8.1 Recipient vs Donor D1 (specific autoantibodies)

	Antigens						
	A	D	C	c	E	e	K
Recipient	A	D+	C+	c+	E-	e+ *anti-e antibody*	K-
Donor D1	A	D+	C+	c+	E-	e+	K-
MS Code	MS0	MS0	MS0	MS0	MS0	MSM	MS0
Priority Factor (PF)	10000	100	0	0	0	15000	100
Choice Depth	1	1	1	1	1	1	1
Choice Factor						-1	

Aggregate: PF = 25200; MS Code = MSM (Mismatch); Choice Depth = 7; Choice Factor = −1.

Recipient (TRG 2) — Donor E1: Compatibility assessment

- Choice Depth: 7 (ABO, D, K, C, c, E, e)
- Priority Factor (PF): 10441 (10000 + 100 + 0 + 0 + 240 + 1 + 100)
- Match Score Code (overall): MSM (Mismatch)
- Choice Factor: −1

Table G8.2 Recipient vs Donor E1 (specific autoantibodies)

	Antigens						
	A	D	C	c	E	e	K
Recipient	A	D+	C+	c+	E-	e+<br>*anti-e antibody*	K-
Donor E1	A	D+	C+	c+	E+	e-	K-
MS Code	MS0	MS0	MS0	MS0	MSM	MS1	MS0
Priority Factor (PF)	10000	100	0	0	240	1	100
Choice Depth	1	1	1	1	1	1	1
Choice Factor					-1		

Aggregate: PF = 10441; MS Code = MSM (Mismatch); Choice Depth = 7; Choice Factor = −1.

Conclusion and interpretation

- Both donors (D1 and E1) are incompatible with the TRG2 recipient because the compatibility is MSM (Mismatch) in each comparison.
- D1 is higher risk than E1: D1's PF = 25200 versus E1's PF = 10441, so E1 is the lower-risk (lower-PF) incompatible donor.
