# Supplementary material for: Quantifying red blood cell compatibility beyond ABO and RhD: a recipient-centered model for matching, allocation, and inventory curation
Source: Front Med (Lausanne). 2026 Jul 14;13:1875496. doi: 10.3389/fmed.2026.1875496 (PMC13407175; doi:10.3389/fmed.2026.1875496)
Supplement: Supplementary file 9 [file Data_sheet_8.pdf]

Supplement H. Prototype System

This supplement provides a high-level overview of the IHF prototype system, including conceptual data flow and system components. These descriptions are intentionally brief because internal design is outside the scope of this article.

The supplement also illustrates how the prototype UI enables a user to retrieve precomputed WPCS results, receive patient-specific donor and unit recommendations, and view outputs from other IHF modules and IHF analytics insights and reports.

A demonstration video (Video H1) is included.
Video H1. Demonstration of the IHF prototype system.
This video illustrates how the IHF user interface (Panorama) presents integrated clinical and laboratory information, and how the system performs compatibility assessment, calculates the degree of phenotype match, and supports RBC unit selection and allocation. The recording shows the steps executed in a typical unit selection case. Duration: 3 minutes 30 seconds.

Contents

Supplement H. Prototype System 1

- H1. Overview 2
- H2. Conceptual Architecture 2
- H3. Solution Engine – Operational Module 3
- H4. Solution Engine – Analytics Module 3
- H5. User Interface: Panorama 3
- H6. Metadata-Driven Configuration 7
- H7. Technologies (Brief Note) 9

H1. Overview

The prototype system is built on the IHF integrative model, which defines its operational, analytical, and user-interface capabilities. The framework acquires patient, donor, and blood bank inventory data using defined specifications and intake mechanisms. These inputs are processed by the IHF Solution Engine, and the resulting information is displayed through the Panorama interface (Figure H1).

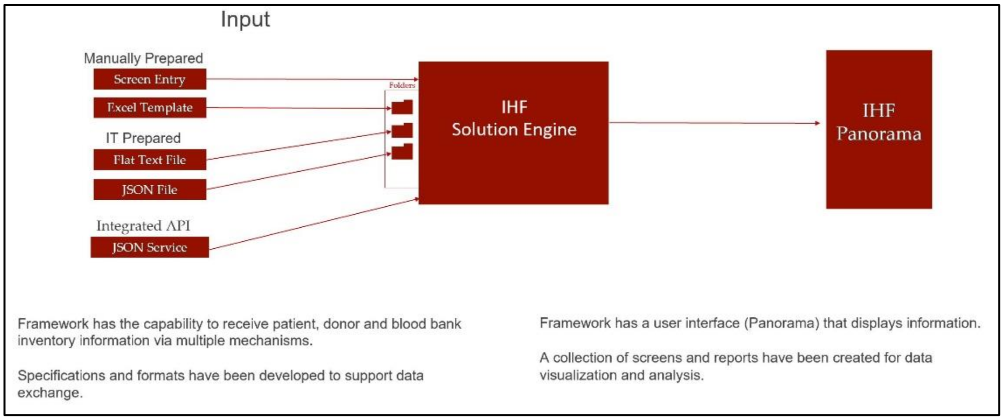

Figure H1 IHF Data Ingestion, Solution Engine, and Visualization

The system is organized into interconnected modules that collectively support the full set of IHF functions, including data processing, compatibility logic, and user-facing visualization.

H2. Conceptual Architecture

IHF architecture consists of three major components:

Specifications and Interfaces
These components define how information enters and leaves the system, including data formats and transfer mechanisms for patient, donor, and inventory sources.

Solution Engine
The Solution Engine receives, stores, and processes data. It includes:

- an operational database derived from the logical data model
- a rules engine
- software modules for profile construction and matching
- a dimensional data mart for storing operational history and aggregates

Panorama Interface
Panorama provides real-time access to operational and analytical information, including patient, donor, and inventory views, compatibility results, and historical insights.

H3. Solution Engine – Operational Module

The operational module produces the data structures required for compatibility assessment and allocation.

- Patient profiles:

The system receives demographic, clinical, and laboratory data. TRG classification is stored as metadata. Algorithms generate individual and collective patient profiles, which are stored in the operational database.

- Donor profiles:

Donor demographic and laboratory data are processed similarly. PUT classification is stored as metadata, and algorithms generate donor profiles for storage in the operational database.

- Inventory profiles:

Inventory data are processed to create unit-level profiles and an inventory snapshot.

- Matching process:

The system performs an all-patients-to-all-donors (and units) matching process. Compatibility results, rule-level details, and model recommendations are stored in the operational database.

- Data mart population:

Patient profiles, donor profiles, inventory profiles, operational snapshots, and matching results are saved to the data mart for analytics.

H4. Solution Engine – Analytics Module

The analytics module generates operational history, inventory aggregates, and snapshots of unit-selection decisions. These outputs support reporting, analysis, comparison of clinician and model decisions, and inventory optimization.

H5. User Interface: Panorama

Panorama provides a unified interface for accessing operational, analytical, metadata, and reference data.

Operational capabilities

- 360° operational snapshot
- patient, donor, and unit profiles
- compatibility results and model recommendations
- unit-selection workflow

Analytical capabilities

- daily operational history
- patient and donor historical views
- inventory analytics and optimization history
- comparisons of clinician decisions with model recommendations

Configuration capabilities

- TRG definitions
- PUT classifications
- match rules
- reference data and metadata settings

Panorama Sample Screens: Operational

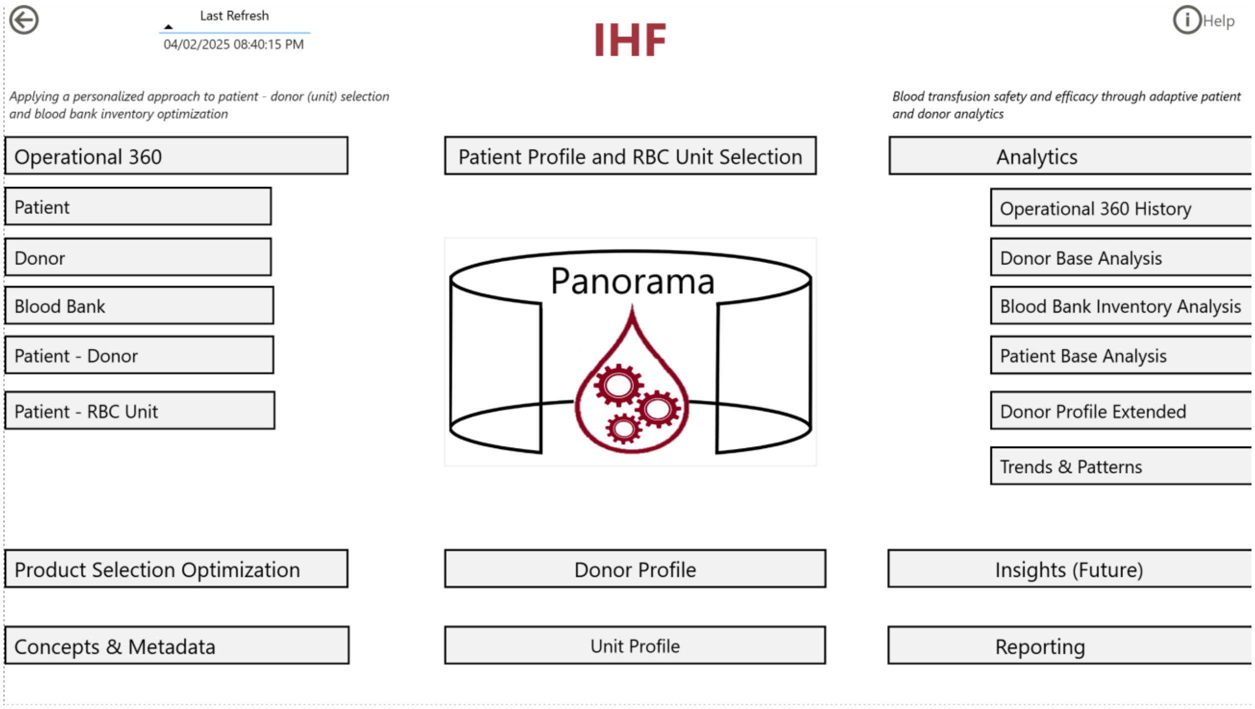

Figure H5.1 Main Menu

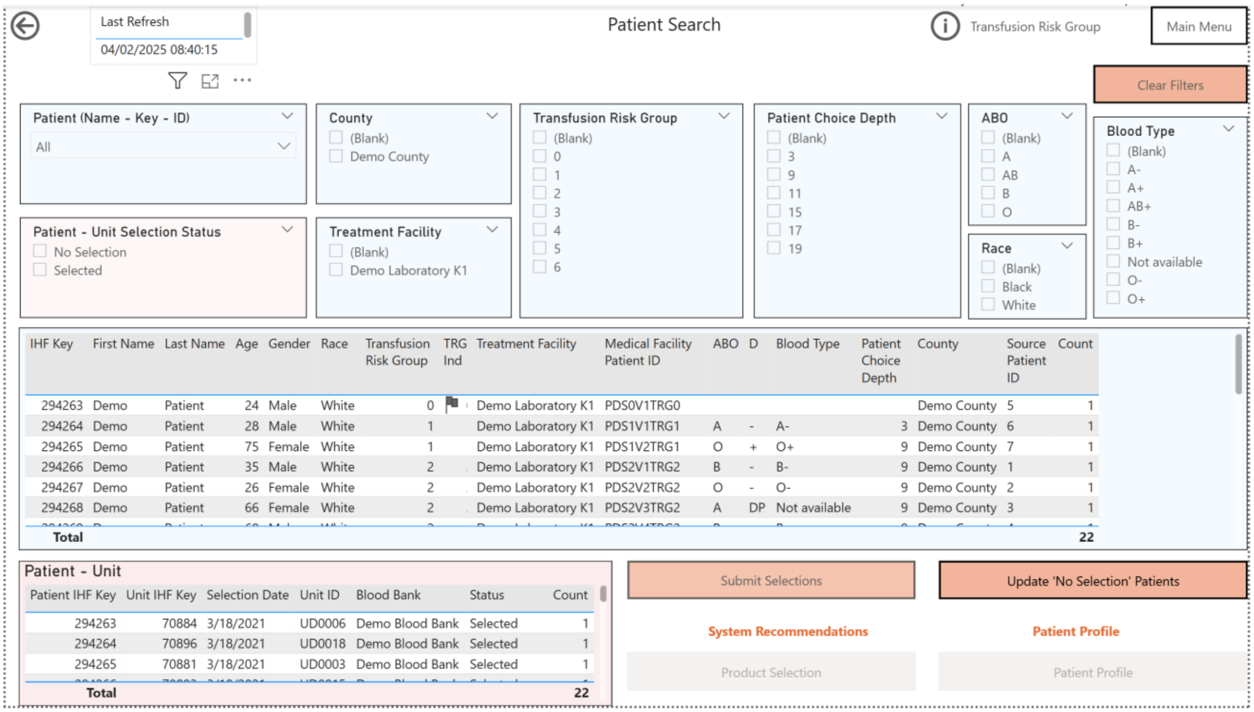

Figure H5.2 Patient Search

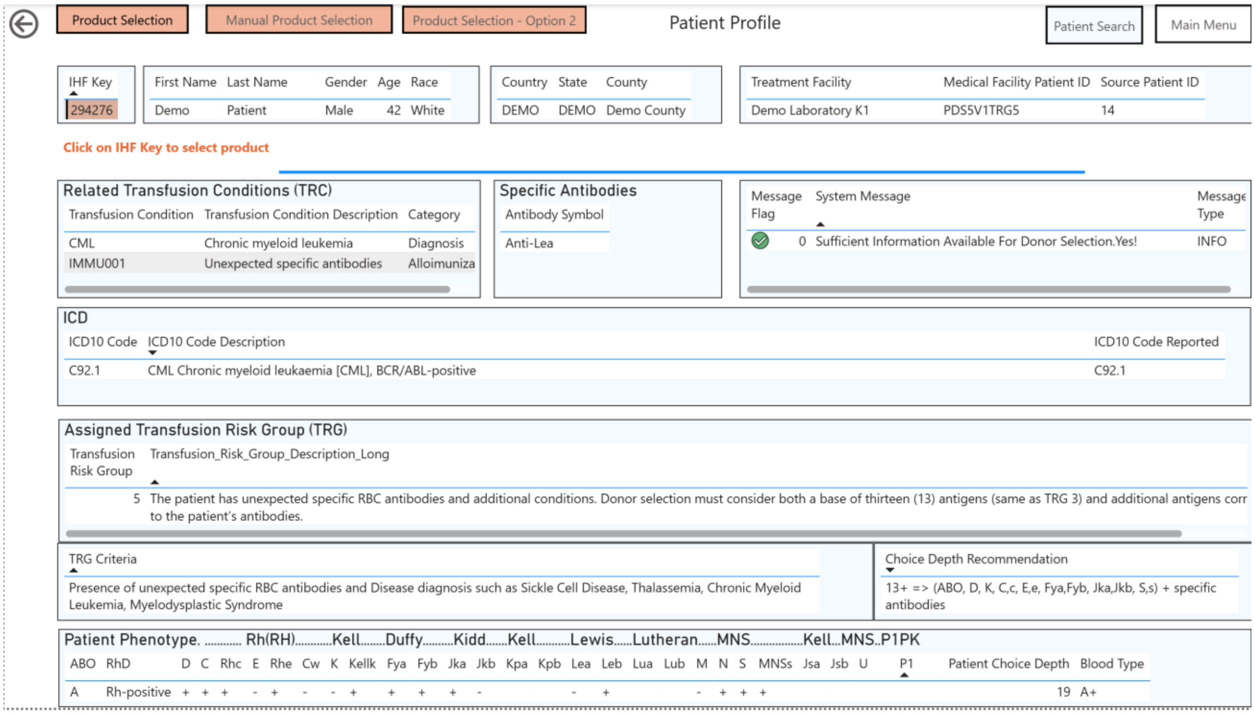

Figure H5.3 Patient Profile

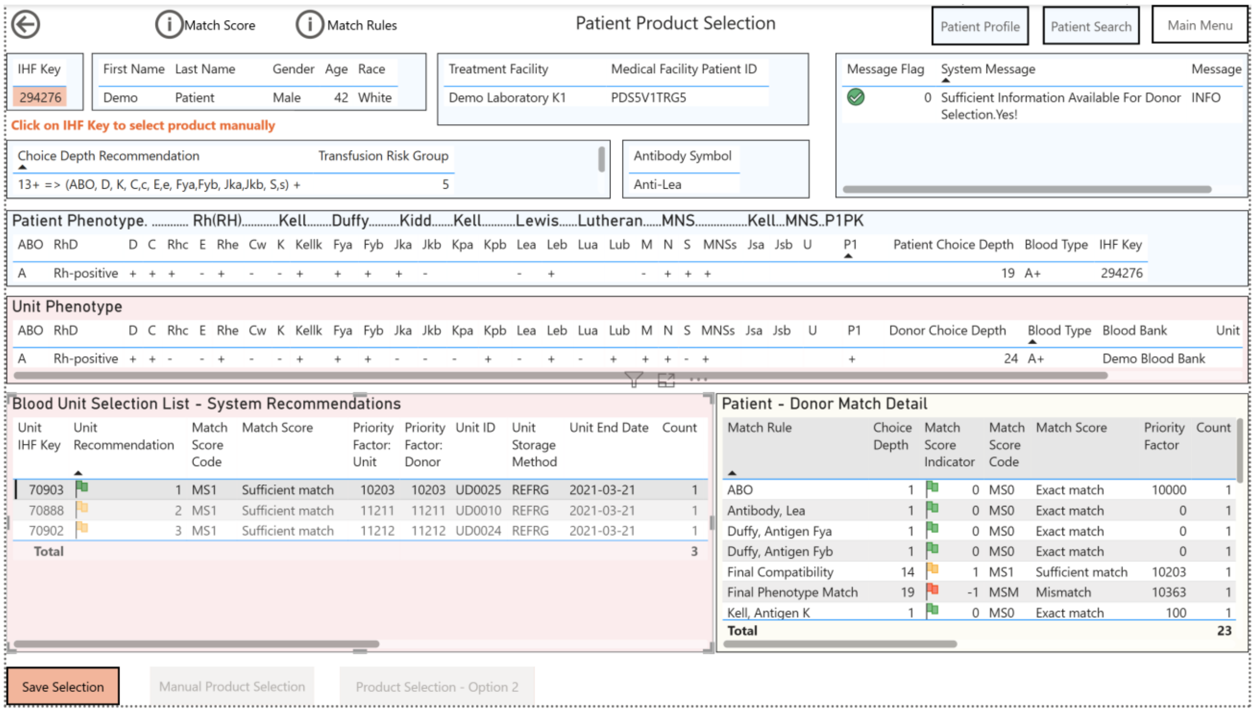

Figure H5.4 Patient-unit model recommendations

Panorama Sample Screens: Analytics

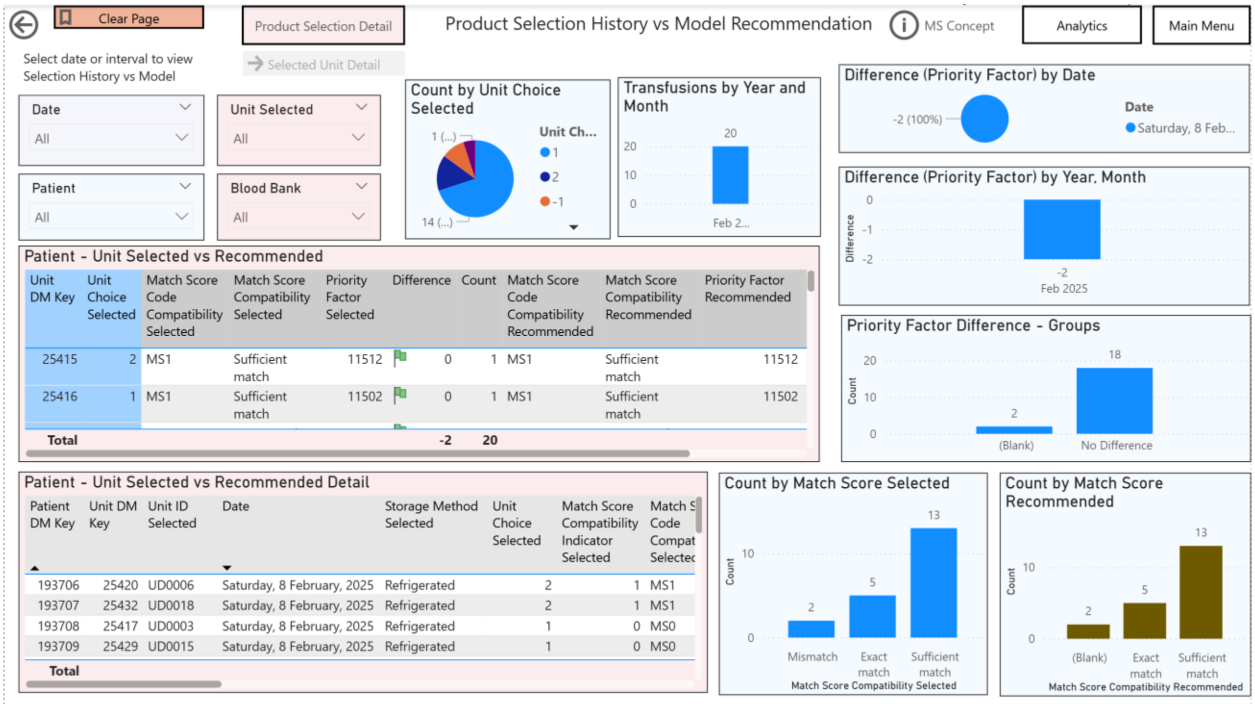

Figure H5.5 Clinician decisions vs. model recommendations

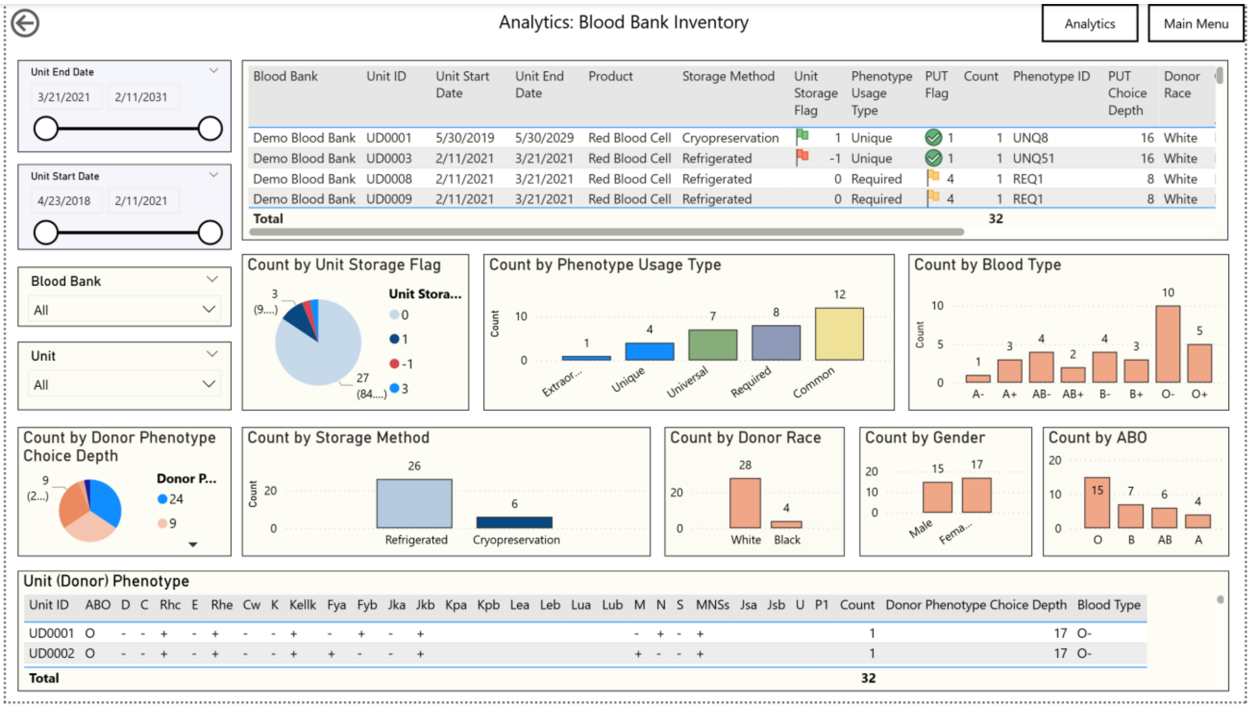

Figure H5.6 RBC Inventory Analysis

H6. Metadata-Driven Configuration

Most IHF concepts and processes are metadata-driven and configurable within Panorama, including TRG definitions, PUT classifications, priority factors, match rules, error and warning messages, and reference data. Metadata can be tailored to the standards and protocols of individual medical facilities, research laboratories, or blood banks.

The IHF uses a single, unified Priority Factor (PF) system that operates across two functional layers. Rule-level PFs weight specific compatibility conditions within the matching rules, while unit-specific PFs (e.g., Unique PUT, rare phenotypes; Table H6.1) adjust allocation desirability for individual units. Both PF types share the same PF scale and are calibrated together to ensure consistent behavior. During operational processing, the recipient–unit PF is computed as the sum of donor-level PF and unit-specific PF, aligning compatibility logic with allocation prioritization.

Table H6.1 RBC Unit-Specific Factors.

Unit-Specific Factor	Priority Factor
Cryopreserved RBC unit (Unique PUT)	300
Fresh (refrigerated) RBC unit	0
Extraordinary PUT	5000
Universal, Required, Unique, Common PUT phenotype	0

Metadata sample screens

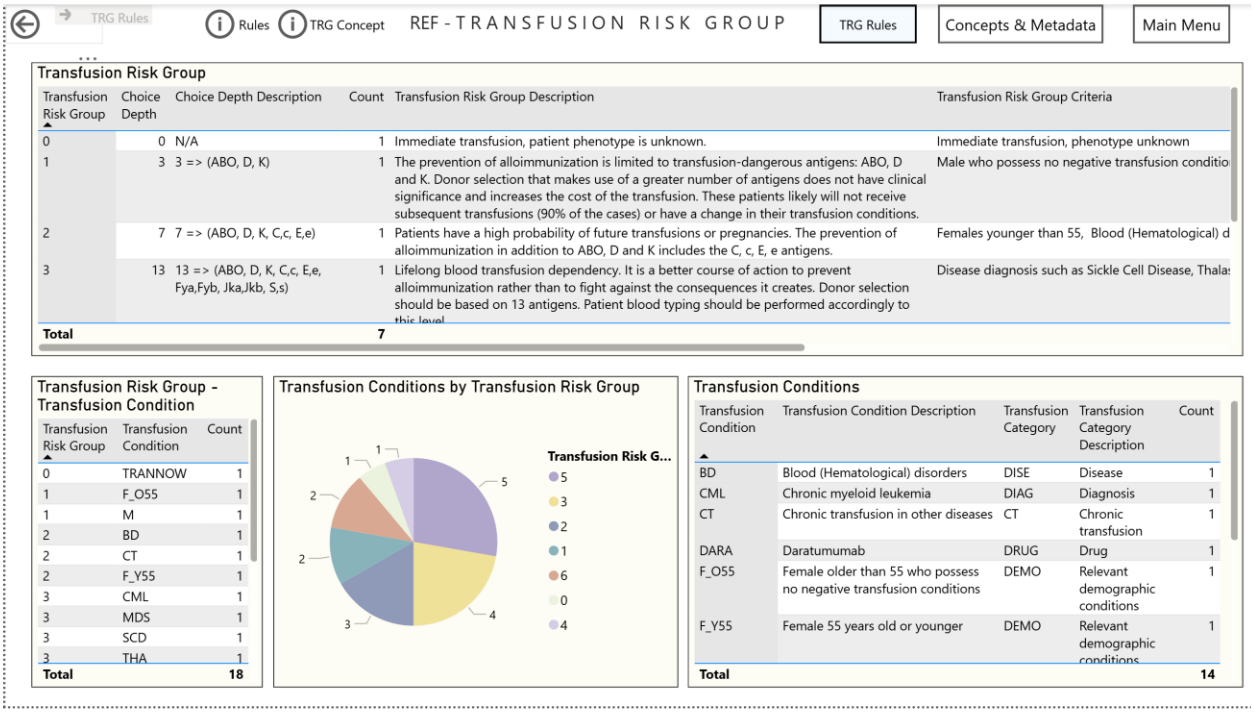

Figure H6.1 TRG Metadata

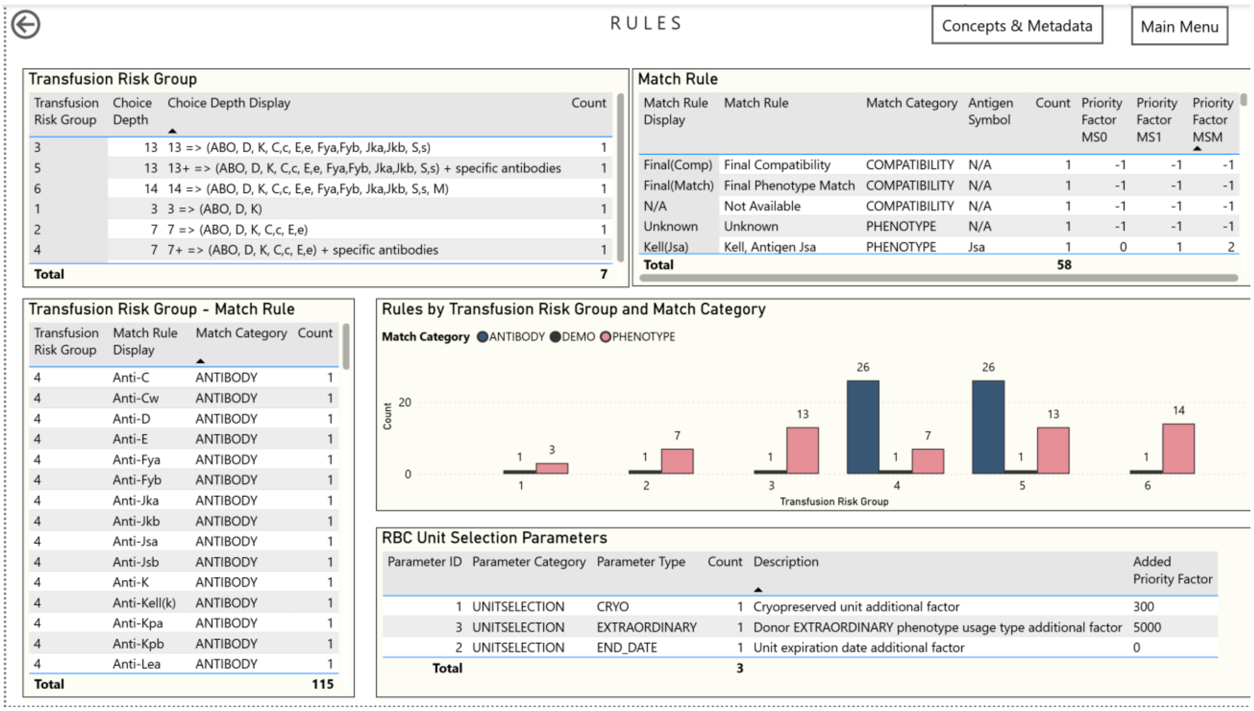

Transfusion Risk Group

Transfusion Risk Group	Choice Depth	Choice Depth Display	Count
3	13	13 => (ABO, D, K, C,c, E,e, Fya,Fyb, Jka,Jkb, S,s)	1
5	13	13+ => (ABO, D, K, C,c, E,e, Fya,Fyb, Jka,Jkb, S,s) + specific antibodies	1
6	14	14 => (ABO, D, K, C,c, E,e, Fya,Fyb, Jka,Jkb, S,s, M)	1
1	3	3 => (ABO, D, K)	1
2	7	7 => (ABO, D, K, C,c, E,e)	1
4	7	7+ => (ABO, D, K, C,c, E,e) + specific antibodies	1
**Total**			**7**

Match Rule

Match Rule Display	Match Rule	Match Category	Antigen Symbol	Count	Priority Factor MS0	Priority Factor MS1	Priority Factor MSM
Final(Comp)	Final Compatibility	COMPATIBILITY	N/A	1	-1	-1	-1
Final(Match)	Final Phenotype Match	COMPATIBILITY	N/A	1	-1	-1	-1
N/A	Not Available	COMPATIBILITY	N/A	1	-1	-1	-1
Unknown	Unknown	PHENOTYPE	N/A	1	-1	-1	-1
Kell(Jsa)	Kell, Antigen Jsa	PHENOTYPE	Jsa	1	0	1	2
**Total**				**58**			

Transfusion Risk Group - Match Rule

Transfusion Risk Group	Match Rule Display	Match Category	Count
4	Anti-C	ANTIBODY	1
4	Anti-Cw	ANTIBODY	1
4	Anti-D	ANTIBODY	1
4	Anti-E	ANTIBODY	1
4	Anti-Fya	ANTIBODY	1
4	Anti-Fyb	ANTIBODY	1
4	Anti-Jka	ANTIBODY	1
4	Anti-Jkb	ANTIBODY	1
4	Anti-Jsa	ANTIBODY	1
4	Anti-Jsb	ANTIBODY	1
4	Anti-K	ANTIBODY	1
4	Anti-Kell(k)	ANTIBODY	1
4	Anti-Kpa	ANTIBODY	1
4	Anti-Kpb	ANTIBODY	1
4	Anti-Lea	ANTIBODY	1
**Total**			**115**

RBC Unit Selection Parameters

Parameter ID	Parameter Category	Parameter Type	Count	Description	Added Priority Factor
1	UNITSELECTION	CRYO	1	Cryopreserved unit additional factor	300
3	UNITSELECTION	EXTRAORDINARY	1	Donor EXTRAORDINARY phenotype usage type additional factor	5000
2	UNITSELECTION	END_DATE	1	Unit expiration date additional factor	0
**Total**			**3**		

Figure H6.2 Rules Metadata

H7. Technologies (Brief Note)

The pilot implementation uses the Microsoft technology ecosystem, including a relational operational database and a dimensional data mart for analytics. Technology details are provided solely to contextualize the prototype and are not required to reproduce the conceptual framework.
