# Supplementary material for: Quantifying red blood cell compatibility beyond ABO and RhD: a recipient-centered model for matching, allocation, and inventory curation
Source: Front Med (Lausanne). 2026 Jul 14;13:1875496. doi: 10.3389/fmed.2026.1875496 (PMC13407175; doi:10.3389/fmed.2026.1875496)
Supplement: Supplementary file 10 [file Data_sheet_9.pdf]

Supplement I. Pilot Validation

This supplement presents the pilot validation of the IHF workflow, including compatibility assessment, degree-of-phenotype-match evaluation, recipient–unit ranking, allocation logic, and validation of the PUT concept. The pilot validates the prototype system's execution of this workflow and uses system-generated data to analytically evaluate core conceptual components and the workflow's integration logic.

Contents

Supplement I. Pilot Validation 1
- Pilot Objectives 2
- Pilot Data Overview 3
- System Generated Outputs 3
- Patient Collective Profile 3
- Donor Collective Profile 5
- Decision Quality Analysis: RBC Unit Selections vs. System Recommendations 7
- Decision Quality Analysis: by Situation Type 8
- Decision Quality Analysis: Conclusion 10
- Phenotype Usage Type Proven Concept 10
- Blood Bank Inventory Analysis 13
- Pilot Objectives Achieved 13
- Conclusion 14
- Supplemental Tables 14

Pilot Objectives

The IHF completed a pilot project in which the system processed two months of operational data (September–October 2020) from a specialized clinical unit that manages blood disorders.

Patient-Base Analysis

- ✓ Data processing and TRG assignment: Processing of patient demographics, clinical, and laboratory data, followed by assignment of each patient to a Transfusion Risk Group (TRG).
- ✓ Transfusion-relevant conditions: Assessment of blood typing information sufficiency; identification of chimerism, autoantibodies, and rare or improbable antigen combinations.
- ✓ Profile generation: Creation of individual patient profiles and a collective patient profile, with emphasis on distribution across TRGs.

Donor-Base Analysis

- ✓ PUT assignment: Assignment of each donor to a Phenotype Usage Type (PUT) based on antigen-profile characteristics and usage criteria.
- ✓ Antigen-profile characterization: Identification of rare and improbable antigen combinations within the donor population.
- ✓ Profile generation: Creation of individual donor profiles and a collective donor profile, with emphasis on distribution across PUT categories.
- ✓ Special categories: Identification of donors belonging to the Unique and Extraordinary PUT categories.

RBC Unit Selection Decision Quality Analysis

- ✓ Decision-quality assessment: Comparison of clinical blood-unit selection decisions with IHF model recommendations, with analysis of concordance and divergence.

Blood-Bank Inventory Analysis

- ✓ Unit-level processing: Processing of RBC unit information resulting in individual blood-unit profiles.

- ✓ Inventory optimization: Identification of units meeting criteria for cryopreservation.

Pilot Data Overview

The operational data used in the pilot were derived from laboratory and blood-bank records. All data were manually prepared in MS Excel files in accordance with the IHF data-exchange specifications.

The pilot dataset covers a two-month period (September 1–October 31, 2020) and includes patient, donor, and blood-bank inventory information. The dataset consisted of 109 active patients, 484 active donors, 914 RBC units, and 193 unit-selection decisions made by laboratory physicians.

System Generated Outputs

These prepared data were processed by the IHF solution engine. The engine's analytics component populated the framework data mart with operational history and optimization results, including:

- Historical daily operational snapshots and optimization recommendation: 61, September 1–October 31, 2020
- Patient individual and collective profiles
- RBC selection decisions quality analysis
- Donor individual and collective profiles
- Blood bank inventory analysis

Patient Collective Profile

The pilot included 109 patients: 58 men and 51 women, aged 1 to 89 years. Reflecting the regional population, all patients were white Europeans

Transfusion Conditions

Transfusion-relevant conditions were widespread across the patient population. Their distribution is shown in Table I1.

Table I1 The Collective Patient Profile: Transfusion Conditions

Transfusion Condition		Patients
Blood hematological disorders	(BD)	107
Daratumumab	(DARA)	21
Unexpected specific antibodies	(IMMU001)	9
Myelodysplastic syndrome	(MDS)	6
Multispecific/unidentified antibodies	(IMMU002)	6
Chronic myeloid leukemia	(CML)	3
Thalassemia	(THA)	1
Males who possess no negative conditions	(M)	1
Female 50 years old or younger	(F_Y50)	1

Specific Antibodies

Nine patients had unexpected specific antibodies. These included:

- Anti-M: 5 patients
- Anti-E: 1 patient
- Anti-Jka: 1 patient
- Anti-Lea: 1 patient
- Anti-P1: 1 patient

No patients had autoantibodies or rare/improbable antigen combinations.

Double Population (Chimerism)

Three male patients (ages 33, 35, and 55) demonstrated double populations (chimerism).

The Transfusion Risk Group at Work

All 109 patients were assigned to one of the six Transfusion Risk Groups (TRGs) by matching their transfusion-relevant conditions to TRG definitions. All six TRGs were represented.

- TRG 2: 63 patients
- TRG 6: 27 patients

These were the two largest groups.

Transfusion Risk Group	Count
1	1
2	63
3	9
4	8
5	1
6	27
**Total**	**109**

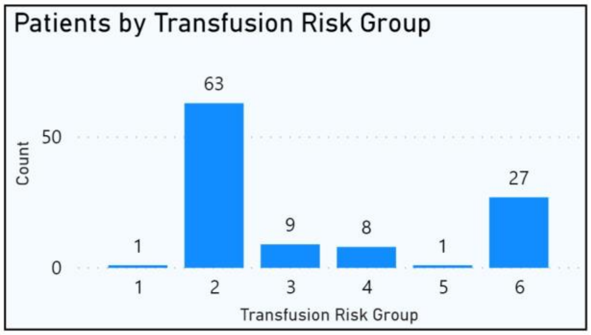

Figure I1 The Collective Patient Profile: Transfusion Risk Group

Phenotype Information Sufficiency

Phenotype sufficiency was evaluated against the antigen-matching requirements defined for each TRG. Of the 109 patients, 108 had sufficient phenotype information for their assigned group. One patient assigned to TRG 3 had incomplete phenotype data.

TRG 3 requires phenotype matching for the following antigens:
ABO, D, K, C, c, E, e, Fya, Fyb, Jka, Jkb, S, s.

The patient's available phenotype was:
AB D+ C+ c+ E+ e+ Cw– K– k+

This profile lacked results for six required antigens:
Fya, Fyb, Jka, Jkb, S, s.

Donor Collective Profile

The pilot included 484 donors: 270 men and 214 women, aged 21 to 70 years. Reflecting the regional population, all donors were white Europeans. This specialized medical unit maintains a curated donor registry to ensure availability of phenotypically appropriate donors for patients with complex transfusion requirements; as a result, the donor base does not represent a random population sample.

Phenotype

There were 64 universal donors (O–) in the donor base. The complete blood-type distribution is shown in Figure I2.

Phenotype typing depth varied across donors. The number of antigens typed ranged from 9 to 22, corresponding to Choice Depth = 9 to Choice Depth = 22. The full distribution of typing results is also presented in Figure I2.

No donors exhibited rare or improbable antigen combinations.

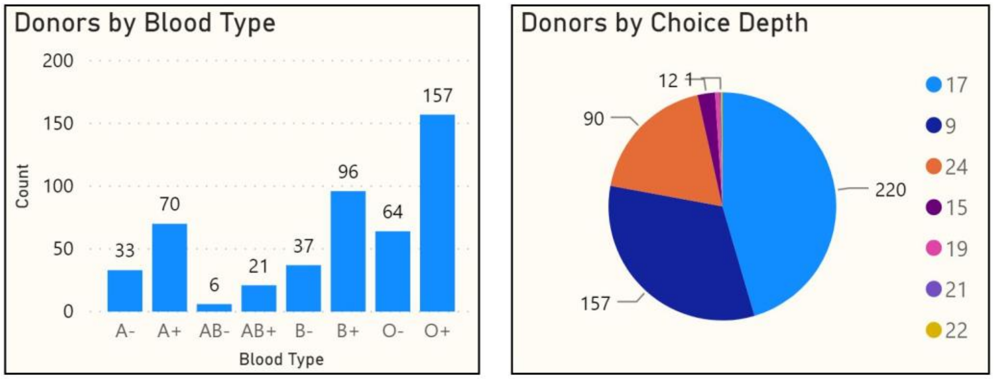

Figure I2 The Collective Donor Profile: Phenotype

The Phenotype Usage Type at Work

All 484 donors were assigned to one of the four Phenotype Usage Type (PUT) categories by matching their phenotype profiles to PUT definitions.

- Unique PUT: 15 donors (3%)
- Extraordinary PUT: 0 donors

The complete PUT distribution for the donor base is shown in Figure I3 and Table I2.

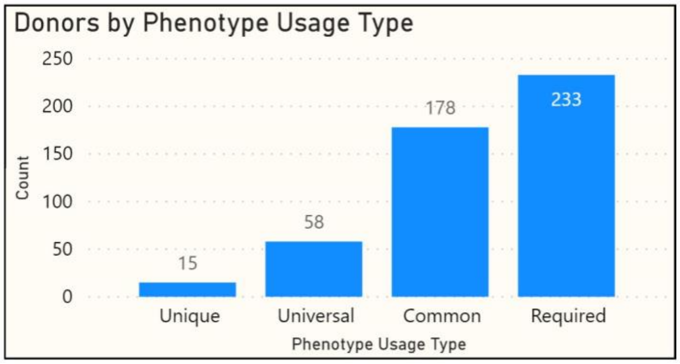

Figure I3 The Collective Donor Profile: Phenotype Usage Type

Table I2 The Collective Donor Profile: Phenotype Usage Type

Phenotype Usage Type (PUT)	Donors	Percentage
Unique	15	3%
Universal	58	12%
Common	178	37%
Required	233	48%
Extraordinary	0	0
	484	100%

Decision Quality Analysis: RBC Unit Selections vs. System Recommendations

The pilot included 109 patients. Over the 2-month pilot period (September–October 2020), 193 RBC units were selected for transfusion (133 in September and 60 in October). These 193 physician unit-selection decisions were compared with the IHF model recommendations. Differences (deviations) were quantified using the WPCS method parameters:

- Match Score (MS) Code
- Priority Factor (PF)

The results of this analysis were as follows:

1. Decisions that matched model recommendations: 114 (59%)
2. Decisions that did not match model recommendations, medium deviation (PF < 350): 34 (18%)
3. Decisions that did not match model recommendations, high deviation (PF > 350): 27 (14%)
4. Decisions that the model declined to consider (patient–unit pairs that should not be considered): 18 (9%)

The IHF pilot system screen shown in Table I3 illustrates examples of unit-selection decisions compared with model recommendations, with medium-deviation decisions highlighted.

Table I3 Unit Selection Decisions vs. Recommendations (Medium Deviation)

Unit DM Key	Unit Rank	MS Code Selected	Compatibility Selected	Priority Factor Selected	Difference	MS Code Model	Priority Factor Model	Patient DM Key
47	3	MS0	Exact Match	10500	300	MS0	10200	10
53	4	MS1	Sufficient Match	10201	1	MS0	10200	8

Operational Meaning of Deviations

Medium deviation refers to selections with a Priority Factor (PF) of 300 or lower. These cases indicate that the physician-selected unit remained within an acceptable compatibility range—or, in some instances, was at the same compatibility level but involved choosing a Unique unit instead of a Common one. Such decisions involve units that are phenotypically compatible but not optimal according to the model's ranking logic. In contrast, a high deviation (PF > 300) means the chosen unit was substantially less compatible than the model's recommended option. High-deviation cases highlight situations in which clinical judgment or availability constraints led to choices that were substantially different from the model's prioritization.

Decision Quality Analysis: by Situation Type

To complement the deviation statistics, individual unit-selection decisions were examined by scenario type. These scenarios illustrate the deviation patterns and highlight how compatibility, donor-phenotype depth, unit-specific factors, and human error contribute to decision quality.

Scenario 1: Physician-selected unit had the same Match Score and Priority Factor as the model recommendation.

> 1A. The physician and the model selected the same unit.
>
> 1B. The physician selected a different unit than the model recommended, but both units had the same Match Score and Priority Factor.

Clinically, these choices were equivalent for the patient. However, the model also incorporates donor phenotype depth (Choice Depth) and unit expiration date. From a blood bank inventory management perspective, the physician's choice was often not optimal, even though compatibility was identical.

Scenario #2: Physician-selected unit was compatible, but the model-recommended unit would have been a better choice.

2A. The physician selected an MS1 (Sufficient Match) unit, while the model recommended an MS0 (Exact Match) unit.

2B. The physician selected a unit with the same MS Code, but the model-recommended unit had a better donor Priority Factor.

2C. The physician selected a unit with the same MS Code and donor Priority Factor as the model recommendation, but the selected unit had additional unit-specific factors (e.g., classified as Unique PUT and/or cryopreserved) that made it a less desirable choice.

Table I4 illustrates Scenario 2C.

Table I4 Scenario 2C

Patient DM Key	Unit DM Key	Date	Unit Rank Selected	Priority Factor Selected	Diff.	Unit Rank Model	Priority Factor Model	Unit DM Key	TRG
22	253	10/9/20	6	10501	300	1	10201	239	2

Unit DM Key	Unit Specific Factor	Priority Factor (Added)
253	Unique PUT	300

From an inventory management perspective, the physician's choice in this case was not optimal, as it unnecessarily used a high-value Unique PUT unit when a standard unit of equal compatibility was available.

Scenario #3: Human error

A Mismatch (MSM) unit was selected despite the availability of a compatible MS1 unit, with no adverse transfusion event documented.

Table I5 provides an example.

Table I5 Human Error

Patient DM Key	Unit DM Key	MS Code Selected	Compatibility Selected	Priority Factor Selected	MS Code Model	Compatibility Model	Priority Factor Model	Unit DM Key
11739	11739	MSM	Mismatch	10642	MS1	Sufficient Match	10202	11719

Scenario #4: Selected unit not considered by the system

In this scenario, the physician selected a unit that lacked sufficient Choice Depth information and was therefore not considered by the model. Because the system excludes units with incomplete phenotype depth from ranking, the selected unit had neither a Match Score nor a Priority Factor. A compatible unit was available and was correctly recommended by the model.

This scenario highlights cases in which the physician's choice fell outside the set of eligible units for evaluation, typically because donor typing information was incomplete.

Table I6 Selected Unit not Considered by System

Patient DM Key	Unit DM Key	MS Code Selected	Compatibility Selected	Priority Factor Selected	MS Code	Compatibility Model	Priority Factor Model	Unit DM Key
362	14591	Not avail.	Not avail.	Not avail.	MS1	Sufficient Match	10202	11719

Decision Quality Analysis: Conclusion

The IHF model identifies RBC units for transfusion recipients more efficiently than current methods. Traditional workflows require substantial time and effort and also carry the risk of human error. By contrast, the IHF model evaluates all available units, applies uniform compatibility and prioritization logic, and recommends the optimal unit for each patient.

Phenotype Usage Type Proven Concept

Unique Phenotype Usage Type Concept Analytical Proof

Phenotypes classified as Unique are the most compatible with recipient phenotypes across the clinically relevant antigens of the ABO, Rh, Kell, Kidd, Duffy, and MNS blood group systems. The main manuscript presents the core Unique PUT findings; Supplement I provides the analytical details supporting that result.
Patients requiring matching for these six blood group systems are assigned to TRG 3, TRG 5, or TRG 6. In the pilot dataset, 27 patients were assigned to TRG 6, providing a sufficiently sized cohort for analysis.

We compared 327 RBC donors (out of 484 total donors) with the appropriate typing depth to the 27 TRG 6 patients. The analysis demonstrated significant differences in the number

of compatible recipients for donors classified as Unique PUT versus those classified as Common, Required, or Universal (p ≤ 0.01).

Table I7 summarizes the number of TRG 6 patients compatible with donors across the six blood group systems. Both the average and median number of compatible patients were substantially higher for Unique donors than for all other donors (Mann–Whitney test).

Table I7 TRG 6: Unique vs. Common, Required, and Universal

Donor Phenotype Usage Type (PUT)	Donors, n	Compatible patients, n		p
		Average ± standard deviation	Median (range of values)	
Unique	15	9.1 ± 2.38	9 [3 – 12]	≤0.01
Common, Required, Universal	312	3.8 ± 2.62	4 [0 - 12]	

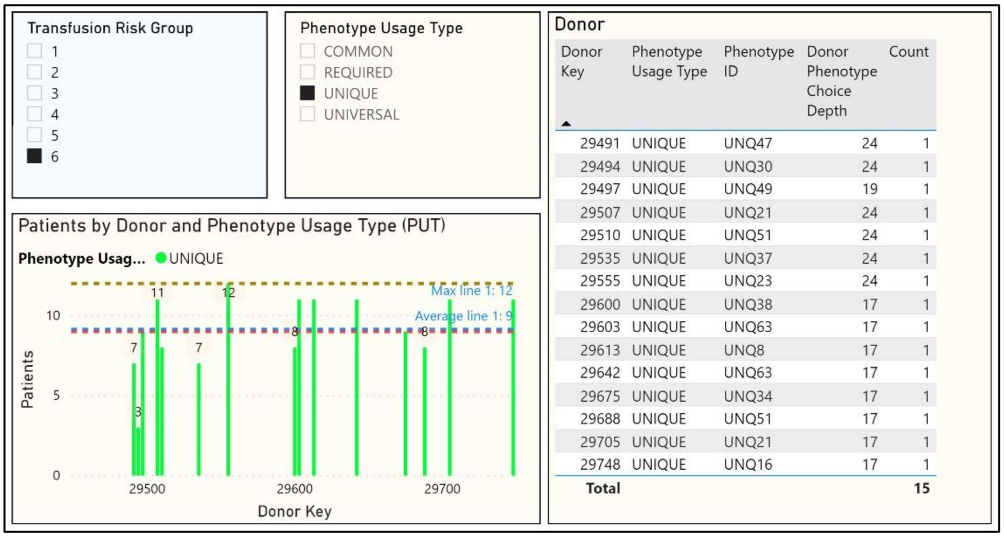

Figure I4 TRG 6: Unique PUT Donor–Patient Compatibility Chart

LEGEND: System screen from the IHF prototype displaying the number of TRG 6 patients compatible with each donor classified as Unique PUT. The chart includes individual donor compatibility counts as well as the average, median, and maximum values across the Unique donor set.

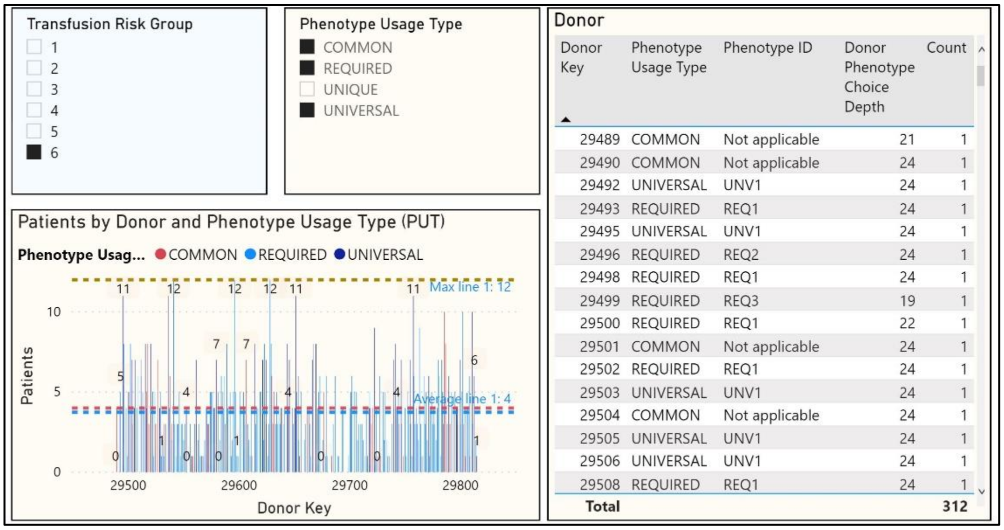

Figure I5 TRG 6: Common, Required, and Universal PUT Donor–Patient Compatibility Chart

LEGEND: System screen from the IHF prototype displaying the number of TRG 6 patients compatible with each donor classified as Common, Required, or Universal PUT. The chart includes individual donor compatibility counts and summary statistics (average, median, and maximum) for each PUT category.

Universal Phenotype Usage Type

A separate compatibility analysis was performed for TRG 2 patients, who require matching for ABO, Rh, and Kell antigens. In this cohort, 469 RBC donors were compared against 63 TRG 2 patients.

Significant differences were observed in the number of compatible patients for donors classified as Universal, Required, and Common PUT ($p \leq 0.01$). Table I8 presents compatibility results. Universal and Required donors demonstrated substantially higher compatibility than Common donors (Mann–Whitney test).

Table I8 TRG 2: Universal vs. Required vs Common PUT Donor–Patient Compatibility

Donor Phenotype Usage Type (PUT)	Donors, n	Compatible patients, n		p
		Average ± standard deviation	Median (range of values)	
Universal	58	48.0 ± 0	48 [48]	≤0.01
Required	233	24.9 ± 9.50	19 [16–40]	
Common	178	14.9 ± 8.66	14 [0–27]	

Blood Bank Inventory Analysis

In the pilot blood bank, 914 RBC units were monitored. Their Phenotype Usage Type (PUT) distribution was as follows: 724 Common, 103 Required, 73 Universal, 11 Unique, and no Extraordinary units.

The IHF approach to long-term blood bank storage optimization:

- RBC units from donors classified as Unique or Extraordinary PUT are candidates for cryopreservation.

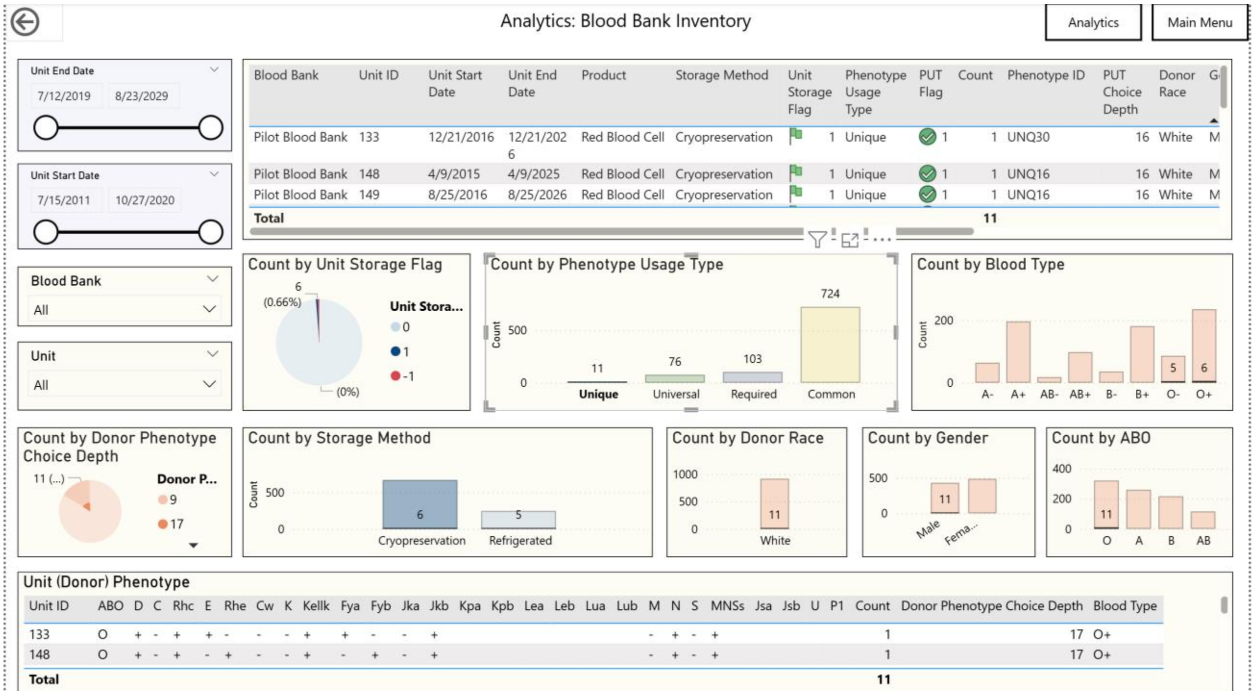

Figure I6 Unique PUT units: six cryopreserved and five refrigerated. Five refrigerated units are under consideration for cryopreservation.

Pilot Objectives Achieved

The IHF prototype project successfully achieved all defined objectives. The pilot system analyzed the medical unit's patients, donors, blood bank inventory, and physician RBC unit-selection decisions.

Key pilot achievements:

Patient classification: Active patients were classified according to the IHF Transfusion Risk Group (TRG) classification. Patient profiles were created, typing sufficiency was verified, and the TRG was validated.

Donor classification: Active donors were classified according to the IHF Phenotype Usage Type (PUT) classification. Donor profiles were created, Unique PUT donors were identified, and the PUT concept was analytically validated.

Decision-quality analysis: Physician RBC unit-selection decisions were compared with model recommendations. Laboratory researchers and immunohematologists confirmed that the IHF matching and recommendation algorithms were correct and more efficient than current manual decision-making.

Inventory optimization: RBC units from Unique PUT donors were identified for inventory planning.

Conclusion

Overall, the pilot demonstrated that the IHF framework is operationally feasible and capable of improving decision quality and inventory optimization.

PF Distribution and Supplemental Tables

Table I9. Mismatch (MSM) patient–donor pairs across TRG groups.

TRG	Patients	Patient–donor pairs	% of total pairs	MSM	MS0, MS1	Choice Factor Avg	Choice Factor Max
1	1	160	33	160	324	-1.01	-2
2	63	18362	61	18362	11880	-1.51	-4
3	9	3260	95	3260	172	-1.94	-5
4	8	2588	83	2588	528	-1.57	-4
5	1	435	-	435	-	-2.37	-5
6	27	10595	11	10595	1301	-1.88	-7

Counts of MSM pairs and associated negative Choice Factor values, shown alongside the number of compatible pairs for reference. CF quantifies mismatch (MSM) outcomes, with more negative values indicating more severe mismatches.

Residual PF distributions (excluding ABO, D, and K) were dominated by PF=0 outcomes across all TRGs, with median and IQR equal to zero and only small variability (SD ≈ 0.39–0.42). PF values in Table I10 represent antigen-rule–level outcomes (MS0 = 0, MS1 = 1), summarized by TRG for each group's antigen scope. PF=1 occurred in only 18.8–22.5% of antigen-rule evaluations.

Table I10. Residual PF Distribution by TRG (Antigen-Rule Level, Excluding ABO, D, and K)

TRG	Patients	Median PF	IQR (0.25–0.75)	SD	PF=0 count	PF=1 count	PF=1 %
1	0	0	0–0	0	0	0	0
2	63	0	0–0	0.41	37059	10461	22
3	8	0	0–0	0.39	1256	292	18.8
4	8	0	0–0	0.42	1635	477	22.5
5	0	0	0–0	0	0	0	0
6	27	0	0–0	0.41	10235	2775	21.3

Residual PF distributions at the record level (excluding ABO, D, and K) showed non-zero PF values for all TRGs with patients, with medians ranging from 1 to 2 and IQRs spanning 0–1 to 1–3. Variability remained modest (SD ≈ 0.64–1.03). PF values in Table 5 represent record-level residual PF outcomes aggregated by TRG. PF>0 occurred in 69.1–95.6% of records in TRGs 2–4 and 6, reflecting the accumulation of multiple sufficient-match (MS1) antigen-rule contributions within this compatible dataset; PF=0 appeared only in TRGs without patients.

Table I11. Residual PF Distribution by TRG (Record Level, Excluding ABO, D, and K)

**TRG**	**Patients**	**Median PF**	**IQR (0.25–0.75)**	**SD**	**PF=0 count**	**PF>0 count**	**PF>0 %**
1	0	0	0–0	0	0	0	0
2	63	1	0–1	0.7	3666	8214	69.1
3	8	2	1–2	0.8	9	163	94.7
4	8	1	0–1	0.64	133	395	74.8
5	0	0	0–0	0	0	0	0
6	27	2	1–3	1.03	57	1244	95.6
